# The microbiota–gut–brain axis in mental and neurodegenerative disorders: opportunities for prevention and intervention

**DOI:** 10.3389/fnagi.2025.1667448

**Published:** 2025-10-01

**Authors:** Lidya K. Yassin, Jurga Skrabulyte-Barbulescu, Shamsa H. Alshamsi, Sara Saeed, Shamma H. Alkuwaiti, Saif Almazrouei, Abeer Alnuaimi, Shamsa BaniYas, Dana Aldhaheri, Mahra Alderei, Safa Shehab, Mohammad I. K. Hamad

**Affiliations:** ^1^Department of Anatomy, College of Medicine and Health Sciences, United Arab Emirates University, Al Ain, United Arab Emirates; ^2^The Institute of Psychiatry, Psychology and Neuroscience (IoPPN), King's College London, London, United Kingdom

**Keywords:** microbiota–gut–brain axis, neurodegenerative diseases, microplastics, gut dysbiosis, neuropsychiatric disorders, microbiome-based interventions, neuroinflammation

## Abstract

The microbiota–gut–brain axis (MGBA) is increasingly recognized as a critical regulator of brain health, influencing both neurodevelopment and age-related neurological decline. Disruptions in this axis, driven by gut dysbiosis, have been implicated in the pathogenesis of a wide range of neurodegenerative and neuropsychiatric disorders. This review synthesizes current evidence linking microbiota alterations to Alzheimer's disease (AD), Parkinson's disease (PD), amyotrophic lateral sclerosis (ALS), multiple sclerosis (MS), and stroke—including post-stroke cognitive impairment (PSCI), as well as major depressive disorder (MDD), bipolar disorder (BD), anxiety disorders, post-traumatic stress disorder (PTSD), and chronic fatigue syndrome (CFS). Common findings include reduced microbial diversity, depletion of short-chain fatty acid (SCFA)-producing genera, and enrichment of pro-inflammatory taxa. These changes contribute to neuroinflammation, blood–brain barrier (BBB) dysfunction, microglial activation, and neurotransmitter imbalances. The review further explores the neurotoxic effects of external factors such as radiation and xenobiotics on the MGBA. Despite disorder-specific variations, shared microbial and immunological mechanisms emerge across the spectrum of conditions. Importantly, we present current and emerging strategies aimed at restoring gut–brain communication, including dietary interventions such as fiber-rich and Mediterranean diets, SCFA supplementation, probiotics, and fecal microbiota transplantation (FMT). These approaches show promise in alleviating cognitive and emotional symptoms, modulating immune responses, and potentially slowing disease progression. By integrating mechanistic insights with therapeutic perspectives, this review underscores the gut microbiota as a modifiable factor in neuropsychiatric and neurodegenerative disease. Targeting the MGBA offers a novel, translational approach to intervention that may ultimately contribute to healthier brain aging and improved outcomes across the lifespan.

## 1 Introduction

The human gut microbiota, a vast, dynamic community of microorganisms inhabiting the gastrointestinal (GI) tract, has emerged as a key modulator of brain development, function, and health. Unlike the brain, the gut microbiota is directly accessible to external influences, including dietary changes, prebiotics, probiotics, antibiotics, and other lifestyle-related interventions. This accessibility opens a promising avenue for preventive and therapeutic strategies targeting the central nervous system (CNS). The concept of the MGBA stems from extensive evidence highlighting intricate communication between the gut and the brain (Rhee et al., 2009; Cryan and O'Mahony, 2011; De Palma et al., 2014; Yassin et al., 2025; Nakhal et al., 2024). This bidirectional axis integrates neural, immune, endocrine, and metabolic pathways, enabling gut microbes to influence mood, cognition, and behavior. In turn, brain function and emotional states can modulate gut physiology and microbiota composition.

Compelling findings from germ-free (GF) animal studies have demonstrated that the absence of microbiota leads to substantial neurodevelopmental abnormalities and altered behavior (Sudo et al., 2004; Gareau et al., 2011; Heijtz et al., 2011; Neufeld et al., 2011; Clarke et al., 2013). Moreover, specific strains of bacteria have been shown to modulate behavior when administered to animals, suggesting a causal role for certain microbial populations in emotional and cognitive processes (Bercik et al., 2011; Bravo et al., 2011; Savignac et al., 2014; Desbonnet et al., 2015). In particular, microbial exposure has been shown to mitigate stress-induced behaviors and modulate immune responses, reinforcing the potential of microbial interventions in managing stress-related conditions (Bharwani et al., 2017). Even subclinical infections can induce behavioral changes in animal models without triggering classic immune activation, further supporting the functional sensitivity of the brain to microbial cues (Lyte et al., 1998). Additionally, external and iatrogenic factors such as radiation and xenobiotics are increasingly recognized as disruptors of gut–brain homeostasis, contributing to neurotoxicity and immune dysregulation.

Disruption of the gut microbiota early in life, for instance via antibiotic exposure, can result in long-lasting changes to visceral pain sensitivity and stress responsiveness, as demonstrated in rodent models (O'Mahony et al., 2014; Aleksic et al., 2023; Hamad et al., 2024a; Nakhal et al., 2025b). These findings underscore the developmental importance of early microbial signals in shaping neural circuits and behavior, processes modulated by molecules such as reelin, which controls dendritic growth and synaptic receptor function in post-natal entorhinal cortex neurons (Hamad et al., 2021b,a, 2024b,a; Leifeld et al., 2022). Mechanistically, the MGBA operates through a network of interconnected signaling systems. These include immune-mediated cytokine release, hormonal modulation through the hypothalamic–pituitary–adrenal (HPA) axis, and neural pathways involving both the enteric nervous system (ENS) and the vagus nerve (Guzzetta et al., 2022; Kasarello et al., 2023). Recent evidence demonstrates that genetic disruptions such as MECP2 loss in Rett syndrome lead to distinct, cell-type-specific alterations in dendritic architecture. These include alterations in MEC II pyramidal cell projections to the hippocampal CA1 and cortical areas, as well as stellate cells targeting the dentate gyrus and CA3 regions, both of which are involved in memory and spatial navigation (Krishnan et al., 2025). Additionally, molecular signals such as pathogen- and damage-associated molecular patterns (PAMPs and DAMPs) can cross into the circulation, potentially impacting both the microbiota and CNS function. While vagus nerve signaling has been strongly implicated in microbiota–brain communication, the full scope of neuronal networks involved remains incompletely understood. Moreover, diverse extracellular molecular signals, including neurotransmitters, neurotrophins, extracellular matrix proteins, contact-mediated ligands, and secreted diffusible cues, shape neural circuit development and modulate brain connectivity during early life (Hamad et al., 2023). These molecular signals may likewise influence gut–brain communication in adulthood.

There is increasing recognition that imbalances in the gut microbial community, referred to as dysbiosis, are associated with a range of neurological disorders. These include developmental disorders, neurodegenerative diseases, neuroimmune and metabolic conditions, as well as affective and behavioral syndromes (Zhang et al., 2021d; Góralczyk-Bińkowska et al., 2022; Loh et al., 2024). A balanced microbiome appears to be essential for healthy brain function, while microbial perturbations can contribute to cognitive deficits, mood disturbances, and neuroinflammation (Sorboni et al., 2022). Recent findings demonstrate that antibiotic-induced gut dysbiosis can also reshape dendritic architecture in adult cortical interneurons (Nakhal et al., 2025a) and stellate cells in the medial entorhinal cortex (Mydeen et al., 2025). The bidirectional nature of the MGBA further implies that not only can the brain influence gut health, but gut-targeted interventions may offer tangible neuroprotective benefits. In this review, we synthesize recent advances in our understanding of gut–brain communication with a focus on prevention and intervention strategies. We examine how microbial, environmental, and host factors interact to influence brain health across various neurological disease categories. This review explores gut dysbiosis across a broad spectrum of conditions, including neurodegenerative diseases (e.g., AD, PD, ALS), psychiatric disorders (e.g., MDD, BD, PTSD, anxiety), autoimmune syndromes (e.g., MS), and neuroinflammatory or toxic exposures (e.g., radiation, xenobiotics). Special attention is given to the translational potential of microbiome-based approaches, including dietary modification, psychobiotics, and FMT, to prevent or mitigate CNS disorders.

## 2 Neurodegenerative disorders

### 2.1 Alzheimer's disease (AD)

Evidence indicates that gut microbiota composition is altered in AD, with a trend toward reduced diversity and specific bacterial taxa linked to disease severity. The effect of MGBA arises primarily through neuroinflammatory pathways, microbial metabolites, and the modulation of systemic immune responses, influencing the brain pathology (Mulak, 2021; Thakur et al., 2023). Probiotic and dietary interventions for the restoration of microbiota balance have shown promising results in animal models and initial human trials, with some signs of cognitive improvement and reduced inflammatory markers (Kang and Zivkovic, 2021; Kesika et al., 2021). While consistent microbial alterations and mechanism pathways are emerging, evidence is preliminary; microbiome-targeted therapies are promising but require robust clinical validation.

#### 2.1.1 Background

AD is a progressive loss of memory, cognitive capabilities, speech, executive abilities, and language (Bayles et al., 2018). There are also changes in personality and behavior with the disease. AD decreases life span; the median survival rate of a person with AD is 5–9.3 years (Hegde et al., 2022; Kao et al., 2018). Many common neuropathological factors are linked with progressive AD. Neurofibrillary tangles are pathological, entangled structures in the cytoplasm of neuron cell bodies, dendrites, and axons (Metaxas and Kempf, 2016). Neuritic plaques are microscopic lesions in dendrites and terminal portions of axons. Neuritic plaques are also called senile plaques. Granulovacuolar degeneration (GVD) is a state where cells create microscopic vacuoles with granulated protoplasm (Funk et al., 2011). GVD lesions, found in regions such as the neocortex and amygdala, align with areas involved in stress and sleep regulation (Thal et al., 2011). In AD, GVD increases with disease severity, correlating with neurofibrillary lesions and memory decline, similar to β-amyloid plaques and tangles. However, the exact mechanism and the connection to Tau remain unclear (Funk et al., 2011). Reduced dendritic connections hinder neuron impulse transmission. Dopamine and cholinergic neurotransmitter deficiencies are common with all types of AD. Both white and gray matter are lost, best observed in the frontal and temporal lobes of the brain (Hegde et al., 2022; Kao et al., 2018). Increased ventricles and sulcal dilatation are a consequence of neuron degeneration within the brain. Reduced cerebral metabolism is a sign of pathological changes at the neuronal level (Wang Z. et al., 2025). When neurofibrillary tangles, neuritic plaques, and other injury occur in neurons within the brain, metabolic function is reduced (Wang Z. et al., 2025). Numerous clinical trials focusing on amyloid-beta (Aβ) and Tau proteins of AD within recent decades have been ineffective (Asher and Priefer, 2022). Existing drugs achieve little cognitive improvement and cannot halt disease progression (Bazzari et al., 2019). Because of the enigma of AD molecular mechanisms, there is an urgent need for alternative therapies acting on multiple biological pathways (Wu et al., 2024).

#### 2.1.2 Molecular and cellular mechanisms underlying the pathogenesis of AD

Amyloid plaques arise from the aggregation of Aβ peptides, particularly Aβ4_2_, which is harmful due to its tendency to form plaques and resist dissolving (Jarrett et al., 1993). The peptides are generated when the amyloid precursor protein (APP) is cleaved by BACE1 and γ-secretase (Ashe, 2020). The amyloid cascade hypothesis, which came into being as a result of the work of Hardy and Higgins in 1992, has almost since then been the dominant purview in the field of AD. According to the hypothesis, accumulation of Aβ resulting from APP triggers tau hyperphosphorylation and consequent neurodegeneration (Ma et al., 2022). Tau, which is normally a microtubule-stabilizing protein, becomes pathogenic after being hyperphosphorylated, thereby compromising neuronal transport systems. The hypothesis has expanded to include vascular dysfunction, oxidative stress, microglial activation, and impaired proteolysis (Roda et al., 2022). Nevertheless, one singular hypothesis cannot fully justify the complex etiology or mechanisms underlying AD (Joe and Ringman, 2019). Toxic Aβ oligomers activate microglia and encourage the release of pro-inflammatory cytokines (De Felice et al., 2022). Moreover, the deposit of Aβ in the vascular system may lead to the development of cerebral amyloid angiopathy, which is frequently observed in AD and involves cerebrovascular pathology (Glenner and Wong, 1984).

Insulin, a hormone produced by the pancreas β-cells, regulates glucose metabolism. When the body's tissues become less responsive to insulin, a condition known as insulin resistance, it increases the risk of type 2 diabetes mellitus (T2DM), metabolic syndrome, fatty liver disease, and atherosclerosis (Arnold et al., 2018; Lee et al., 2022). Aging often worsens this resistance through chronic high insulin levels. Emerging research links insulin resistance to AD; for instance, diabetic rats induced with streptozotocin show AD-like brain changes, including Aβ and neuroinflammation (Clark et al., 2012). While the exact connection between AD and T2DM remains unclear, insulin resistance contributes to cognitive decline. Enhancing insulin signaling in the hippocampus has shown promise in improving memory and cognition in AD models (Biessels and Reagan, 2015). Tau hyperphosphorylation and Aβ accumulation are promoted by microglial and astrocytic actions. Aβ deposits give rise to microglia-mediated neuroinflammation (Thakur et al., 2023). While the BBB tries to limit systemic inflammation, it can be compromised by cytokines such as tumor necrosis factor (TNF-α) and IL-1β. Sustained glial activation drives neuroinflammation in a vicious cycle via reactive oxygen species (ROS), cytokines, and chemokines (Thakur et al., 2023). Astrocytes, key modulators of neuroinflammation, congregate around Aβ plaques, as described in both human and mouse models. They affect amyloidosis through their role in the synthesis and disposal of Aβ, as well as through interactions with other CNS cells (Frost and Li, 2017). In reaction to AD, astrocytes experience a shift in profile, one that is reactive and pro-inflammatory while losing homeostatic roles thus disrupting BBB integrity, ion and neurotransmitter buffering, and energy metabolism (Liddelow et al., 2017; Escartin et al., 2021). Reactive astrocytes also release neurotoxic saturated lipids through the APOJ and the C3 complement component, thus affecting neuronal network activity via C3aR signaling (Lian et al., 2015; Liddelow et al., 2017). Importantly, as the chief brain source of APOE, astrocytes, especially those expressing the APOE4 allele, promote Aβ aggregation, tau pathologies, BBB breakdown, and cerebrovascular dysfunctions (Wang M. et al., 2021).

There are still important questions concerning the role of the gut microbiome in astrocyte function in AD that have yet to be studied. Of interest are which species of microbes mediate MGBA-astrocyte signaling; which astrocytic molecular pathways are modulated by the MGBA; how the MGBA-regulated astrocytic pathways contribute to AD pathology; and whether the MGBA-astrocyte could be therapeutically targeted for our benefit. On the immune front, gut microbiota can increase systemic inflammation by releasing lipopolysaccharides (LPS) and proinflammatory cytokines, and they also produce amyloids, which may stimulate neural amyloid production via immune priming. Recognition of bacterial amyloids by toll-like receptor-2 (TLR2) activates immune cells, escalating inflammation (Mulak, 2021). In the gut, T cells maintain immune balance, differentiating into proinflammatory (Th1, Th17) or anti-inflammatory [Th2, regulatory T cells (Tregs)] subsets (Dressman and Elyaman, 2022).

#### 2.1.3 The link between gut microbiome and AD

**Figure 1:**
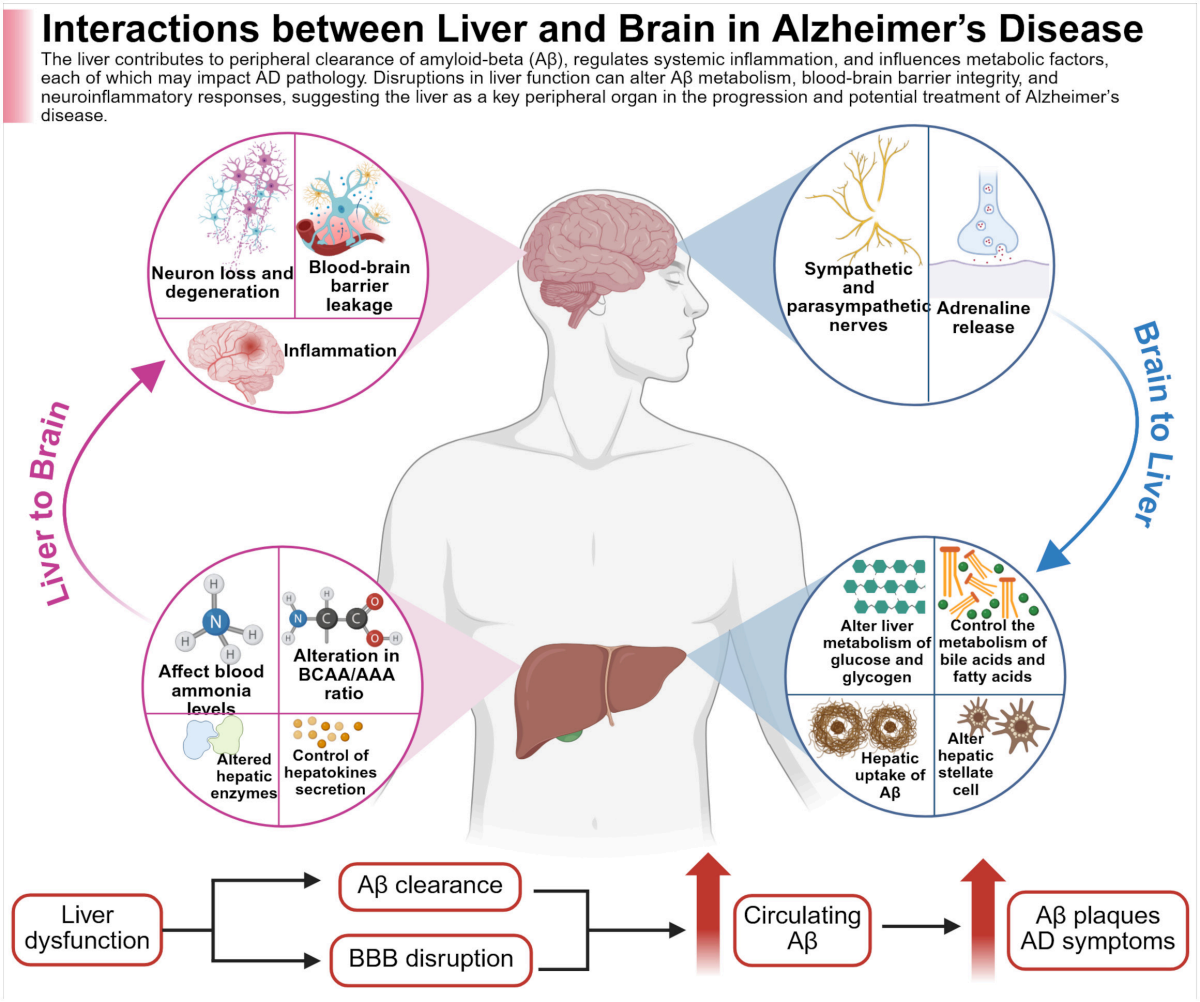
The figure illustrates the bidirectional nature of liver function, altogether with brain health, in relation to AD. The liver is involved in the clearance of circulating amyloid-beta (Aβ), the regulation of systemic inflammation, and the modulation of key metabolic processes. Disruptions in liver function may interfere with Aβ metabolism and clearance, increase BBB permeability, and trigger neuroinflammatory responses; all factors that may contribute to accelerated pathology of AD. Created with BioRender.com.

AD, based on emerging research over the past two decades, may be associated with chronic stress, where prolonged exposure to adverse life events (such as MDD and anxiety) increases the risk of developing the disease (Huang et al., 2024). The HPA axis plays a key role in this connection; it is the central component that regulates the stress response by stimulating the release of glucocorticoids from the adrenal cortex (Mulak, 2021). Elevated glucocorticoid levels, along with decreased glucocorticoid receptor (GR) function in early AD, are linked to Aβ production and abnormal tau phosphorylation (Canet et al., 2019). Additionally, GR dysregulation impacts dyslipidemia and insulin resistance. Therefore, chronic stress-induced dysregulation of the HPA axis, potentially mediated by imbalances in gut microbiota, might be a crucial link between dysbiosis and the progression of AD (Figure 1).

#### 2.1.4 Bile acids (BAs) as modulators in Alzheimer's brain-gut axis

**Figure 2:**
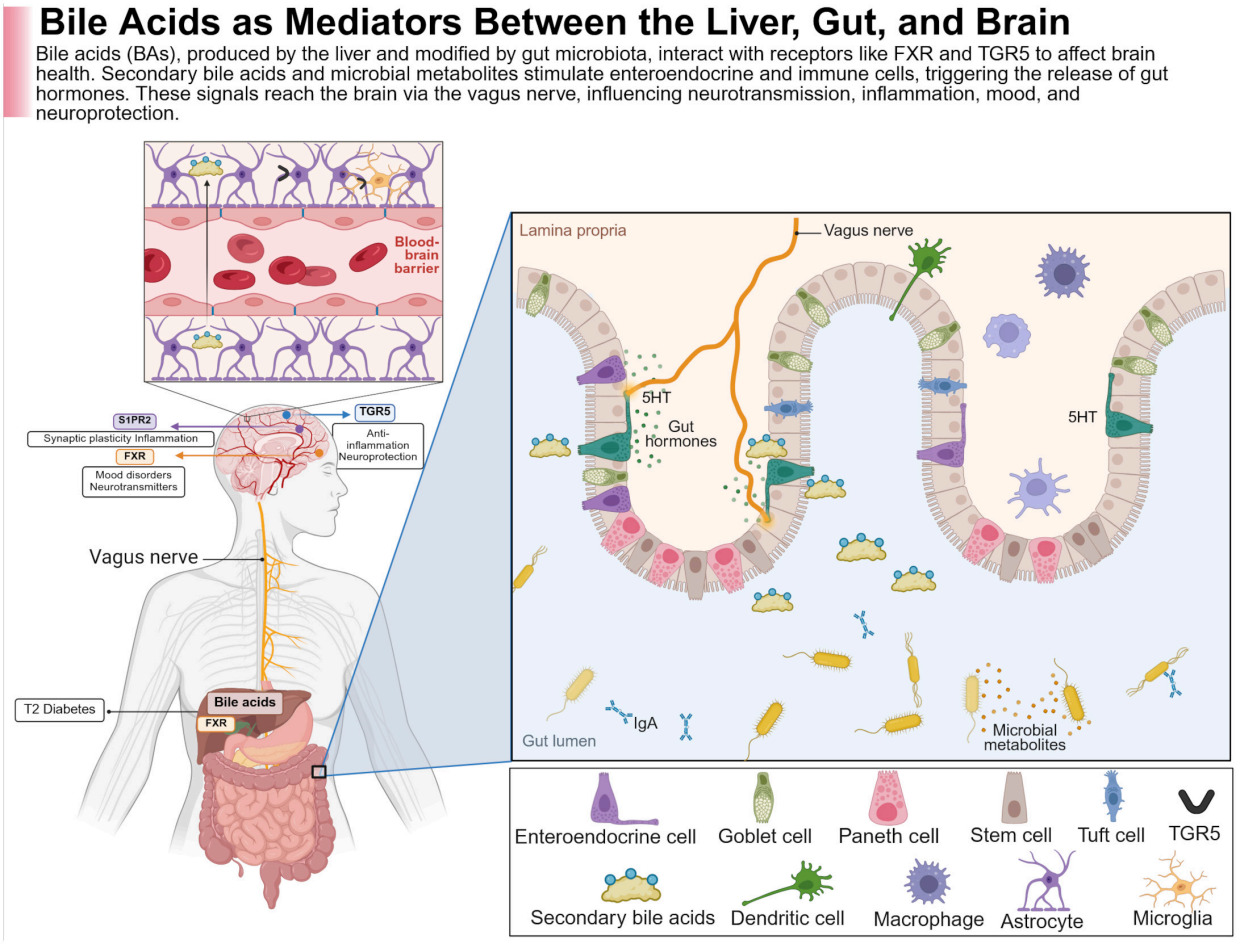
BAs provide neuroprotection through a complex system of physiological mechanisms. This figure illustrates how BAs are derived from the liver, transformed by gut microbiota, and interact with receptors such as the Farnesoid X receptor (FXR) and G protein-coupled bile acid receptor (TGR5) along the intestinal and brain axis. The modulation of microbial metabolites and secondary BAs within the gut lumen affects enteroendocrine and immune cells (e.g., dendritic cells, macrophages), leading to the secretion of gut hormones, with serotonin (5-HT) being the most prominent. These signals then travel via the vagus nerve to the CNS, where they influence processes such as synaptic plasticity, neurotransmission, inflammation, and neuroprotection. Regarding FXR, which interacts with BAs in the liver, it acts as a mediator of metabolic diseases like type 2 diabetes along the liver–gut–brain axis. In the brain, BA signaling through receptors like TGR5 and S1PR2 plays a role in mood regulation, cognition, anti-inflammatory responses, and neuroprotection, all of which are vital in neurodegenerative diseases such as Alzheimer's. Created with BioRender.com.

A potential therapeutic approach for AD consists of the BAs, which are produced in the liver (Grant and DeMorrow, 2020). Although their largest involvement is in nutrient and metabolic regulation, around 10% of them might cross the BBB, as shown in animal studies with mechanistic details (Monteiro-Cardoso et al., 2021). A comparison of 119 patients suffering from AD with 267 controls found that alterations in serum BAs correlate with cerebrospinal fluid (CSF) Aβ1–42, tau, and p-tau. Higher concentrations of glycochenodeoxycholic acid, glycodeoxycholic acid, hyodeoxycholic acid, and certain bile acid ratios were associated with patient CSF tau and p-tau. Higher hyodeoxycholic acid and certain ratios were associated with lower CSF Aβ1–42. While these associations suggest a link between serum bile acids and AD pathology, a causal relationship has yet to be established (Nabizadeh et al., 2024). BAs act in the brain by interacting with key receptors on neurons and glial cells-Farnesoid X receptor (FXR), Takeda G protein receptor 5 (TGR5), GR, and sphingosine-1-phosphate receptor 2 (S1PR2; Kiriyama and Nochi, 2019; Figure 2). BAs may modulate gut microbiota through antimicrobial properties, with deconjugated BAs produced by bacterial bile salt hydrolases being much less toxic to gut bacteria. In animal models of neurodegenerative disease, activation of TGR5 receptor was neuroprotective, decreasing neuronal death and inflammatory response (Monteiro-Cardoso et al., 2021). S1PR2, located both in the liver and in the brain, activates the ERK and AKT pathways that regulate cell survival, inflammation, and stress response in the CNS. In the liver, S1PR2 activates pro-insulin signaling through enhancing insulin-degrading enzyme (IDE), thereby potentially alleviating insulin resistance associated with T2DM and AD based on animal mechanistic studies (Vettorazzi et al., 2017; Zangerolamo et al., 2022). Beyond directly influencing pathological changes in the AD brain, BAs may provide neuroprotective benefits by modulating the microbiota–gut–brain (MGB) axis, which supports memory function. GM also regulates BA synthesis; both antibiotic use and germ-free conditions suppress the expression of CYP7A1 and CYP27A1, key enzymes involved in BA production based on animal mechanistic studies (Chiang and Ferrell, 2019). Deconjugated BAs, which are less toxic to gut microbes, are reduced in AD, likely due to decreased BSH-secreting bacteria like *Clostridium* and *Bifidobacterium*, as observed in human associative studies (Mulak, 2021). While these findings support the hypothesis that restoring BA–microbiota interactions could influence AD symptoms, causal evidence in humans remains limited and warrants further investigation. Studies consistently show altered MGBA composition in AD patients, suggesting a possible role in disease onset and progression, as shown in Table 1. Confounding variables, such as diet and environmental exposures, complicate comparisons.

**Table 1:** Summary of studies concerning the alterations of the gut microbiota in AD.

**Experimental subject**	**Main findings**	**References**
APP/PS1 mice	↑Proteobacteria, ↑Erysipelotrichaceae, and ↑Firmicutes	Bäuerl et al., 2018
	Altered gut microbial diversity with ↑Proteobacteria and Verrucomicrobia; and ↓SCFA	Zhang et al., 2017
	↓Microbial diversity, ↓spatial memory, ↑*Odoribacter* and *Helicobacter* (genus level), and ↓*Prevotella*	Shen et al., 2017
	↓Aβ plaques, ↓plaque-localized glial reactivity, and significantly altered microglial morphology	Minter et al., 2016, 2017
	AD is associated with shifts of the gut microbiome toward profiles that resemble those seen in inflammatory disorders	Bäuerl et al., 2018
	Altered peripheral inflammatory mediators in antibiotic-treated Tg mice	Minter et al., 2016
C57BL/6 mice	Infection with the pathogenic bacteria *Citrobacter rodentium* resulted in stress-induced memory disturbances	Gareau et al., 2011
Symptomatic Tg2576 mice	↑Firmicutes, ↑Bacteroidetes, and ↑*Lactobacillus*	Honarpisheh et al., 2020
Post-mortem AD brains	LPS and *Escherichia coli* K99 colocalize with Aβ in plaques and perivascular aggregates	Zhan et al., 2016
	LPS accumulates in neocortical neurons in AD	Zhao et al., 2017
	16S rRNA sequencing shows increased bacterial populations	Emery et al., 2017
Living AD human participants	↑Pro-inflammatory *Escherichia/Shigella*, abundance of anti-inflammatory *Eubacterium rectale*; ↓*Bacteroides fragilis* in AD	Cattaneo et al., 2017
	↓Microbial diversity; ↓Firmicutes, ↓*Bifidobacterium*; and ↑Bacteroidetes in AD	Vogt et al., 2017
	↑Actinobacteria; ↑Enterococcaceae; ↑Lactobacillaceae, ↑Ruminococcaceae; ↓Bacteroidetes, ↓Lachnospiraceae, ↓Veillonellaceae	Zhuang et al., 2018
	Compared to controls, individuals with AD showed ↓Clostridia, ↓Lachnospiraceae, and ↓Ruminococcaceae, along with ↑Proteobacteria and ↑Enterobacteriaceae	Liu et al., 2019
	AD patients exhibited ↑*Bifidobacterium, ↑Blautia, ↑Dorea, ↑Escherichia, ↑Lactobacillus*, and *↑Streptococcus*, while showing ↓*Alistipes, ↓Bacteroides, ↓ParaBacteroides, ↓Paraprevotella*, and *↓Sutterella* compared to controls	Li et al., 2019
	AD patients showed ↑*Alistipes, ↑Bacteroides, ↑Barnesiella, ↑Collinsella, ↑Odoribacter*; and *↓Eubacterium, ↓Lachnospiraceae Clostridium*, and *↓Roseburia* compared to controls	Haran et al., 2019
	CSF TMAO levels are ↑ in AD and positively correlate with CSF biomarkers of amyloid accumulation, tau pathology, and neuronal damage	Vogt et al., 2018
	Significant difference in the gut microbial genotypes between the AD and control human populations	Paley et al., 2018

SCFA, short-chain fatty acids; Aβ, amyloid-beta; Tg, transgenic; LPS, lipopolysaccharide; rRNA, ribosomal RNA; CSF, cerebrospinal fluid; TMAO, trimethylamine N-oxide; ↑, Increase; ↓, Decrease.

#### 2.1.5 Therapeutic interventions

##### 2.1.5.1 Probiotics

Probiotics are supplements made of live, helpful bacteria, mainly *Lactobacillus* and *Bifidobacterium*, that support gut health. They are used to correct imbalances in the MGBA and may help slow disease progression (Kesika et al., 2021). Animal studies suggest that strains like *Lactobacillus helveticus, L. plantarum*, and *L. fermentum* can improve memory and cognitive function in AD models (Kesika et al., 2021), as shown in Table 2. Similarly, *Bifidobacterium breve* A1 has been shown to reduce brain inflammation and suppress harmful immune responses triggered by amyloid buildup in the hippocampus (Kobayashi et al., 2017).

**Table 2:** Application and therapeutic effect of multiple probiotics in different animal models.

**Probiotic**	**Duration**	**Sample size**	**Results**	**References**
*Akkermansia muciniphila*	6–7 months	6–7 months old wild-type zebrafish (*n* = 100)	Improving glucose levels and diabetes markers in diabetic-AD zebrafish, while also easing AD symptoms and enhancing memory, mood, and social behavior	Qu et al., 2023
*Bifidobacterium breve*	12 weeks	16-weeks old male APPswe/PS1dE 9 mice (*n* = 8)	Strengthening the gut barrier, reduced neuroinflammation and synaptic dysfunction, improved gut microbiota, and alleviated cognitive decline in APP/PS1 mice	Zhu G. et al., 2023
*Bifidobacterium breve strain A1*	6 days	10-week-old male ddY mice (*n* = 11/12)	This probiotic reversed behavioral impairments in memory tests, and its non-living components or metabolite acetate partially improved cognitive decline in AD mice	Kobayashi et al., 2017
*Bifidobacterium breve*	6 weeks	8-weeks old male C57BL/6J mice (*n* = 8)	Enhancing synaptic plasticity and increasing BDNF, thereby slowing AD progression	Zhu et al., 2021
*Bifidobacterium longum*	4 and 8 weeks	6-months, 18 months old male C57BL/6 and 5 × FAD-Tg mice (*n* = 6)	Modifying the gut microbiota in 5 × FAD-Tg and aged mice reduced fecal and blood LPS, suppressed NF-κB and TNF-α, boosted colon tight junction proteins, and decreased Aβ production, related enzymes, and accumulation in the hippocampus	Lee et al., 2019b
*Bifidobacterium bifidum* and *Bifidobacterium longum*	30 days	3-months old C57BI/6 and 5 × FAD mice (*n* = 10)	Inhibiting amyloidosis and apoptotic processes by improving neuroinflammatory responses and ameliorating cognitive and memory deficits in AD mice	Kim H. et al., 2021
*Bifidobacterum bifidum, Lactobacillus plantarum* and exercise	8 weeks	8-weeks old male Wistar rat (*n* = 5)	Improving Aβ plaque deposition and reducing brain cell death in the brains of AD mice	Shamsipour et al., 2021
*Bifidobacterium lactis*	45 days	4-months old APP/PS1 mice (*n* = 11/12)	Reducing the deposition of Aβ plaque in the whole brain, preventing the imbalance of intestinal flora, and alleviating the cognitive impairment of APP/PS1 mice	Cao et al., 2021
*Lactobacillus plantarum*	12 weeks	8-week-old male C57BL/6J, 6-month-old male wild-type, and PrP hAβPPswe/PS1Δ E9 transgenic mice (*n* = 15/30)	Improving cognitive deterioration, reducing the level of Aβ in the hippocampus, protecting the integrity and plasticity of neurons, and inhibiting the synthesis of TMAO	Wang Q.-J. et al., 2020
*Lactobacillus plantarum*	12 weeks	6-weeks old SPF grade Wistar male rats (*n* = 8)	Improving cognitive impairment and anxiety-like behavior in AD rats, reducing neuronal degeneration and Aβ buildup, and suppressing microglial activation and neuroinflammation	Wang Y. et al., 2022

AD, Alzheimer's disease; LPS, lipopolysaccharide; Aβ, amyloid-beta; BDNF, brain-derived neurotrophic factor.

##### 2.1.5.2 Prebiotics

Prebiotics are non-digestible fibers like oligosaccharides and polysaccharides that feed good gut bacteria. These compounds not only help beneficial microbes survive but also support the production of gut-derived metabolites important for brain and gut health (Kang and Zivkovic, 2021). For instance, lactulose encourages the growth of healthy gut bacteria and may protect brain function in AD models (Lee H. J. et al., 2021). Dietary fibers such as fructo-oligosaccharides (FOS) and galacto-oligosaccharides (GOS) can enhance the gut's production of secondary BAs and SCFAs, both of which support intestinal integrity and may reduce neuroinflammation (Salminen et al., 2021). FOS, found in fruits and vegetables, is especially effective in promoting *Bifidobacterium* and *Lactobacillus* growth. In AD animal models, FOS helped preserve gut microbial balance, reduced brain cell death, and decreased the buildup of harmful proteins, such as tau and Aβ1–42 (Chen et al., 2017). These protective effects are thought to involve the MGBA, including modulation of the glucagon-like peptide-1 (GLP-1) receptor pathway, which influences both brain and metabolic health (Sun et al., 2019a). While human trials show more modest effects, prebiotic intake has been associated with subtle improvements in gut microbiota composition and immune-related gene expression, especially in older adults.

##### 2.1.5.3 FMT

FMT is performed by transferring stool from healthy donors into a recipient's GI tract to restore microbial diversity (Allegretti et al., 2018). Currently, FMT is officially approved only for the treatment of recurrent *Clostridium difficile* infection. However, the feasibility of using it to treat metabolic disorders and neurodegenerative diseases is currently under investigation in clinical trials (Liu Y. et al., 2020). FMT induces cognitive improvement while lessening the accumulation of Aβ in the brain, as shown in animal models of AD, as shown in Table 3. However, there are several issues with FMT as follows: biological mechanisms remain undescribed, donor stool will not always be available, risks and side effects remain unclear, and long-term safety data are extremely limited (Liu Y. et al., 2020). Future research will have to focus on developing areas such as formalizing procedures, ensuring safety protocols, and establishing reliable stool banks. Therefore, recommendations for incorporating FMT into standard care for AD cannot yet be made.

**Table 3:** Fecal microbiota transplantation (FMT) may enhance cognition and Aβ regulation, as suggested by findings from human and animal clinical studies.

**References**	**Sample size**	**Donor**	**Recipient**	**Results**
Hazan (2020)	Case study (*n* = 1)	85-year-old woman	82-year-old man with recurrent AD	↑Cognitive function ↑Memory ↑Mood
Park et al. (2021)	Case study (*n* = 1)	27-year-old healthy man	90-year-old woman with AD	↑Cognitive function ↑Microbiota α Diversity ↑Microbiota β diversity
Kim N. et al. (2021)	Mice (*n* = 8)	5 × FADmice	C57BL/6 mice	↓Adult hippocampal neurogenesis and BDNF expression ↑Microglia activation
Sun et al. (2019b)	Mice (*n* = 8)	WT mice	APPswe/PS1dE9 transgenic (Tg) mouse model	↓Amyloid brain deposition (Aβ 40 and Aβ 42) ↓Tau protein phosphorylation ↑Synaptic plasticity ↑SCFA and microbiota composition
Dodiya et al. (2019)	Mice (*n* = 9)	Age-matched APPPS1-21	ABX-treated APPPS1-21 male	↓Aβ pathology ↑Microglial physiology
Valeri et al. (2021)	Mice (*n* = 10)	Either 4 months old or 1 year old wild type mice	5 × FAD mice (4-month old)	↑Enterobacteriaceae, Lactobacillaceae, ↓Firmicutes ↑Plaques in the dentate gyrus and prefrontal cortex

AD, Alzheimer's disease; BDNF, brain-derived neurotrophic factor; Aβ, amyloid beta; SCFA, short-chain fatty acid; ABX, antibiotic cocktail; ↑, Increase; ↓, Decrease.

##### 2.1.5.4 Dietary intervention

Diet strongly influences gut microbiota composition and activity, depending primarily on the nutrients we eat. The Mediterranean diet (MeDi), rich in fruits, vegetables, legumes, and whole grains, has been shown to lower the likelihood of cognitive decline and delay the onset of AD by 1.5–3.5 years (Lourida et al., 2013). This diet alters gut microbiota profiles, such as higher population levels of Clostridium cluster XIVa, Lactobacilli, and Bifidobacteria, and lower levels of Firmicutes and Proteobacteria. MeDi can further influence gut bacterial diversity and functionality, generating healthy metabolites, like SFCAs, which offer multifaceted benefits for the intestinal, metabolic, and immune health of the host organism (Nagpal et al., 2019). A high fiber diet further aids in better blood glucose control among T2DM and AD subjects, possibly mediated through impacts on hemoglobin A1c and gut hormone GLP-1 levels (Zhao et al., 2018). Omega-3 fatty acids ingested from foods like sardines, walnuts, seaweed oil, and deep-sea fish provide neuroprotective effects by supporting neuronal development and synaptic activity while reducing inflammation over the gut–brain axis (Latifi et al., 2016).

##### 2.1.5.5 Exercise

Research in humans shows that regular physical exercise helps protect the brain from age-related cognitive decline and reduces the risk of AD (Meng et al., 2020). This protection is likely due to increased growth of new neurons in the hippocampus, improved signaling of brain-derived neurotrophic factor (BDNF), better synaptic function, and reduced brain inflammation (Nichol et al., 2008). Emerging evidence suggests that the MGBA might also play a key role in these effects. Both human and animal studies have shown that exercise changes the diversity and composition of gut bacteria. Microbiota changes in people, however, are heterogeneous, with food intake being one major determinant of gut microbial composition that might confound the apparent exercise-related effects (Zhou et al., 2024). In a key study by Masumoto and colleagues, 5 weeks of exercise in mice changed their gut microbiome and increased levels of butyrate (Matsumoto et al., 2008). Because exercise improves brain function and gut microbiome composition, its brain-protective effects in AD may be partly mediated through the microbiome. To better understand this relationship, future studies should compare AD mice models with disrupted gut microbiomes to those with healthy microbiomes during exercise interventions (Chandra et al., 2023). If the microbiome is confirmed as a key link, therapies that mimic an “exercise-enhanced” gut microbiome might offer a new strategy to alleviate AD symptoms.

##### 2.1.5.6 Sleep

Sleep and circadian rhythm disturbances are among the hallmark features associated with AD (Musiek et al., 2015). Almost all AD patients exhibit erratic sleep patterns, nocturnal staying awake, and daytime sleepiness (Hatfield, 2004). Notably, adults without cognitive impairment reporting a bad night's sleep are more likely to show signs of amyloid buildup in their brains observed by PET scans (Spira et al., 2013). Research has shown that Aβ levels vary with sleep-wake cycles. For example, during wakefulness, Aβ levels increase and decrease during sleep in mice, and this phenomenon is similar in humans with AD (Kang et al., 2009; Huang, 2012). Reducing Aβ plaque counts in mice elicits an improvement in sleep cycle restoration, as well as natural fluctuations in Aβ levels, thereby implying that sleep may be disturbed directly by Aβ deposition (Roh et al., 2012). Sleep deprivation is associated with increased brain activity, thereby leading to increased production of Aβ and subsequently worsening the already existing AD pathology (Lim et al., 2013). Sleep disruption changes the microbiome, as is shown in experiments where alterations in gut bacterial profiles were found in mice with circadian rhythm mutations or rats with chronic sleep deprivation (Chandra et al., 2023).

Several probiotics and prebiotics have been shown to have positive effects on sleep, which could be interpreted to mean that sleep health can be managed by supporting the MGBA (Thompson et al., 2020). Additionally, propionate, a known SCFA, may play a role together with other microbial metabolites, while greater levels of propionate have been correlated with promising infant sleep patterns (Heath et al., 2020). These empirical findings support the notion that modulating microbial communities could positively influence sleep regulation and overall health.

Building on these current empirical findings, future hypotheses suggest that the precise manipulation of microbial communities to boost propionate production may improve sleep patterns, decrease Aβ accumulation, and slow cognitive decline. To empirically test this future hypothesis, long-term controlled trials may need to standardize the intervention by assigning either a probiotic or prebiotic regimen aimed at propionate enhancement to subjects with mild cognitive impairment. These impending studies should incorporate objective assessments of sleep quality, utilizing polysomnography, concurrently with measurements of gut-derived metabolites, amyloid/tau biomarkers, and cognitive trajectories over time. By simultaneously targeting gut function and sleep, future research has the potential to uncover synergistic mechanisms and provide a new therapeutic strategy to slow AD progression.

Preclinical studies indicate that microbiota modulation via diet or probiotics can reduce neuroinflammation and amyloid burden, with some animal models showing improved cognitive outcomes. Human trials show that probiotic and dietary interventions may support GBA health, but clinical evidence remains preliminary pending further validation.

### 2.2 Parkinson's disease (PD)

PD patients show exhaustive gut microbiome dysbiosis, such as reduced Prevotella and increased Enterobacteriaceae, which can be related to GI symptoms such as constipation (Hashish and Salama, 2023). It seems that the direction of effect is consistently spread among studies, usually implicating early gut changes occurring prior to the manifestation of motor symptoms (Aho et al., 2019). The changes in the microbial metabolites, immune activation, and gut permeability constituting the pathways identified. Initial studies with probiotics, dietary modifications, and FMT have shown benefits in GI symptoms and, in some instances, motor and non-motor symptoms (Georgescu et al., 2016; Huang et al., 2019; Wang Q. et al., 2021). Controlled studies are ongoing, but present data do favor microbiome modulation as an adjunct therapy. Many consistent alterations in the microbiome lead toward a causative or contributory role, and early interventions of microbiota could influence the progression of the disease and the severity of its symptoms.

#### 2.2.1 Background

**Figure 3:**
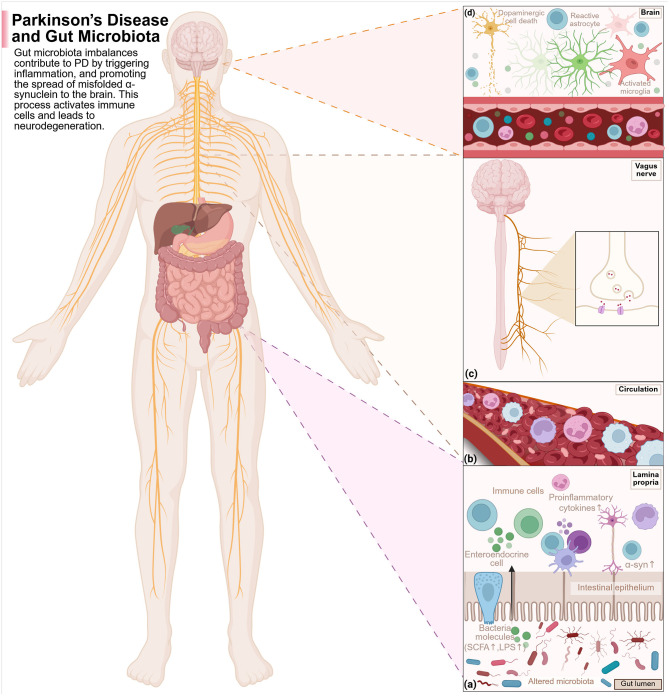
The disruptions of the gut microbiota work together with the immune, endocrine, and nervous systems in PD pathogenesis as follows: **(a)** Disruptions of the gut microbials and their metabolites' composition are able to induce inflammation of the gut. The metabolites can cross the compromised intestinal wall, reach the immune activation of the gut lining, stimulate pro-inflammatory cytokine secretion, and enable α-syn misfolding and aggregation. **(b)** Increased permeability of the gut wall will enable more microbial metabolites and immune signaling molecules to enter into the circulation to induce inflammation in the body. **(c)** The misfolded α-syn of the gut could then enter the brain through the vagus nerve in a cell-cell transmission-like manner that would also be reversible. **(d)** These pathological proteins enter the brain via BBB disruption and vagal channels, activating microglia and astrocytes and leading to neuroinflammation and dopaminergic neuronal degeneration, thus advancing PD. Created with BioRender.com.

PD is the second-most common neurodegenerative disorder after AD. It acts from a lack of equilibrium between two neurotransmitters in the basal ganglia: dopamine (inhibitory) and acetylcholine (excitatory). In typical basal ganglia function, the actions of these neurotransmitters counterbalance each other to refine voluntary movement; however, in PD, the neurons producing dopamine slowly die out, causing an excess of acetylcholine to overstimulate the basal ganglia (Wang Q. et al., 2021). This leads to the major motor symptoms of tremor, bradykinesia, and rigidity, along with postural difficulties, which are classical for PD (Obeso et al., 2017). Affected individuals may show other non-motor symptoms, with some being GI-oriented and emerging years before the classical motor features (Cersosimo et al., 2013). Cognitive manifestations include depression, irritability, and anxiety, while GI manifestations encompass constipation, dysphagia, nausea, and prolonged intestinal transit time (Pfeiffer, 2003; Li et al., 2023). Most non-motor symptoms go unrecognized when this neurodegenerative disease begins in his prodromal form. Diagnosis and treatment usually begin after the disease shows motor symptoms. It has a complicated pathogenesis associated with environmental modifications, genetic predispositions, deregulated dopamine metabolism, neuroinflammation, oxidative stress, and mitochondrial dysfunction (Gökçe Çokal et al., 2017; Wang T. et al., 2020). However, the leading theory behind PD pathogenesis is the accumulation of alpha-synuclein aggregates within cells, which trigger neuroinflammation and neuronal apoptosis (Kim et al., 2019). Gut findings of alpha-synuclein abnormalities in early-stage PD are documented in patients having PD. Imaging studies further corroborate that PD may arise from the gut and subsequently spread into the brain (Horsager et al., 2021, 2022). Through the spinal cord, the brain communicates with all other body systems, including the endocrine system, which regulates body functions such as stress and mood via hormone secretion, and the immune system, which defends the body from pathogenic infection and promotes healing. These systems share signals through the vagus nerve, which carries information from the body's organs to the brain regions (Jackson et al., 2019; Li et al., 2023; Figure 3). Some epidemiological studies carried out in Denmark and Sweden have indicated that people who had undergone complete truncal vagotomies decades ago had a lower risk of developing PD later in life (Svensson et al., 2015; Liu et al., 2017). This points to the possibility of some disturbances in gut microbial position, thereby predisposing to PD.

#### 2.2.2 Vagotomy and insights into the microbiota–gut–brain axis in Parkinson's

The vagus nerve is the tenth cranial nerve. It has been so named from the Latin word meaning “wandering,” because it wanders widely through the body. It originates from either side of the medulla oblongata, exits through the jugular foramen, and descends the neck, chest, and abdomen (Liu and Forsythe, 2021). Along the way, the vagus nerve sends off branches to innervate and regulate almost all the visceral organs. Functionally, it is vital for the control of immune responses via neural signaling. Containing both motor and sensory fibers, the vagus nerve is also the main afferent pathway from the abdominal organs, lungs, liver, stomach, pancreas, and intestines to the brain. Sensory information from the abdominal organs traverses the vagus nerve to the nucleus of the solitary tract in the brainstem, with projections to several other areas of the CNS, including the cerebral cortex and medulla oblongata (Liu and Forsythe, 2021). At present, vagotomy is considered only for those patients who are resistant to pharmacological treatment or who are severely ill with complications, such as perforations or gastric outlet obstruction. In the past, it has also been employed in the treatment of biliary dyskinesia and chronic abdominal pain in neurological conditions (Crile, 1951; Liu and Forsythe, 2021).

In PD, the spread of the pathological alpha-synuclein from the gut to the brain is mediated along the vagus nerve. There are two major forms of vagotomy: truncal vagotomy, which severs all connections, and selective vagotomy, which maintains intestinal innervation (Wang Q. et al., 2021). Studies have noted that vagotomy can inhibit the misfolding of alpha-synuclein in enteric neurons and reduce the appearance of PD symptoms in animal models (Uemura et al., 2018; Kim et al., 2019).

The total vagotomy can be beneficial in reducing the chances of developing secondary PD, according to a cohort study done in Denmark (Svensson et al., 2015). On the other hand, there is a separate study in Sweden that has followed patients after vagotomy and pointed out no relation between vagotomy and PD risk (Liu et al., 2017). However, 5 years of follow-up indicated that within that period, after vagotomy, there is a reduced risk of PD, and very similar results were obtained in the 10-year follow-up. These findings provoke interesting primary questions as to the possible origins of PD-associated alpha-synuclein misfolding from within autonomic nerve fibers of the gut.

Selective or highly selective vagotomy may permit α-synuclein pathology from elsewhere in the GI tract to still reach the vagus nerve and, thereafter, the brainstem. Vagotomy in animal studies is said to disrupt the transfer of α-synuclein derived from the gut to the CNS, but human observational evidence is inconsistent, with some studies indicating no change in PD incidence and others suggesting a reduced risk following truncal vagotomy (Elfil et al., 2020). Currently, there is too immature an understanding of PD pathophysiology to make vagotomy a potential treatment method, and clarification through research is needed regarding the vagotomy-PD relationship. Therefore, no clinical role for vagotomy exists at present.

#### 2.2.3 Duration of disease, motor symptoms, non-motor symptoms, and associated microbiota

The composition of gut microbiota might vary whether PD has an early or late onset. In a study, Pasteurellaceae, Alcaligenaceae, and Fusobacteria were more common in early prodromal PD stages vs. *Comamonas* and *Anaerotruncus*, which were commonly detected in late-onset PD cases (Lin et al., 2018). Some gut pathogens, such as *Aquabacterium, Peptococcus*, and *Sphingomonas*, have been associated with motor complications in PD (Qian et al., 2018).

Weis et al. (2019) examined the impact of PD medications (levodopa and entacapone) on gut microbiota, finding marked changes in *Peptoniphilus, Finegoldia, Faecalibacterium, Fusicatenibacter, Anaerococcus, Bifidobacterium, Enterococcus*, and *Ruminococcus*. For other drugs such as MAO inhibitors, amantadine, and dopamine agonists, no indication was found for an effect on taxa abundance or microbial functions (Horsager et al., 2020). Palacios et al. (2021) reported lower *Clostridium* group IV in PD as well as no strongly associated taxa with levodopa (L-DOPA) use, although preliminary data suggest *Clostridium* cluster IV may be linked to short-term motor-symptom response to L-DOPA, which deserves a thorough controlled validation. Other studies have shown that the other PD drugs acted independently to shape different microbial profiles, which strengthens the rationale for disentangling drug effects from disease signatures (Hashish and Salama, 2023).

Aho et al. (2019) distinguished PD patients who are stable and those with rapid progression, with inconsistent taxa throughout methods and times but uniquely exiting enterotypes, accompanied by a decline in *Prevotella* for the rapid-progression cases. Group differences between PD and controls stayed after the removal of confounds such as deep-brain stimulation (DBS), but differed across studies. In general, replications of the PD-microbiome instances are often poorly developed, presumably as a result of confounding by usage of medication, geographical differences, sequencing methods, and analytical pipelines. Nevertheless, some microbial changes were reported consistently: decrease in Prevotellaceae, *Prevotella*, and *P. copri* (Scheperjans et al., 2015; Unger et al., 2016; Hopfner et al., 2017), and increase in *Akkermansia*/Verrucomicrobiaceae (Keshavarzian et al., 2015; Heintz-Buschart et al., 2018; Lin et al., 2018).

The most important GI symptom is constipation, which impacts almost 60% of people suffering with PD (Pavan et al., 2022). Research indicates that the severity of PD-related constipation helps diagnose the PD stage, with 67% sensitivity and 90% specificity (Hashish and Salama, 2023). References state that the dysbiosis is likely to be responsible for early stage PD GI-related problems, including constipation, with a notable increase in bacterial families that include Lactobacillaceae, Verrucomicrobiaceae, Bradyrhizobiaceae, *Bifidobacterium*, and *Akkermansia* (Baldini et al., 2020; Hashish and Salama, 2023). Among these groups, through greater severity of constipation, longer transit times, and harder stool, *Akkermansia* has been related to constipation (Lubomski et al., 2022b; Hashish and Salama, 2023). Patients suffering from PD and characterized by slowed transit of the intestine may consequently need to receive higher amounts of L-DOPA (Fasano et al., 2013; Weis et al., 2019). Such patients lack in terms of absorption of medicines, thus translating into efficacy problems because of the delays in transit. Constipation may also predispose bacteria to reach excessive populations, particularly *Lactobacillus* species that can execute tyrosine decarboxylation, L-DOPA by converting it into dopamine in the gut hence limiting its release into the bloodstream and causing motor fluctuations (Menozzi and Schapira, 2024). More doses of L-DOPA combined with decarboxylase inhibitors have to be repeated under this scenario, thus denoting a complicated interaction of PD medications with GI tract-related symptoms.

Most people who suffer from PD, may have experienced either the condition of small intestinal bacterial overgrowth (SIBO) or colonization by *Helicobacter pylori* (*H. pylori*; Gabrielli et al., 2011; Felice et al., 2016). SIBO could lead to symptoms like bloating, excessive gas, or impaired absorption of nutrients. A relationship between PD and SIBO was first established in 2021 by the symptom of constipation, indicating that aggravation in the severity of constipation might correlate with an increased risk for SIBO (Dănău et al., 2021). Relief from SIBO not only alleviates the GI discomfort, but it also helps in the amelioration of the motor fluctuations experienced by PD patients (Gabrielli et al., 2011; Fasano et al., 2013). Patients with PD and comorbid SIBO—as opposed to those without it—showed more severe dyskinesia which included prolonged rest time, delayed on-time and off-time. Another proof of this was that motor fluctuations in PD patients improved after SIBO eradication, hence providing more support of the association between SIBO and motor fluctuations (Lubomski et al., 2020). Interestingly, studies show multiple symptoms of PD improve with *H. pylori* antibiotic therapy due to increased absorption and bioavailability of L-DOPA (Pierantozzi et al., 2001). A trial, double-blind, placebo-controlled study pronounced that motor symptoms do improve from hypokinesia over the year since the eradication of *H. pylori* but worsen from flexor rigidity (Dobbs et al., 2010). Although the mechanisms remain unknown, researchers present their theory claiming that the thickening rigidity coexists with SIBO because of overgrowth by certain bacterial strains already present (Felice et al., 2016). The increasing evidence that altered gut microbiota correlate with non-motor features of PD rests on the premise of the need for more evidence to be generated for possible therapeutic strategies to be unveiled.

#### 2.2.4 Interactions between the gut microbiome and device-assisted therapies

Evidence consistently points toward the role of gut microbiota in the persistent interaction upon L-DOPA treatment response. It has been seen that the efficacy of L-DOPA in antibiotic therapy was improved as gut microorganism influences drug metabolism and action (Hashim et al., 2014; Hashish and Salama, 2023; Hey et al., 2023). As one of the most important dopamine-replacement therapies for PD, L-DOPA is commonly given with carbidopa to improve bioavailability and avoid early conversion into dopamine before penetration into the brain (Gandhi and Saadabadi, 2025). Thus, an increasing amount of evidence suggested that the gut microbiome has taken a rather complex interest in influencing the response of the body to L-DOPA treatment. Such studies have shown that antibiotic administration might increase the effectiveness of L-DOPA therapy, indicating that gut microorganisms affect metabolism and the effectiveness of the drug (Hashim et al., 2014). Another important fact is that this L-DOPA is co-administered with carbidopa, another important dopamine-replacement therapy for PD, aiming to increase the bioavailability of the substance and prevent early conversion into dopamine prior to entering the brain (Gandhi and Saadabadi, 2025).

The research by Lubomski et al. (2022b) looked at how the administration of levodopa-carbidopa intestinal gel (LCIG) has effects on the gut microbiome in PD. The LCIG treatment involves a continuous infusion of a carboxymethylcellulose aqueous gel directly into the proximal jejunum via a percutaneous gastrojejunostomy tube, with delivery by a portable infusion pump (Olanow et al., 2014). When assessing alpha diversity (the diversity and evenness of microbial species within a single sample, representative of microbial richness and community balance), they found no marked changes attributable to LCIG. The LCIG application caused changes in the beta diversity (which assesses the differences in microbial community composition between samples, indicating how similar or dissimilar microbial communities are across individuals or time points). Changes in beta diversity reveal that while overall species richness and evenness among the individuals were stable, LCIG can redistribute the abundance of different taxa, thereby altering cohabitation structure and interrelations among microbial species. This pattern holds biological significance in PD in that it suggests that disease- or treatment-related effects may exert selective pressure on the microbial network in the gut without necessarily lowering the overall number of microbial species present, thereby exerting an influence on GBA signaling and L-DOPA metabolism. The acidic condition of LCIG may alter the chemical situation in the gut, which, finally, leads to hypergrowth of *Escherichia/shigella*, being acid-tolerant bacteria (Lubomski et al., 2022b). This observation of microbial shift may contribute to the excessive gut inflammation. Research is scarce on the effect of LCIG on the gut microbiota in PD individuals, and more studies are needed to substantiate findings on gut homeostasis.

Previously reported changes in the abundance of this family Prevotellaceae in advanced PD seem to have been expanded upon by Bedarf and associates, who have shown a dramatic decrease in the numbers of *Prevotella copri* in patients with early-stage PD (Bedarf et al., 2017). Since these species of *Prevotella* produce SCFAs like butyrate, their decrease may affect intestinal barrier function and immune health and thus be involved in the pathogenesis of PD (Hey et al., 2023). Notably, neither MAO inhibitors nor dopamine agonists appear to affect the composition and function of gut microbiota (Bedarf et al., 2017). However, emerging data suggest that different PD therapies exert differential effects on gut microbiota, as shown in Table 4. Since PD patients are frequently on multiple therapies, it will be important for future studies to determine the independent effect of these therapies on the gut microbiome and their potential roles in the progression and management of the disease.

**Table 4:** Overview of studies investigating the effect of DBS and LCIG activation on the composition of the GM.

**References**	**Intervention**	**Sample size**	**Results**
Lubomski et al. (2022b)	4 weeks DBS	PD: 21 DBS: 10 LCIG: 11 HC: 10	Notable differences in alpha and beta diversity relative to LCIG therapy
			Excess *ParaBacteroides* and *Clostridium* may aid post-surgical healing by reducing inflammation
			Elevated *Escherichia/Shigella* levels may be due to LCIG's acidic properties
Lubomski et al. (2022a)	12 months DBS	PD: 74 DBS: 9 LCIG: 10 HC: 74	Increased microbial diversity may result from time-dependent GM changes after DBS and/or LCIG
Melis et al. (2021)	LCIG	PD: 107 LCIG: 38 Naïve group: 23	LCIG therapy increases Enterobacteriaceae, *Escherichia*, and *Serratia* more than levodopa alone
	Higher Enterobacteriaceae levels after LCIG may contribute to gut inflammation

#### 2.2.5 Gut microbiota-based therapeutic interventions

##### 2.2.5.1 Probiotics

The results of human clinical trials (see Table 5) suggest that probiotics may be beneficial as a supportive therapy for PD. *Lactobacillus casei* Shirota is found in milk, which, in one randomized clinical trial, relieved abdominal discomfort while improving stool consistency and bowel movements. The improvement of gut health in patients suffering from PD may be attributable to other probiotic strains, including *Lactobacillus acidophilus* and *Bifidobacterium infantis* (Georgescu et al., 2016). More recently, another randomized clinical trial demonstrated that co-administering Probio-M8 (*Bifidobacterium animalis* subsp. *lactis* Probio-M8) with dopamine agonists improved non-motor symptoms such as cognitive function, bowel regularity, and overall gut health (Sun et al., 2021). Their findings match existing clinical data, supporting the notion that probiotics can influence the MGBA, perhaps as a complementary method of intervention against the advancing PD phenotypes.

**Table 5:** Probiotics may offer supportive therapeutic benefits for Parkinson's disease (PD), as suggested by findings from human clinical trials.

**References**	**Study subjects**	**Probiotic**	**Prebiotic**	**Duration**	**Results**
Cassani et al. (2011)	40 PD patients	*Lactobacillus casei Shirota*	No	5 weeks	Improved stool consistency, and reduction in abdominal pain, while other motor-symptoms were not tested
Georgescu et al. (2016)	40 PD patients	*Lactobacillus acidophilus* and *Bifidobacterium infantis*	No	12 weeks	Improved abdominal pain and bloating, no changes in constipation, while other motor-symptoms were not tested
Borzabadi et al. (2018)	50 PD patients	*Lactobacillus acidophilus, Bifidobacterium bifidum, L. reuteri*, and *L. fermentum*	No	12 weeks	Improved markers of inflammation, while constipation and motor symptoms were not tested
Tamtaji et al. (2019)	60 PD patients	*Lactobacillus acidophilus, Bifidobacterium bifidum, L. reuteri*, and *L. fermentum*	No	12 weeks	Improved motor symptoms, and insulin-related measures, while constipation was not tested
Ibrahim et al. (2020)	48 PD patients	The multi-strain probiotic Hexbio consists of *Lactobacillus acidophilus, L. casei, L. lactis, Bifidobacterium infantis*, and *B. longum*	Yes (FOS)	8 weeks	Improved constipation and gut motility, while no changes found in motor symptoms
Tan et al. (2021)	72 PD patients	*Lactobacillus acidophilus, L. reuteri, L. gasseri, L. rhamnosus, Bifidobacterium bifidum, B. longum, Enterococcus faecalis*, and *E. faecium*	No	4 weeks	Improved constipation, while motor symptoms were not tested
Sun et al. (2021)	82 PD patients	*Bifidobacterium animalis* subsp. *lactis* Probio-M8	No	12 weeks	Improved constipation, motor symptoms, and cognitive functions, and increased serum acetate and dopamine

##### 2.2.5.2 Prebiotics

Prebiotics could be helpful in the management of PD, even if only limited studies have been done on human PD patients to find out how prebiotic supplementation affects them (Table 5). According to a study conducted recently by Liu et al. (2022) it was identified that polymannuronic acid, when given as a prebiotic, might protect dopaminergic neurons from SCFA-mediated anti-inflammatory and anti-apoptotic pathways. A more recent study showed that butyrate levels in PD patients increased due to the production of SCFA after being treated orally with resistant starch, along with improvements in non-motor symptoms (Becker et al., 2022). More human trials are essential for a full understanding of the long-term impact of prebiotics in PD pathologies.

##### 2.2.5.3 FMT

Sampson et al. (2016) were the first to demonstrate that transplanting fecal matter from PD patients into mice genetically engineered to overexpress alpha-synuclein significantly worsened their motor symptoms compared to mice receiving transplants from healthy human donors. In one event where a 71-year-old PD subject was subjected to FMT, improvement in bowel regularity was noted, and tremors disappeared for 2 months after the process (Huang et al., 2019). Reduced striatal dopamine, serotonin levels, and their metabolites were also reported. On the contrary, studies have proven that the fecal microbiota transferred from healthy donors into PD-model mice mitigates their motor impairments and their gut microbiota changes, which were shown by Zhang et al. (2021d) and Zhao et al. (2021).

Various studies have positively linked the use of FMT with a healthy gut microbiome through a decrease in pathogenic microbes like *Desulfovibrio, Akkermansia*, and Proteobacteria and an increase in beneficial groups like Bacteroidetes and Actinobacteria, especially including *Blautia* and *Prevotella* species (Sun et al., 2021). Beyond the gut, FMT provides many improvements in the brain, including reversing cognitive decline, neuroprotective mechanisms by reducing alpha-synuclein accumulation, and restoring striatal levels of dopamine and serotonin (Hashish and Salama, 2023).

Improvement in bowel function and reduction of tremor has been reported (Huang et al., 2019) but such studies are tentative and do not prove efficacy. The use of FMT in routine care of patients with Parkinson's cannot be recommended at this stage.

Animal models show microbiota changes prior to motor symptoms, and probiotic interventions have improved GI and non-motor symptoms in clinical trials. Different PD therapies may influence gut microbiota composition, emphasizing the need to clarify therapy-specific effects.

### 2.3 Amyotrophic lateral sclerosis (ALS)

Recent human studies demonstrate that ALS patients have gut dysbiosis, defined primarily by decreased microbial diversity, such as lower populations of beneficial bacteria like *Faecalibacterium*, and increased levels of potentially harmful species like *E. coli* (Brenner et al., 2018). These changes are associated with increased systemic inflammation markers, and some investigations have made correlations of microbial imbalance with faster disease progression (Obrenovich et al., 2020). However, the evidence for causality remains limited, and the findings across studies have rather conflicting outcomes. Still, gut microbiota profiles may be considered as biomarkers or therapeutic targets.

#### 2.3.1 Background

ALS is a serious and progressive disease that damages both the upper and lower motor neurons, which are the nerve cells that control voluntary muscle movement. It usually begins between the ages of 50 and 70 and is slightly more common in men than women, with a male-to-female ratio of about 1.5–1 (Chiò et al., 2013). Worldwide, ALS affects about 1–2.6 people out of every 100,000 each year, and at any given time, around 4–8 out of every 100,000 people, especially those of Caucasian descent are living with the disease (Al-Chalabi and Hardiman, 2013; Chiò et al., 2013). Roughly 90% of ALS cases happen without a family history (sporadic ALS), while the remaining 10% are inherited (familial ALS). More than 50 genes have been linked to ALS, with the most common being C9ORF72, which causes up to 50% of inherited cases and 5–10% of sporadic ones. Other key genes include SOD1, TARDBP (which codes for TDP-43), and FUS (Renton et al., 2011). Interestingly, ALS and frontotemporal dementia (FTD) are closely related conditions and often share both genetic and disease mechanisms, especially in people with C9ORF72 mutations suggesting they may be part of the same disease spectrum (Parobkova and Matej, 2021). In addition to genetics, certain environmental exposures may increase the risk of developing ALS. These include smoking, military service, pesticide exposure, and heavy metals, with smoking raising the risk by about 40% (Al-Chalabi and Hardiman, 2013).

#### 2.3.2 Clinical presentation and diagnostic challenges

ALS can look very different from person to person in how it begins and how quickly it progresses. In about 70% of people, the first symptoms appear in the limbs usually starting with hand weakness (60%) or leg weakness (40%). Around 30% of patients start with bulbar symptoms, which affect the muscles used for speaking and swallowing, leading to problems like slurred speech (dysarthria), trouble swallowing (dysphagia), and twitching of the tongue muscles (Chiò et al., 2013). A small number (less than 5%) have respiratory-onset ALS, where breathing muscles weaken early, and these cases tend to be more severe.

The disease usually begins in one part of the body and spreads gradually, leading to increasing muscle weakness. On average, there's a delay of 10–16 months between the first symptoms and the official diagnosis (van den Bos et al., 2019). Once diagnosed, median survival is between 2 and 5 years, although patients with bulbar-onset ALS tend to decline more quickly.

ALS is mostly diagnosed based on clinical signs, using the revised El Escorial criteria, which require signs of both upper motor neuron (e.g., spasticity, brisk reflexes) and lower motor neuron damage (e.g., muscle wasting, twitching) in multiple body regions (Brotman et al., 2025). Electromyography (EMG) tests are crucial to show nerve damage and muscle denervation, while nerve conduction studies are used to rule out other diseases. MRI scans are also done to make sure there isn't another condition, such as a structural issue in the brain or spine (Kwan and Vullaganti, 2022). Despite all this, about 10–15% of ALS cases are initially misdiagnosed. Conditions that are often mistaken for ALS include multifocal motor neuropathy, cervical spine disorders, and inclusion body myositis (Kwan and Vullaganti, 2022).

#### 2.3.3 Molecular pathogenesis and disease mechanisms

**Figure 4:**
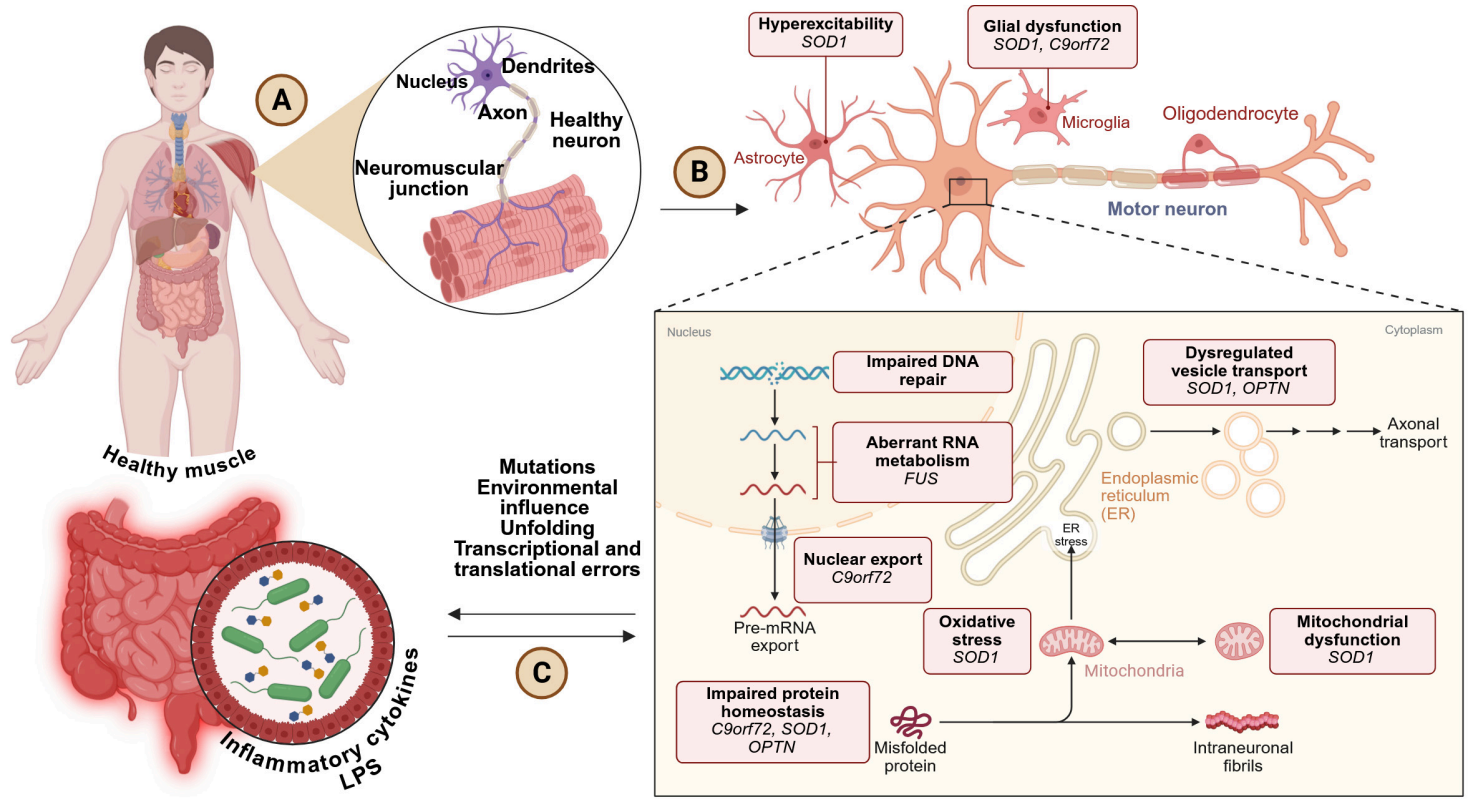
**(A)** ALS starts in the neuromuscular system, composed of normal neurons and muscle fibers. **(B)** Progression of the disease includes neuronal hyperexcitability, glial dysfunction, and neurodegeneration of the lower motor neuron generated by several gene mutations (i.e., SOD1, C9orf72, FUS, OPTN). **(C)** Other contributory causes include defective RNA processing, protein misfolding, mitochondrial dysfunction, ER stress, and inflammation, mostly dependent on environmental and genetic factors, and perhaps gut-derived cytokines. Created with BioRender.com.

The development of ALS is driven by several overlapping biological mechanisms that together cause motor neuron degeneration. One key process is glutamate excitotoxicity, where a buildup of glutamate at the synapse due to reduced function of the transporter EAAT2 (also known as GLT-1) leads to overstimulation of N-methyl-D-aspartate (NMDA) receptors. This causes excessive calcium to enter neurons, damaging mitochondria and triggering cell death (Arnold et al., 2024). Another central feature is the loss of protein balance, especially involving TDP-43, a protein that is abnormally located in the cytoplasm instead of the nucleus in about 97% of ALS cases. In this mislocated state, TDP-43 forms toxic clumps that interfere with RNA processing and disrupt essential cellular functions (Prasad et al., 2019). In patients with mutations in the C9ORF72 gene, the production of harmful dipeptide repeat proteins further disrupts communication between the nucleus and cytoplasm, impairing cell function. Neuroinflammation also plays a major role, with microglial cells releasing inflammatory molecules such as TNF-α, IL-6, and IL-1β, along with ROS, all of which intensify neuronal damage (Liu and Wang, 2017). In addition, astrocytes, which normally support neurons, become dysfunctional through a process called astrogliosis, which worsens glutamate imbalance and reduces neuronal support (Boillée et al., 2006). Other contributors include faulty axonal transport, resulting in buildup of neurofilaments and synaptic breakdown (Taylor et al., 2016), and genetic mutations—such as SOD1 (causing harmful protein accumulation), FUS (interfering with RNA handling), and TBK1 or OPTN (which impair the cell's waste disposal system known as autophagy; Goutman et al., 2022; Figure 4). Together, these processes create a toxic environment that progressively destroys motor neurons in ALS.

#### 2.3.4 The gut microbiome in ALS pathogenesis

Recent studies have shown that the gut microbiome, the community of bacteria living in our digestive system may play an important role in the development and progression of ALS. People with ALS often have an imbalance in their gut bacteria, known as dysbiosis, with fewer helpful bacteria like *Faecalibacterium* prausnitzii and Bifidobacterium, and more potentially harmful ones such as *E. coli* and Dorea species (Brenner et al., 2018). This imbalance may speed up the disease in several ways. For example, reduced levels of SCFAs (like butyrate, which is normally made by good bacteria) can weaken the BBB, making the brain more vulnerable to harmful substances. At the same time, increased levels of inflammatory molecules, such as LPS from harmful bacteria, can lead to systemic-wide inflammation (Obrenovich et al., 2020). The gut microbiome also affects the immune system, with ALS patients showing increased Th17 immune cells and higher levels of IL-17, an inflammatory cytokine that may worsen the disease (Zang et al., 2023). Together, these findings support a strong connection between gut health and brain health in ALS.

#### 2.3.5 Gut microbiota-based therapeutic interventions

##### 2.3.5.1 FMT

There is growing interest in using FMT as a possible treatment for ALS, based on encouraging early research. In one recent case report, an ALS patient showed clinical improvement after receiving FMT, with better scores on the ALS Functional Rating Scale and lower levels of inflammation-related markers like CRP and IL-6 (Yan et al., 2024). It is important to emphasize that evidence from these early reports is limited, and the efficacy and safety of FMT for ALS have not yet been established. Additionally, an ongoing clinical study known as the FETR-ALS trial (a phase II randomized controlled trial) has reported that around 40% of participants experienced a slowing or stabilization of their disease following FMT. Thus far, preliminary clinical observations, mostly from case reports and Phase II studies, seem to indicate that FMT causes changes in gut microbiota composition and inflammatory markers in ALS. However, these are merely early signals, and robust finding regarding efficacy cannot be made until the results of larger randomized controlled trials with determining endpoints come about. Mechanistically, alterations in gut microbial metabolites such as SCFAs could affect the integrity of the BBB, induce systemic endotoxemia, and activate microglia so as to impact disease progression; further studies are required to clarify these pathways. These mechanistic perspectives provide potential explanations for some of the clinical improvements seen and illustrate the various pathways through which FMT might exert its effects on disease processes. Scientists believe that these benefits may be due to several effects of FMT, including the restoration of healthy gut bacteria such as *Faecalibacterium prausnitzii*, improved gut barrier function, and reduced brain inflammation by calming overactive microglial cells in the CNS (Niccolai et al., 2024). Despite these promising early findings, there is insufficient evidence to support the inclusion of FMT in standard ALS treatment.

##### 2.3.5.2 Dietary interventions

Nutrition plays a vital role in the care of ALS patients because the disease causes major metabolic challenges. As ALS progresses, over 80% of patients develop difficulty swallowing (dysphagia), and many also experience increased metabolism, which raises their calorie needs by about 15–20% more normal (Dupuis and Chio, 2023). Losing more than 10% of body weight is linked to a worse outcome, making early and aggressive nutritional support essential. Experts recommend high-calorie diets (around 35–40 kcal per kilogram of body weight per day), often with a high-fat content, which might offer some protective effects on nerve cells (Guillemin et al., 2022). Ketogenic diets which are low in carbs and high in fats have shown potential benefits in small studies, possibly by lowering oxidative stress and improving energy use in cells. However, more research is needed. For patients who can't eat enough due to swallowing or breathing problems, a feeding tube (PEG tube) is usually advised while their lung function is still fairly good (when their forced vital capacity is above 50%). Studies suggest that using a PEG tube at the right time may extend life by 3–6 months (Guillemin et al., 2022). Even with these strategies, it can still be difficult to ensure ALS patients get enough nutrition, especially as their condition worsens and metabolism continues to change.

Preclinical models indicate microbiota manipulation could modulate inflammation, but human data are scarce. Interventions targeting microbiota are a prospective avenue for slowing disease progression.

### 2.4 Multiple sclerosis (MS)

Gut dysbiosis is seen in MS patients as marked by decreased microbial diversity and shifts among specific taxa, like increased *Akkermansia* and reduced SCFA-producers (Jangi et al., 2016). The changes are rather inconsistent but trend toward correlation with disease activity and relapse risk (Tremlett et al., 2016). Regulation on the responses of the immune system is the way through which gut microbiota influence MS. It also modulates the permeability of gut which may facilitate neuroinflammation. The approaches of FMT and probiotics are in their early stages; some case reports and preliminary studies indicate promise in reducing inflammation and relapse rates (Borody et al., 2014; Lavasani et al., 2010). There are clinical trials ongoing to evaluate the efficacy of microbiota-targeted therapies. Evidence supports the role of dysbiosis in MS, particularly on immune regulatory pathways; interventions to restore microbiota are promising but require more definitive trials.

#### 2.4.1 Background

MS is an autoimmune disease of the CNS, whose hallmark is the demyelination and consequent damage to the demyelinated axons. Demyelination causes a multitude of neurological symptoms, including loss of coordination of movement, ataxia, visual impairments, psychiatric abnormalities such as depression, and cognitive decline (Preiningerova et al., 2022). MS likely results from a combination of genetic and environmental factors (Preiningerova et al., 2022). It is normally diagnosed based on clinical symptomatology complemented by MRI with lesions of various stages, combined with biomarkers of immunologic activity in CSF, namely oligoclonal bands and elevated IgG index (Thompson et al., 2018). The experimental autoimmune encephalomyelitis (EAE) animal model of MS has proven the pathogenic function of myelin-reactive T cells in MS pathogenesis (Laaker et al., 2021). The triggers of activating self-reactive clones are still to be determined, but may be common infections, molecular mimicry, or inflammation signals from gut microbiota (Westall, 2006). Histological evidence has shown that the activated T cells cross the BBB, invade the CNS, and congregate around small venules to trigger localized inflammation (Kuhlmann et al., 2017). Over the decades, the EAE model, focused primarily on T cells, has been the model for MS pathogenesis and treatment studies (Preiningerova et al., 2022). Current treatments are largely the primary application of disease-modifying treatments, immunosuppressants, immunomodulators, and immune-reconstitution therapies, in addition to symptomatic treatments such as anticholinergics for urinary incontinence and pain medication for neuropathic pain (Dobson and Giovannoni, 2019). Patient-dependent conditions affecting the effectiveness of treatment or, quite possibly, affecting outcomes through the gut microbiota, however, are still to be established.

#### 2.4.2 Dysbiosis of gut microbiota in MS

**Figure 5:**
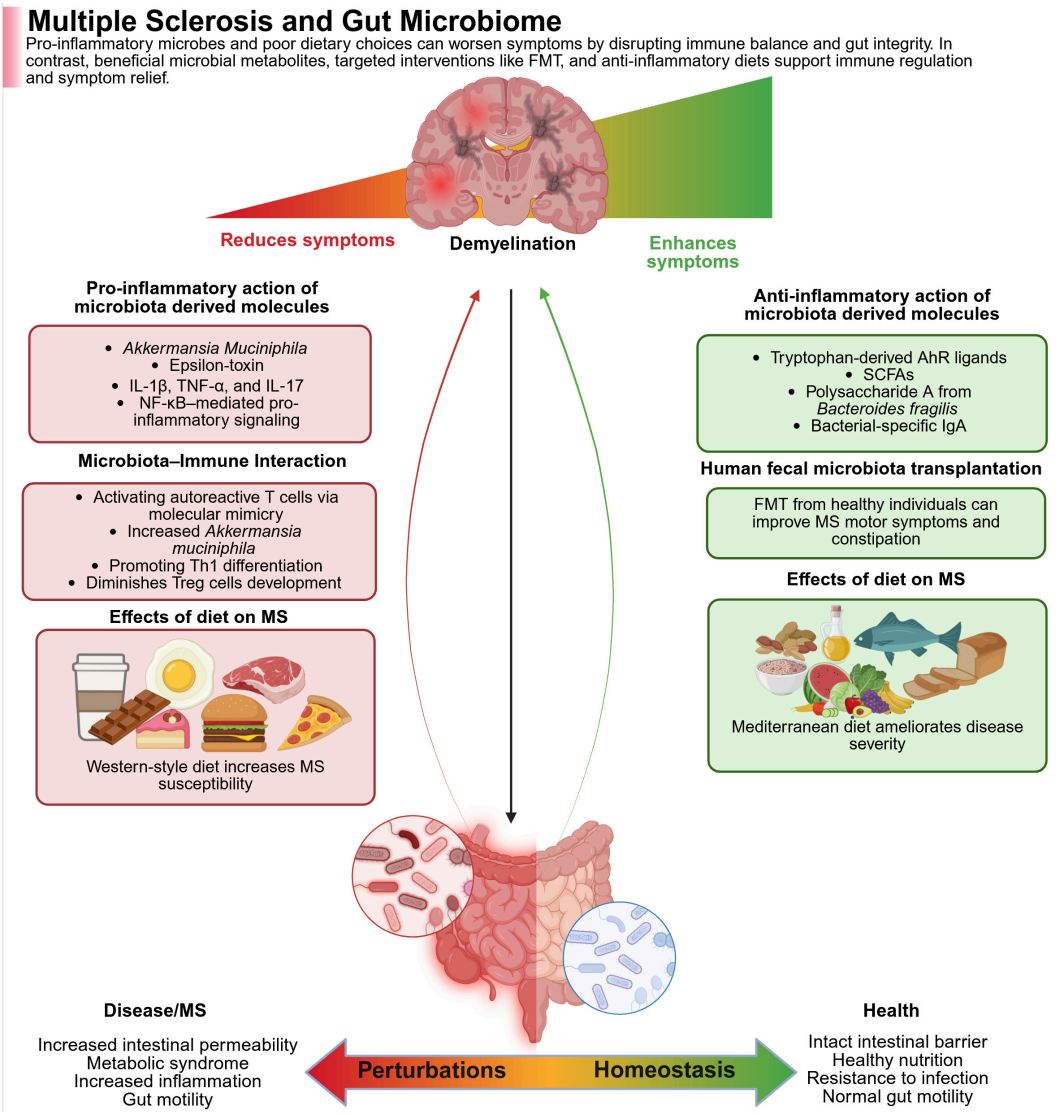
The figure shows how gut microbes and diet impact the course of multiple sclerosis (MS). Whereas, pathogenic microbes and a Western diet trigger gut inflammation, both of which are detrimental in MS, while beneficial microbes, anti-inflammatory metabolites, and a MeDi are all gut-healthy and alleviate symptoms. In the left panel (Red—Worse MS), the pro-inflammatory microbes like *Akkermansia muciniphila* and their toxins activate immune pathways (e.g., NF-κB), trigger autoimmune responses, and damage gut integrity. Poor diet further increases inflammation and disease risk. In the right panel (Green—alleviation of MS), the beneficial metabolites (e.g., SCFAs, tryptophan ligands), as well as polysaccharides from *Bacteroides fragilis*, activate the immune system. Fecal transplants combined with healthy diets reduce inflammation, improve gut barrier function, and ease MS symptoms. Created with BioRender.com.

In patients with MS, it was found that the composition of their microbiota was relatively reduced from that of healthy individuals. Studies are not entirely consistent, but they mostly report an increase in Akkermansiaceae and Methanobacteriaceae, and a decrease in SCFA-producing Bacteroidetes and Clostridia clusters (Atarashi et al., 2011; Palm et al., 2015; Wang et al., 2019). While EAE models have offered valuable insight into adaptive immune responses, demyelination, and axonal damage in MS, they fall short in replicating the full spectrum of human MS pathology, particularly the disease's onset and heterogeneity (Figure 5).

GI symptoms, including anorectal dysfunction such as constipation and fecal incontinence, are commonly reported in MS, affecting approximately 40% of patients (Nusrat et al., 2012). Additionally, Minuk and Lewkonia (1986) have even reported familial clustering of Inflammatory Bowel Disease (IBD) and MS, suggesting that they have genetic or environmental risk factors in common. In the past few years, several studies have explored the differences in fecal gut microbiota between MS patients and healthy controls, revealing that gut microbial dysbiosis with both depletion and enrichment of certain gut microbiota in MS patients, as shown in Table 6. For example, Chen et al. (2016) reported increased levels of *Pseudomonas, Mycoplasma, Haemophilus, Blautia*, and *Dorea*, and reductions in *ParaBacteroides, Adlercreutzia*, and *Prevotella* in RRMS patients. Whether these changes are a cause or a consequence of the disease remains unclear. Increased levels of *Methanobrevibacter* and *Akkermansia* are increased, whereas those of *Butyricimonas* decreased in RRMS patients (Jangi et al., 2016).

**Table 6:** Alterations in gut microbiota and associated functions in MS patients.

**Sample size**	**Sample sources**	**Changes of gut microbiota**	**Associated functions and pathways**	**References**
MS group: *n* = 31; HC group: *n* = 36	Stool samples from RRMS patients (active/remission) vs. HCs	*↑Pseudomonas, ↑Pedobacter, ↑Blautia, ↑Dorea, ↑Mycoplana, ↓Adlercreutzia, ↓Collinsella, ↓Lactobacillus, ↓ParaBacteroides*	Signal transduction Lipid transport and metabolism Immune defense mechanisms Fatty acid biosynthesis Membrane transport systems	Chen et al., 2016
MS group: *n* = 60; HC group: *n* = 43	Stool samples from RRMS patients (remission) vs. HCs	*↑Methanobrevibacter, ↑Akkermansia, ↓Butyricimonas*	Inflammatory cells recruitment Barrier function disruption	Jangi et al., 2016
MS group: *n* = 7; HC group: *n* = 8	Stool samples from RRMS patients (remission) vs. HCs	*↑Akkermansia, ↑Faecalibacterium, ↑Coprococcus, ↓*Moraxellaceae	Not addressed	Cantarel et al., 2015
MS group: *n* = 20; HC group: *n* = 40	Stool samples from RRMS patients (remission) vs. HCs	*↑Bifidobacterium, ↑Streptococcus, ↓Bacteroides, ↓Faecalibacterium, ↓Prevotella, ↓Anaerostipes*	Systemic inflammation	Miyake et al., 2015
MS group: *n* = 19; HC group: *n* = 17	Small intestinal mucosa from active RRMS vs. HCs and inactive MS	↑Firmicutes*/*Bacteroidetes *ratio, ↑Streptococcus, ↓Prevotella*	Differentiation and an increase in the Th17 cell population	Cosorich et al., 2017
MS group: *n* = 71; HC group: *n* = 71	Stool samples from RRMS patients (remission) vs. HCs	*↑Acinetobacter, ↑Akkermansia, ↓ParaBacteroides*	The differentiation of Treg cells and Th1 cells	Cekanaviciute et al., 2017

↑, Increase; ↓, Decrease.

In addition, differences in corresponding changes in expression levels of genes associated with dendritic cell maturation, interferon signaling, and NF-κB signaling pathways in circulating T cells and monocytes have been noted (Jangi et al., 2016). The relation between the variation in gut microbiota diversity and relapse risk in children with MS was found with testing by Tremlett et al. (2016); thus, the depletion of *Fusobacteria* correlated with the relapse risk of pediatric MS. Nevertheless, all those discoveries were performed on gut microbiota obtained from stool samples of MS patients in remission. A recent study has been done on changes in microbiota within small intestinal tissues from MS patients in the active phase. The authors reported an increased Firmicutes/Bacteroidetes ratio and *Streptococcus* abundance, with a reduction in *Prevotella* strains in patients with active MS when compared with healthy controls and patients with MS in remission (Cosorich et al., 2017). Moreover, the relative presence of *Prevotella* strains was inversely associated with Th17 cells in the small intestine, while positively related to disease activity (Cosorich et al., 2017). Although changes in gut microbiota have been reported, it remains uncertain whether these changes are a cause or a consequence of the disease.

#### 2.4.3 Treatment strategies

##### 2.4.3.1 Probiotics

Synergistic therapeutic effects may be exerted by the different strains in probiotic cocktails; therefore, three strains of *Lactobacillus*, each shown alone to have a protective effect against the development of EAE when administered before disease onset, were shown to inhibit established disease when administered as a therapeutic mixture. In MS patients, administering a mixture of probiotics (enriched with *Lactobacillus, Streptococcus*, and *Bifidobacterium*) switched the peripheral immune response to an anti-inflammatory one and reversed the microbiota composition changes associated with MS (Lavasani et al., 2010). In this short-term study, it was not assessed whether these changes were associated with clinical improvement; however, data from randomized double-blind placebo-controlled clinical trials lasting 3–4 months with a similar probiotic mixture (*Lactobacilli* and *Bifidobacteria*) suggest that a daily probiotic may improve clinical symptoms in MS (Tankou et al., 2018b).

Despite some studies suggesting a positive impact of probiotics, recent meta-analyses on EAE have been quite disappointing (Valizadeh et al., 2021). One of the outcomes observed from meta-analysis prescribed administration of probiotics to be associated with a considerable decline in risk of mortality, although this observation holds only for female animals. Furthermore, the meta-analysis confirmed promising effects of probiotics on the prevention as well as management of EAE (lower incidence, delayed expression of symptoms, and less severe symptoms). Using *Enterococci* bacteria rendered the most hopeful results. Hence, the authors are concluding that it's worth it to conduct trials in humans (Valizadeh et al., 2021). A recent meta-analysis in relapsing-remitting MS patients stated four trials that included 213 patients (106 under intervention) and summarized that there was improvement in disability and depression, as well as general health in patients to whom probiotics were administered (Mirashrafi et al., 2021). Such results should be interpreted with caution. Another study of nine MS patients reveals some correlation with microbiome composition changes and an inflammatory cytokine shift in the blood during the weeks of treatment via probiotics (Tankou et al., 2018a).

##### 2.4.3.2 Antibiotics

According to observations, a combination of broad-spectrum antibiotics inhibited the development of EAE and altered the clinical course during the progressive phase of EAE (Ochoa-Repáraz et al., 2009; Colpitts et al., 2017). Human trials showed that in people with high-risk features, treatment with minocycline reduced in 6 months the risk of conversion to MS, decreased lesion volume, and showed the absence of new enhancing lesions, but this effect did not last beyond 24 months of study (Preiningerova et al., 2022). In SJL mice with EAE, a 7-day oral antibiotic regimen (ampicillin, vancomycin, neomycin, metronidazole) before disease induction led to amelioration of the disease through an accumulation of Tregs in the peripheral lymph nodes (Ochoa-Repáraz et al., 2009). Antibiotic mixture administered in NOD/ShiLt mice pre-EAE improved the disease course, correlated with enhanced Tregs and altered gut microbiota in Peyer's patches (Colpitts et al., 2017). Following this pattern, TMEV-infected SJL/J mice treated orally with this same antibiotic mixture showed protection against motor dysfunction, axonal damage, and CNS immune infiltration, likely through enhanced CD4^+^CD39^+^ T cells, CD5^+^CD1d^+^ B cells, and downregulated IL-17 in the periphery (Mestre et al., 2019).

##### 2.4.3.3 FMT

The striking effects of FMT in MS patients have always found their way into case reports in scientific literature. One fortunate report is that of 3 patients diagnosed with MS who were dependent on wheelchairs yet improved neurologically after FMT for constipation to the point that they could walk without assistance (Borody et al., 2014; Vendrik et al., 2020). Rebuilding the gut microbiota represents an exciting new approach to the management of MS, but it will need well-constructed controlled studies to be scientifically validated. FMT in animal models has been found to reduce the abundance of the *Akkermansia* genus (in phylum Verrucomicrobia) and increase the abundance of the *Prevotella* genus (in phylum Bacteroidetes) in gut microbiota (Tankou et al., 2018b), which is in line with findings of decreased gut *Akkermansia* after probiotic interventions and increased gut *Prevotella* after first-line disease-modifying treatments and time-restricted eating in MS patients (Jangi et al., 2016; Barati et al., 2023). An in-depth investigation including metagenomics in a single MS patient following FMT not only showed altered composition of gut microbiome with a highly sustained production of SCFAs, improved gait, and no relapse during a year of follow up, but also showed a sustained increase in serum levels of BDNF known to be low in MS (Engen et al., 2020).

A recent proof-of-concept single-subject longitudinal study investigating the putative impact of FMT on relapsing-remitting MS was conducted. The patient underwent FMT infusion with material from five healthy donors and was followed for 12 months for clinical assessments, detailed descriptions of fecal microbiome composition, fecal SCFA concentration measures, and serum levels of inflammatory and neuroprotective biomarkers (Engen et al., 2020). The treatment given to this patient resulted in an improved microbiome with an increase in bacterial diversity, partly due to an increase in the relative abundance of butyrate-producing bacterial species, which were paralleled by an increase in butyrate concentration, an anti-inflammatory SCFA (Engen et al., 2020). The microbiota-altered state correlated with lower levels of inflammatory cytokines associated with increased serum levels of the neuroprotective factor, brain-derived growth factor. The patient improved clinically in gait, and throughout the study, improvements were seen in walking and balancing metrics. However, it is important to note that these findings are preliminary, and the evidence supporting FMT as a treatment for MS remains limited. In like fashion, a case report suggested that FMT treatment of a patient with secondary progressive MS for *Clostridium difficile* enterocolitis correlated with disease stabilization (Makkawi et al., 2018). More rigorous, controlled trials are needed to confirm the potential benefits and safety of FMT as a strategy for MS. These observations call for renewed clinical efforts to investigate the merits of restoring the microbiota through FMT as a complementary strategy to MS treatment, and clinical trials are ongoing.

While detailed human studies are limited, MS involves gut dysbiosis that influences immune regulation, with preclinical data suggesting certain bacterial taxa may modulate neuroinflammatory responses. Microbiome-targeted interventions like probiotics and diet have shown potential to modulate immune activity and may reduce disease activity, though more robust clinical evidence is needed.

## 3 Mood and anxiety disorders

### 3.1 Major depressive disorder (MDD)

The microbiota of patients suffering from MDD represents much less diversity and altered abundances of taxonomies involved in neurotransmitter synthesis and immune modulation, both mainly consistent in terms of pointing toward dysbiosis (Barandouzi et al., 2020; Valles-Colomer et al., 2019). The GBA effects its influence on depression through neuroinflammatory pathways alteration of neurotransmitter precursors and the secondary dysregulation of HPA axis. Probiotics have been used in connection with dietary interventions leading to improvements in mood and inflammation in human trials (Kazemi et al., 2019). Even though the application of FMT remains experimental, some studies show small benefits toward lowering depressive symptoms (Green et al., 2023). Studies continue to accumulate evidence that correlate microbiome alterations with depression, promising microbiota-based approaches pending further validation.

#### 3.1.1 Background

MDD is characterized by constant depressed mood, anhedonia, altered sleep and appetite, fatigue, guilt, hopelessness, and suicidal tendencies (Arnaud et al., 2022). Over the years, a rising trend has been seen in the prevalence of depression since the time of observation (Cui et al., 2024). In 2018, MDD disease burden ranked third in the world according to the WHO, and by 2030, it is supposed to rise to the first (Malhi and Mann, 2018). A psychiatric disorder is formed due to the complex interplay of environmental factors and genetic vulnerability (Souza et al., 2023). The risks of developing several physical comorbidities like cardiovascular diseases, stroke, diabetes, and obesity along with social stigma are quite higher for MDD persons (Penninx et al., 2013). Some of these revolve around education, work, and relationships (Campbell et al., 2022). In case antidepressants are used in conjunction with psychotherapy, some patients can get better. Unfortunately, not all respond to treatment. As many as 30–50% of depressed patients respond only partially to antidepressants, leaving them with substantial residual symptoms (Davis et al., 2021). Additionally, 10–30% of patients of those who are resistant to the effects of antidepressants and thus non-responders to treatment. Differences in treatment response may be indicative of differences in etiologies and mechanisms, like neuroinflammation, changes in the neuroendocrine system, or alterations in the gut microbiome (Mousten et al., 2022). This greatly simplifies a complex process, but a map of the human microbiome may offer new opportunities for tracking therapeutic responses in depression.

Another study showed that both psychosocial as well as physical stressors influence the body's response through the HPA axis (Godoy et al., 2018). The HPA axis initiates a response in times of stress, stopping the activity through negative feedback, engaging the hippocampus and the paraventricular nucleus of the hypothalamus. A properly perceived stress situation calls for a rapid and vigorous response, along with a timely cessation of that response (McEwen and Akil, 2020). Dysfunction of the HPA axis is common in individuals with MDD and is capable of activating the axis that would alter gut microbiota and increase gut permeability (De Punder and Pruimboom, 2015).

MDD is also marked by systemic inflammation with high cytokines IL-6, TNF-α, and IL-1β (Troubat et al., 2021). The increased level of inflammatory cytokines is perhaps a result of dysbiosis of the gut microbiota, which may further weaken the integrity of the gut barrier. These stimuli will also stimulate brain microglia and trigger the NLRP3 inflammasome to release IL-1β and IL-18 (Troubat et al., 2021). The released mediators would also support neuroinflammation. Inflammation also shifts tryptophan metabolism away from serotonin to neurotoxic kynurenine pathway metabolites, like quinolinic acid, and disturbs neurotransmission and thus plays a role in depressive symptoms (Chen et al., 2021; Liang et al., 2024). Major depression is treated by various pharmacological and non-pharmacological interventions. Exercise and lifestyle modification exert substantial antidepressant effects, especially for mild MDD (Marx et al., 2023). Therefore, modulation of gut microbiota mechanisms might be the key to symptom relief of MDD.

#### 3.1.2 Microbiota dysbiosis and depressive behaviors

*Escherichia* and *Enterococcus* produce serotonin, while *Bifidobacterium* and *Lactobacillus* synthesize GABA (Socała et al., 2021). Microbes like *Coprococcus* and *Faecalibacterium* also produce acetate, butyrate, and propionate, which are known to affect the immune, endocrine, and nervous systems (Valles-Colomer et al., 2019). Gut SCFAs promote serotonin synthesis in the gut and further communicate with the brain via the vagus nerve. Although SCFAs can cross the BBB, serotonin and GABA generally do not, unless barrier integrity is compromised, as seen in stress and depression models (Margolis et al., 2021). A translation from gut microbiota research to clinical settings is undermined by the complexity surrounding individual microbial profiles (Galland, 2014). Investigative efforts so far concerning enterotypes, such as alpha diversity, the Firmicutes-to-Bacteroidetes ratio, aim at the identification of an MDD-specific fecal signature (Valles-Colomer et al., 2019). Reduced Firmicutes proportion in MDD patients, as a suggestion for the establishment of an MDD biomarker, was reported by Jiang et al. (2015). This, however, was followed by systematic reviews showing inconsistent results and hence raised questions on replicability (Barandouzi et al., 2020). Most studies have shown that the GM of MDD patients differs from that of controls at the phylum, class, order, family, and genus levels, but with a particular emphasis on differences at the phylum, family, and genus levels (Table 7).

**Table 7:** Alterations in the gut microbiota abundance in patients with MDD.

**Sample**	**Age**	**Phylum**	**Family**	**Genus**	**References**
MDD (*N* = 63) HC (*N* = 30)	MDD: 28 years, HC: 29 years	Actinobacteria↑	Bifidobacteriaceae↑, Lactobacillaceae↓	*Agathobacter↑, Bifidobacterium↑, Blautia↑*	Dong et al., 2022
MDD (*N* = 43) HC (*N* = 47)	MDD: 21 years, HC: 22 years	Bacteroidetes↑, Firmicutes↓	Ruminococcaceae↓	*Flavonifractor↑, Ruminococcus↓, Faecalibacterium↓*	Liu R. T. et al., 2020
MDD (*N* = 62) HC (*N* = 46)	MDD: 39 years, HC: 36 years	Bacteroidetes↑, Proteobaeteria↑, Fusobacteria↑, Firmicutes↓, Actinobacteria↓	Rikenellaceae↑, Porphyromonadaceae↑, Oscillospiraceae↑, Corynebacteriaceae↑, Ruminococcaceae↓, Lachnospiraceae↓, Eubacteriaceae↓, Lactobacillaceae↓	*Eggerthella↑, Streptococcus↑, Oscillibacter↑, Bacteroides↓*	Chen et al., 2021
MDD (*N* = 36) HC (*N* = 37)	MDD: 45 years, HC: 41 years	Actinobacteria↑, Firmicutes↑, Bacteroidetes↓	Bifidobacteriaceae↑, Lachnospiraceae↑, Prevotellaceae↓	*Bifidobacterium↑, Blautia↑, Eggerthella↑, ParaBacteroides↑, Streptococcus↑, Prevotella↓*	Chung et al., 2019
MDD (*N* = 27) HC (N =27)	MDD: 48 years, HC: 42 years	Firmicutes↓	Lachnospiraceae↓, Ruminococcaceae↓	*Prevotella↓, Faecalibacterium↓*	Huang et al., 2018
MDD (*N* = 37) HC (*N* = 18)	MDD: 42 years, HC 46 years	Bacteroidetes↓	Lachnospiraceae↓	*Alistipes↑, Oscillibactergenus*↑	Naseribafrouei et al., 2014

↑, Increase; ↓, Decrease.

Higher relative abundance of proinflammatory bacteria like *Eggerthella, Atopobium* was found to be increased, and decreased relative abundance of *Faecalibacterium* in the subjects with MDD (Knudsen et al., 2021). Similarly, patients with MDD also had decreased relative abundance of the genera *Coprococcus* and *Faecalibacterium* compared to non-depressed individuals in a meta-analysis by Sanada et al. (2020). The meta-analytical study shows decreased abundance in the MDD patients when compared to controls in the bacterial families Veillonellaceae, Prevotellaceae, and Sutterellaceae, genera *Coprococcus, Faecalibacterium, Ruminococcus, Bifidobacterium, Escherichia*, and shows an increase in abundance in Actinomycetaceae family and *Paraprevotella* genus (Sanada et al., 2020). Interestingly, *Faecalibacterium* served as a primary butyrate-producing bacterium in the gut, being crucial for gut homeostasis and possibly alleviating depressive symptoms at least in animal models (Stilling et al., 2016).

#### 3.1.3 Gut microbiota-based therapeutic interventions

##### 3.1.3.1 Probiotics

A double-blind study showed the results of *Lactobacillus rhamnosus* HN001 from mid-pregnancy to 6 months postpartum, which decreased the signs of postpartum depression and anxiety symptoms (Slykerman et al., 2017). They saw improved psycho-emotional scoring in patients receiving a probiotic as compared to those on placebo. However, clinically significant anxiety seemed to have been reduced; the findings, however, were not statistically conclusive for the effects of this clinical trial on the incidence of postpartum depression (Slykerman et al., 2017). In an 8-week open-label trial, a group of researchers evaluated the effect of *Clostridium butyricum* MIYAIRI 588 (CBM588) in patients with treatment-resistant MMD in an open-label study (Miyaoka et al., 2018). Improvements were seen in clinician—and self-rate measures of depression and anxiety. It was well-tolerated, with only mild and short-lived effects being reported. The findings imply that there could be possible therapeutic benefits from CBM588, although confirmation is needed from larger, placebo-controlled trials (Miyaoka et al., 2018).

According to Tian et al. (2019b), increased BDNF levels, greater populations of butyrate-producing bacteria, and modulation of the HPA-axis were noticed in mice administered the *Bifidobacterium longum* subspecies *infantis* strain CCFM687 and developing depressive-like behavior as a consequence of stress. In fact, treatment with the bacterium *Akkermansia muciniphila* diminished the inducement of a depression-like behavior caused by chronic stress, where the regulation of this metabolic profile stands on such variables as acute corticosterone, dopamine, and BDNF (Ding et al., 2021). During another clinical trial, a probiotic/magnesium spirulina complex with *Lactobacillus acidophilus, Bifidobacterium bifidum*, and *Streptococcus thermophilus* was used with an adjunctive effect on current SSRIs for drug-resistant MDD patients. Overall, remarkable improvements in depressive symptoms and quality of life were observed; unfortunately, when the supplemental probiotics were taken away, relapsed into depression followed (Bambling et al., 2017). Those findings suggest that probiotics also may provide a further powerful adjunctive agent in improving resistance to antidepressant drugs and in averting the recurrence of depression, besides improving depressive symptoms through multiple means.

##### 3.1.3.2 Prebiotics

Among the long-term supplementation with prebiotics, FOS, GOS, or their combination, promising results appear in demonstrating significant antidepressant and anxiolytic activities in mice (Burokas et al., 2017). Indeed, these behavioral improvements are clear indicators of gut microbiota modulation in which *Akkermansia* may increase along with beneficial SCFAs such as acetate and propionate, and the decrease of isobutyrate and stable n-butyrate levels in the gut. The most prominent effects of this combination, FOS+GOS, include diminished baseline and stress-induced corticosterone and a decrease in splenic IL-6 and TNF-α levels under chronic stress (Burokas et al., 2017). Evidencing changes indicate the possibility of prebiotics having neuroprotective, stress-buffering effects through the modulation of the MGBA by restoring microbial homeostasis, changing neuroendocrine responses, or affecting gene expression in stress-related brain regions (Burokas et al., 2017).

##### 3.1.3.3 FMT

According to Doll et al. (2022), the clinical trial with oral administration of FMT capsules in depressed irritable bowel syndrome (IBS) patients enhanced bacterial alpha diversity and increased the abundance of bacterial communities predominantly *Bacteroides immitis* and *Bacteroides thicketi*, as well as an attestation to significant improvement in depressive symptoms.

A pilot 8-week double-blind trial delved into the feasibility and safety of FMT in adults with moderate to severe MDD (Green et al., 2023). Participants were assigned to active or placebo groups receiving enemas prepared with donor stool and saline. No serious adverse events were reported, and mild-to-moderate effects occurred similarly across groups. An effective blinding procedure was utilized. Initial outcomes improve GI symptoms along with possible improvements in quality of life (Green et al., 2023). These findings point toward the possibility of providing FMT as an acceptable intervention requiring further investigation in larger controlled trials. However, FMT's mechanism of action in psychiatric disorders remains uncertain, donor screening and long-term safety require further clarification, and at present, no recommendations can be made for its routine use outside of clinical research.

Animal studies show that probiotics can reduce depressive-like behaviors through anti-inflammatory pathways. Human trials with psychobiotics demonstrate improvements in mood and stress markers, supporting microbiota modulation as a promising adjunct therapy.

### 3.2 Bipolar disorder (BD)

Research has shown that people suffering from BD have an altered gut microbiome with much reduced amounts of useful organisms, such as *Faecalibacterium*, and increased amounts of potentially harmful taxa, such as Actinobacteria, which is featured as one of the alterations (Painold et al., 2019). More relevant, microbiota profiles are related to the illness phase, severity of the condition, and with some inflammatory markers such as IL-6; this means that the changes in microbiota shifts may affect neuroinflammatory pathways that are involved in mood regulation. While these associations are robust, interventional evidence remains preliminary, mainly from small-scale probiotic or dietary modification studies.

#### 3.2.1 Background

BD is a chronic psychiatric condition characterized by alternating episodes of depression and elevated mood states, which may manifest as mania or hypomania. These episodes often alternate with periods of euthymia but can also occur in rapid succession or with mixed features. The disorder is classified into two main subtypes: bipolar I disorder (BP-I), defined by the presence of at least one full manic episode, and bipolar II disorder (BP-II), characterized by hypomanic episodes without progression to full mania (Grande et al., 2016). Longitudinal studies highlight the persistent and fluctuating nature of BD. Research indicates that individuals with BP-I remain symptomatic for nearly half of their illness course, with depressive symptoms being the most frequent and persistent, significantly outweighing manic/hypomanic and mixed episodes (Nierenberg et al., 2023). Subsyndromal symptoms are more prevalent than full-threshold episodes, while psychotic features occur infrequently and primarily during manic states. The illness trajectory is highly variable, marked by frequent shifts in symptom polarity, underscoring the chronic and recurrent nature of BD (Grande et al., 2016). Epidemiological data further demonstrate that over 90% of individuals with BD experience recurrent episodes, which contribute to progressive neurobiological changes, cognitive decline, and increased medical and psychiatric comorbidities (Nierenberg et al., 2023). Manic episodes are defined by at least 1 week of persistently elevated, expansive, or irritable mood accompanied by increased activity or energy, whereas hypomanic episodes present similar features without marked functional impairment or hospitalization. The presence of psychotic features necessitates classification as mania. The global burden of BD is substantial. The World Mental Health (WMH) survey, using the WHO Composite International Diagnostic Interview (CIDI 3.0), identified BD as one of the most disabling psychiatric conditions, with affected individuals averaging 41.2 days of role impairment per year 36.5 of which were directly attributed to BD (Alonso et al., 2011). These findings emphasize the critical need for early intervention, personalized treatment strategies, and ongoing research into the disorder's pathophysiology and long-term management (Grande et al., 2016; Nierenberg et al., 2023).

#### 3.2.2 Gut microbiota alterations in BD

Biologically, people with BD, especially those who haven't started treatment, show noticeable changes in their gut microbiome. A study by Hu et al. (2019) found that these untreated patients had significantly less diverse gut bacteria (lower α-diversity) compared to healthy individuals. They also had unique bacterial compositions (Hu et al., 2019). These changes were found not only in untreated individuals but also in those who were being treated with the medication quetiapine. The study also looked closely at quetiapine, which was used to treat depressive episodes in BD. It was given on its own (monotherapy) at a dose of 200–300 mg daily for 4 weeks. All participants were confirmed to be experiencing a depressive episode using structured psychiatric interviews and rating scales like the HDRS-17 and MADRS. After treatment, there was a clear improvement in symptoms. Although quetiapine is not directly labeled as an antipsychotic in this study, the effects it had both metabolically and on gut bacteria are consistent with what is known about that class of drugs. Importantly, the study only explored quetiapine's use in bipolar depression and did not extend its findings to other mental health conditions (Hu et al., 2019).

**Figure 6:**
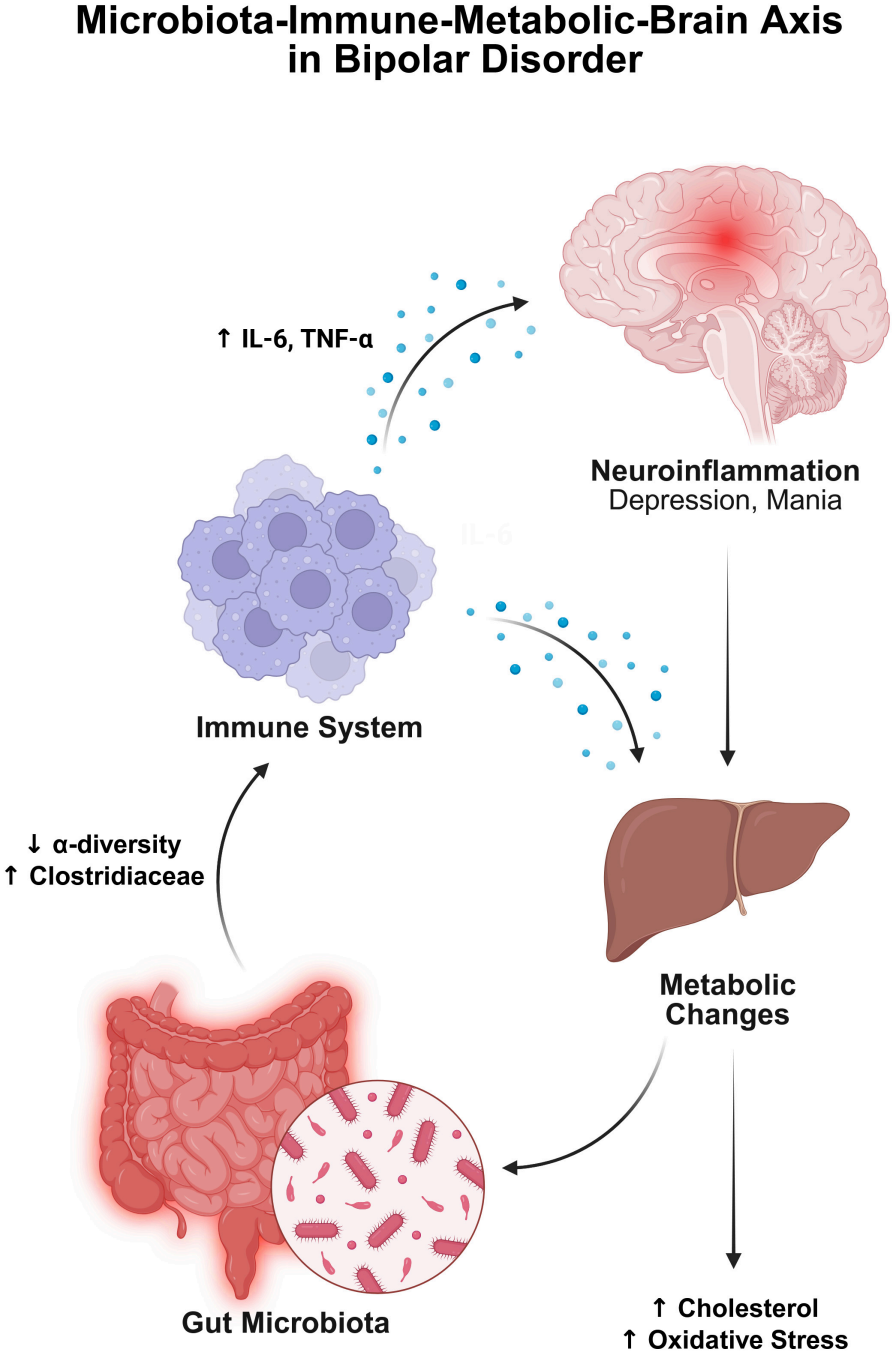
This schematic illustrates the microbiota–immune–metabolic–brain axis in BD. Gut dysbiosis, characterized by reduced microbial α-diversity and increased Clostridiaceae, is associated with immune activation. Elevated pro-inflammatory cytokines, particularly IL-6 and TNF-α, contribute to neuroinflammation, which is implicated in mood symptoms such as depression and mania. IL-6 also drives metabolic disturbances, including increased cholesterol and oxidative stress. These metabolic changes may, in turn, influence gut microbial composition, suggesting a bidirectional feedback loop. Together, these pathways highlight the integrated role of the gut–immune–metabolic axis in BD pathophysiology. Created with BioRender.com.

On the genetic side, research by Heun and Maier (1993) showed that BP-II may be genetically different from BP-I and unipolar depression. In their family-based study, relatives of people with BP-II had a higher risk of also having BP-II, suggesting it tends to run specifically in families (Heun and Maier, 1993). In contrast, less severe mood disorders did not show this familial pattern, supporting the idea that BP-II is a distinct and genetically separate condition. Moreover, the pilot study by McIntyre et al. (2021) observed that patients with BD had lower gut microbial α-diversity compared to healthy individuals, indicating a less varied gut microbiome. They also found a higher presence of *Clostridiaceae* bacteria in BD patients, with Collinsella specifically enriched in individuals with BD-II compared to those with BD-I, suggesting that different BD subtypes may have distinct microbiome patterns (McIntyre et al., 2021). However, due to the study's small sample size and lack of consistent grouping based on diagnosis or diet, the results need further investigation. Similarly, Painold et al. (2019) reported notable changes in gut microbiota among BD patients. They found that microbial α-diversity decreased as the duration of illness increased, meaning that the variety of gut bacteria reduced over time in individuals with BD (Painold et al., 2019). Compared to healthy controls, BD patients had higher levels of Actinobacteria and *Coriobacteria*, while healthy individuals had more *Ruminococcaceae* and *Faecalibacterium*, which are generally considered beneficial. Among BD patients with elevated levels of the inflammatory marker IL-6, there were increases in *Lactobacillales* and *Streptococcaceae*, suggesting a connection between inflammation and gut microbiota changes. The study also linked specific bacterial groups to metabolic issues in BD. For instance, *Clostridiaceae* were more abundant in patients with high cholesterol, and *Eubacterium* was associated with markers of oxidative stress. Additionally, more severe depressive symptoms were correlated with an increased presence of *Enterobacteriaceae*, while comparatively healthier BD patients had more *Clostridiaceae* and *Roseburia*. These findings support the relevance of MGBA mechanisms in BD pathophysiology, implicating both immune and metabolic pathways as shown in Table 8, and are conceptually illustrated through the microbiota–immune–metabolic–brain axis model (Figure 6; Painold et al., 2019).

**Table 8:** Summary of microbiota–immune–metabolic findings in BD.

**Study**	**Observation**	**Microbial taxa**	**Implication**
McIntyre et al. (2021)	↓Alpha-diversity in BD vs. healthy controls	–	Indicates reduced microbial diversity in BD
	↑Abundance in BD (overall)	Clostridiaceae	Potential microbial signature of BD
	↑Abundance in BD-II vs. BD-I	*Collinsella*	Suggests subtype-specific microbiome differences
Painold et al. (2019)	↓Alpha-diversity with increasing illness duration	–	Suggests progressive gut dysbiosis in BD
	↑Abundance in BD	Actinobacteria	Reflects altered gut microbial composition
	↑Abundance in BD	Coriobacteria	Reflects altered gut microbial composition
	↑Abundance in healthy controls	Ruminococcaceae	Indicates reduction of beneficial taxa in BD
	↑Abundance in healthy controls	Faecalibacterium	Indicates reduction of beneficial taxa in BD
	↑In BD with elevated IL-6	Lactobacillales	Suggests link between microbiota changes and inflammation
	↑In BD with elevated IL-6	Streptococcaceae	Suggests link between microbiota changes and inflammation
	↑In BD with high cholesterol	Clostridiaceae	Associated with metabolic disturbance in BD
	↑In BD with oxidative stress markers	*Eubacterium*	Associated with oxidative stress in BD
	↑In BD with depressive symptoms	Enterobacteriaceae	Linked to greater symptom severity
	↑In comparatively healthier BD patients	Clostridiaceae, Roseburia	May reflect relative microbial resilience

#### 3.2.3 Microbial markers and therapeutic correlates

A cross-sectional study has found that taking atypical antipsychotics (AAPs) led to an increase in a group of gut bacteria called *Lachnospiraceae* and a decrease in Akkermansia, which is often linked to gut health (Flowers et al., 2017). While there was also an initial drop in Sutterella, this result was no longer significant after adjusting for age, body mass index (BMI), and gender. AAP use was also linked to a general decrease in gut microbial diversity, especially in women. In a related finding, A related study reported that people with BD had lower levels of *Faecalibacterium*, a beneficial bacterium known for producing butyrate a short-chain fatty acid important for gut health. Higher levels of this bacterium were associated with better physical wellbeing, mood, sleep quality, and lower anxiety (Evans et al., 2017). Moreover, another study also highlighted the important role of *Faecalibacterium* in producing butyrate (Tanca et al., 2017). Independently, Sasaki et al. (2018) tested the effects of small amounts of prebiotic fibers like indigestible dextrin, α-cyclodextrin, and dextran using a lab-based colon model and a small human trial. They found that these supplements increased the production of beneficial SCFAs like acetate and propionate without changing the overall diversity or makeup of gut bacteria. This suggests that low-dose prebiotics can boost gut health without disrupting the balance of the microbiome. Another study found that patients hospitalized for acute mania were 5.5 times more likely to have used systemic antibiotics in the preceding 3 days than controls (Yolken et al., 2016). This association suggests that bacterial infections, as evidenced by antibiotic use, may contribute to manic episodes through immune activation, highlighting infection prevention and treatment as potential targets. Complementary findings demonstrated that systemic immune activation via LPS in mice produced sustained reductions in novel object exploration for up to 24 h (Haba et al., 2012). These effects were independent of acute sickness and linked to impaired cognition and motivation, particularly continuous attention and curiosity, through prolonged central amygdala activation. Although not directly tied to BD prevention, the study illustrates how transient inflammation may result in lasting behavioral changes.

#### 3.2.4 Developmental and drug-induced microbiome disruption

Disruptions to the gut microbiome during early development or the initial phases of psychiatric illness may influence the trajectory of mental disorders (Lavebratt et al., 2019) reported that early-life antibiotic exposure is linked to modestly increased risks for several childhood psychiatric disorders, including mood disorders, suggesting sensitive developmental windows for microbiome perturbations (Lavebratt et al., 2019). In first-episode psychosis, Schwarz et al. (2018) identified a distinct microbial signature with elevated *Lactobacillus* and decreased *Veillonellaceae*, correlating with greater symptom severity and poorer 12-month remission. These findings support the use of microbiome-targeted interventions early in life or illness to potentially mitigate progression of severe psychiatric conditions. Changes in the gut microbiome caused by antipsychotic medications particularly risperidone have been linked to weight gain and metabolic effects. In children receiving long-term risperidone, the ratio of Bacteroidetes to Firmicutes (two major bacterial groups in the gut) dropped significantly from 1.24 in psychiatric controls to just 0.20 in those who gained a lot of weight. This shift came with an increase in certain Firmicutes bacteria, especially *Clostridium* species and *Erysipelotrichaceae*. On the other hand, children whose weight stayed stable had higher levels of Collinsella aerofaciens (Bahr et al., 2015). Supporting this, Bahr et al. (2015) found that risperidone causes weight gain not only by affecting appetite but also by altering the gut microbiome in ways that reduce the body's ability to burn energy. In mice, risperidone treatment led to gut bacterial changes similar to those seen in obesity more Firmicutes, fewer Bacteroidetes and a 16% drop in resting energy expenditure, especially from anaerobic (non-oxygen-requiring) metabolism. Remarkably, this effect was transferable: when gut microbes or even just viral particles (phages) from risperidone-treated mice were transplanted into healthy mice, the recipients also experienced slower metabolism and weight gain. This suggests that risperidone's metabolic side effects are largely driven by changes in gut microbes, opening up the possibility of targeting the microbiome to prevent or reduce weight gain in patients taking this medication.

#### 3.2.5 Gut microbiota-based therapeutic interventions

##### 3.2.5.1 Probiotics

In a randomized trial, Dickerson et al. (2018) randomized 66 patients following hospitalization for acute mania to receive 24 weeks of adjunct treatment with *Lactobacillus rhamnosus* GG plus *Bifidobacterium animalis* subsp. *lactis* Bb12 or placebo after discharge from hospital for acute mania. Fewer rehospitalizations for psychiatric illness over the balance of the year were reported by the probiotic group than were reported by the placebo group, as well as a longer time elapsed before rehospitalization. This protective benefit was strongest among participants whose baseline systemic inflammation was at or above the fiftieth percentile. In sharp contrast, Eslami Shahrbabaki et al. (2020) carried out an 8-week double-blind trial in which patients were administered a multi-strain probiotic (*B. bifidum, B. lactis, B. longum, L. acidophilus*) with a diagnosis of bipolar I disorder and no significant differences on mania or depression rating scales were found, emphasizing that pinpointing strains and duration for treatments needs refinement. As a whole, these studies underpin further inquiry into the use of probiotic strategies as preventative and adjunctive dependent modalities for bipolar disorder, whereas further studies in humans are required to ascertain efficacy and achieve protocol optimization.

##### 3.2.5.2 Prebiotics

According to a preclinical study, Kao et al. (2018), on whether the prebiotic (B-GOS^®^) could ameliorate olanzapine-evoked weight gain while sustaining the antispychotic effects of the drug, B-GOS^®^ was found to prevent the typical olanzapine-induced weight gain in female rats. The specific metabolic benefit appears to result from gut bacteria modifications, increased Bifidobacterium and decreased Firmicutes species. B-GOS^®^ alone raised blood acetate levels (a short-chain fatty acid), but when it was combined with olanzapine, acetate levels returned to normal, while fat tissue metabolism improved through restored GPR43 receptor expression. Besides, B-GOS^®^ supplementations elevated NMDA receptor components (GluN1 protein and GluN2A mRNA) of the brain essential for cognitive functions besides raising the inflammatory marker TNFα in combination with olanzapine as a factor toward weight gain reduction. B-GOS^®^ did not, however, compromise the main olanzapine antipsychotic mechanism, which is mediated through blockage of serotonin 5-HT2A receptors. The results seem to show that B-GOS^®^ may be an effective adjunct treatment aimed at preventing metabolic side effects of antipsychotics; however, the precise role attributed to acetate in this regard still remains to be clarified.

Limited but emerging evidence suggests gut microbiota alterations in BD may affect immune and neuroinflammatory pathways influencing mood swings. Preliminary human studies associate specific microbial signatures with mood states, and probiotic interventions are under investigation for mood stabilization by reducing systemic inflammation.

### 3.3 Anxiety disorders

Gut microbiota alterations in anxiety disorders are increasingly evident in human studies, with beta-diversity consistently shifted, while alpha-diversity and taxa level changes remain heterogeneous across disorder subtypes and sex (Jiang et al., 2018; Butler et al., 2023; Kim et al., 2023). Preclinical models converge on immune–inflammatory signaling, HPA axis dysregulation and neurotransmitter alterations as recurrent mechanistic pathways (Ravi et al., 2021; Jia et al., 2024; Tyagi et al., 2025), with probiotic and prebiotic supplementation showing anxiolytic effects in randomized controlled trials via MGBA modulation (Johnstone et al., 2021; Zhu R. et al., 2023).

#### 3.3.1 Background

Anxiety disorders represent a group of persistent neuropsychiatric conditions affecting approximately 4.05% of the global population, with prevalence rising by over 55% since 1990 (Javaid et al., 2023). Despite the widespread use of serotonergic medications, benzodiazepines and cognitive behavioral therapy (CBT), therapeutic outcomes remain limited with less than 50% of patients reaching full remission and nearly one-third do not respond to conventional treatments (De Vries et al., 2016). Clinically, anxiety disorders encompass disorders such as generalized anxiety disorder (GAD), panic disorder, specific phobias, and social anxiety disorder (SAD), all marked by fear responses, behavioral avoidance, and heightened nervous arousal in perceived threatening situations (Wu et al., 2025). Yet, symptomatology is highly variable across individuals, shaped by underlying neurobiological factors. Evidence suggests a widely distributed neural circuit underpinning anxiety disorders, involving interconnected regions such as the amygdala, hippocampus, bed nucleus of the stria terminalis, insula, hypothalamus, anterior cingulate cortex and prefrontal cortex (Drzewiecki and Fox, 2024). Not all individuals exposed to stress or adversity go on to develop anxiety disorders (Charney, 2003). Vulnerability factors include early life trauma, poor social support, co-existing medical conditions, genetic predisposition and sex-based differences (Lai and Xiong, 2025).

Women are nearly twice as likely to develop anxiety disorders, potentially due to enhanced HPA axis activation under stress and modulatory effects of sex hormones on GABAergic and glucocorticoid signaling (Jaggar et al., 2020). There is a high comorbidity between the anxiety disorders and MDD (Bobo et al., 2022). Heritability estimates range between 30 and 50%, although genome-wide study results have been inconclusive (Meier et al., 2019). From a systems perspective, anxiety disorders is characterized by abnormalities in neurotransmitter functions, neurotrophic signaling, the endocannabinoid system and HPA axis function and these alterations are further compounded by chronic inflammation and maladaptive immune responses (Cai et al., 2025). Interestingly, elevated baseline levels of inflammatory markers such as CRP and IL-6 have been linked to both increased risk and severity of anxiety (Mac Giollabhui et al., 2025), while epidemiological data show that individuals with inflammatory autoimmune diseases like psoriasis have increased risks of experiencing developing anxiety disorders (Lee et al., 2025).

#### 3.3.2 MGBA and anxiety disorders

Emerging evidence highlights the MGBA as a critical contributor to anxiety pathophysiology (Abautret-Daly et al., 2018). Genetic studies further showed that several loci associated with GI disorder such as IBS, namely *NCAM1, CADM2*, and *PHF2/FAM120A* also correlate with anxiety and mood disorders (Eijsbouts et al., 2021). Moreover, Mendelian randomization analyses support a causal role for specific gut microbes: Actinobacteria and *Bifidobacterium* taxa appear protective, while *Lactobacillaceae* increase anxiety risk (Lai and Xiong, 2025). Animal research and scarce human studies confirm these findings, showing that disruptions in microbiota composition or metabolite profiles can induce anxiety-like behaviors via MGBA (Cai et al., 2025).

Gut microbiota alterations are constantly found in individuals with anxiety disorders (Chen et al., 2019). For instance, whilst beta diversity (reflecting differences in microbial community composition) is consistently altered across anxiety disorders, alpha diversity patterns show disorder type and sex-specific patterns (Jiang et al., 2018; Butler et al., 2023; Kim et al., 2023). Sex-dependent effects emerged specifically in a large Korean study with anxious population, where men exhibited reduced alpha diversity while women showed no changes (Kim et al., 2023). However, self-reported anxiety symptoms may limit the generalizability of the findings. Furthermore, a small study with GAD diagnosed patients showed reduced microbial richness among males and females (Jiang et al., 2018) while SAD patients maintained normal diversity (Butler et al., 2023). One hypothesis for these observed differences is that biological sex influences gut microbial composition through hormone-regulated immune and metabolic pathways, with sex steroid hormones and sex-linked genetic factors shaping the immune environment and selectively supporting the growth of certain microbial taxa (Jaggar et al., 2020).

#### 3.3.3 MGBA mechanisms in anxiety

SCFA producing taxa is depleted in anxiety disorders (Burton et al., 2023), mirroring patterns observed in other psychiatric conditions (Bruun et al., 2024). Anxious men showed reduced abundance of butyrate-producing *Lachnospiraceae_NK4A136* (Kim et al., 2023), which is essential for epithelial barrier integrity and microglial regulation (Erny et al., 2015). GAD patients demonstrated broader SCFA depletion, with decreased levels of *Faecalibacterium, Eubacterium rectale, Butyricicoccus*, and *Lachnospira*, bacteria integral to maintaining mucosal homeostasis and modulating host-microbiota immune interactions. Interestingly, in the remission state, increased levels of *Faecalibacterium* and *Eubacterium rectale* were observed (Jiang et al., 2018). Similarly, SAD control group showed an abundance of *Parasutterella*—a symbiont that supports intestinal mucosal stability with a potential protective role in anxiety (Butler et al., 2023; Yao et al., 2023).

Pro-inflammatory and pathobiont taxa showed disorder-specific enrichment patterns. GAD patients exhibited increased *Bacteroides, Ruminococcus gnavus*, and *Fusobacterium* (Jiang et al., 2018), with the latter linked to epithelial disruption, gut inflammation and IBS pathogenesis (Zhou, 2016). Interestingly, about one third of people with IBS have anxiety symptoms, suggesting a potential shared gut–brain inflammatory axis between GAD and IBS (Grover et al., 2021). SAD patients showed enrichment of novel genera *Anaeromassilibacillus* and *Gordonibacter*, whereas *Anaeromassilibacillus* was linked to autism spectrum disorder—a condition with high SAD comorbidity (Butler et al., 2023). Interestingly, the gut metabolic module aspartate degradation I, which converts L-aspartate to oxaloacetate by aspartate aminotransferase, was elevated in SAD patients and the authors hypothesized that bacterial aspartate aminotransferase enzyme activity may represent a link between gut microbiome function and the tryptophan-kynurenine pathway, a key physiological system in psychiatric disorders (Butler et al., 2023). The tryptophan-kynurenine pathway functions as a stress-activated metabolic system that diverts tryptophan from serotonin synthesis toward kynurenic acid production, which acts as a glutamate receptor antagonist and may contribute to anxiety pathophysiology through altered glutamatergic neurotransmission (Butler et al., 2022). Despite consistent patterns of SCFA depletion, the heterogeneity in microbial signatures across anxiety subtypes suggests disorder-specific pathophysiological mechanisms. Small sample sizes and different anxiety types limit clinical translation. Future studies should standardize methodological approaches across populations and increase sample sizes.

#### 3.3.4 MGBA alterations and pathophysiological mechanisms in anxiety

Rodent models of anxiety disorders provide causal evidence that stress exposure disrupts the MGBA, leading to microbiota alterations, compromised gut barrier integrity, immune activation and neurotransmitter imbalance (Bear et al., 2021). Alterations in the Firmicutes and Bacteroidetes (F/B) ratio—a key marker of gut dysbiosis—have been reported in male mice exposed to chronic mild stress (CMS; Tyagi et al., 2025) and humid heat environment stress models (Weng et al., 2024) Enrichment of the phylum Proteobacteria was observed across multiple stress paradigms (Jia et al., 2024; Tyagi et al., 2025). Proteobacteria enrichment in the gut serves as a dysbiosis marker (Shin et al., 2015). Moreover, 6 weeks of chronic stress also revealed decreased relative abundance of Bacteroidetes and *Deferribacteres* (Jia et al., 2024).

Across preclinical anxiety models, there was a consistent enrichment of pathogenic or dysbiosis-associated bacteria. Male mice exposed to CMS for 2 weeks showed increased pathogenic bacteria including *Acinetobacter, Proteus*, and *Enterococcus* (Tyagi et al., 2025). Similarly, chronic stress elevated *Kineothrix alysoides* and *Helicobacter bilis* compared to controls (Jia et al., 2024). Similarly, a systematic depletion of beneficial and anti-inflammatory taxa, many of which are involved in SCFA production, was observed in stress-exposed animals. *Lactobacillus murinus, L. intestinalis, L. reuteri*, and *Akkermansia muciniphila* (Weng et al., 2024). *Oscillibacter, Muribaculum, Roseburia*, and *Alistipes* (Tyagi et al., 2025) as well as *Muribaculum intestinale, Ligilactobacillus murinus, Duncaniella*, and *Prevotella* (Jia et al., 2024). These losses occurred irrespective of stress model or duration. These microbial alterations were accompanied by functional impairments, with CMS significantly reducing SCFA production, particularly acetic and propionic acid concentrations (Tyagi et al., 2025).

Anxiety involves MGBA alterations that create cascading pathophysiological changes (Rogers et al., 2016). Chronic stress activates the HPA axis, resulting in increased cortisol production (Faravelli, 2012). Mice exposed to chronic unpredictable mild stress (CUMS) for 6 weeks showed increased hypothalamic corticotropin-releasing hormone concentrations, elevated corticosterone levels and increased blood ammonia, which corresponded with anxiety-like behaviors, demonstrating HPA axis involvement in anxiety pathogenesis (Jia et al., 2024). Interestingly, Ammonia (a neurotoxic by-product) can impair the nervous system even at low levels and it has been associated with anxiety disorders—potentially serving as a biomarker (Duan et al., 2015). Furthermore, chronic stress impairs inhibitory neurotransmitter systems. Both GABA and serotonin availability declined with reduced receptor expression for GABA_A_α2 and GABAB1β in prefrontal cortex in the 2 week CMS (Tyagi et al., 2025). Dysregulation in these receptors is linked to anxiety behaviors and it may be considered as a potential therapeutic pathway in anxiety treatment (Solati et al., 2013). Moreover, 6 week exposure to CUMS showed further GABA level reductions in tissues such as hippocampus, blood and feces, suggesting that more widespread neurotransmitter depletion may be stress type and duration dependant (Jia et al., 2024). Furthemore, an increased serotonin metabolism was demonstrated by diminished hypothalamic serotonin levels, lowered 5-hydroxytryptamine (5-HT)/tryptophan ratios and elevated 5-hydroxyindoleacetic acid/5-HT ratios, indicating dysregulated serotonergic system (Jia et al., 2024). Altered serotonergic signaling is considered a hallmark neurochemical feature of anxiety disorders (Yohn et al., 2017).

**Figure 7:**
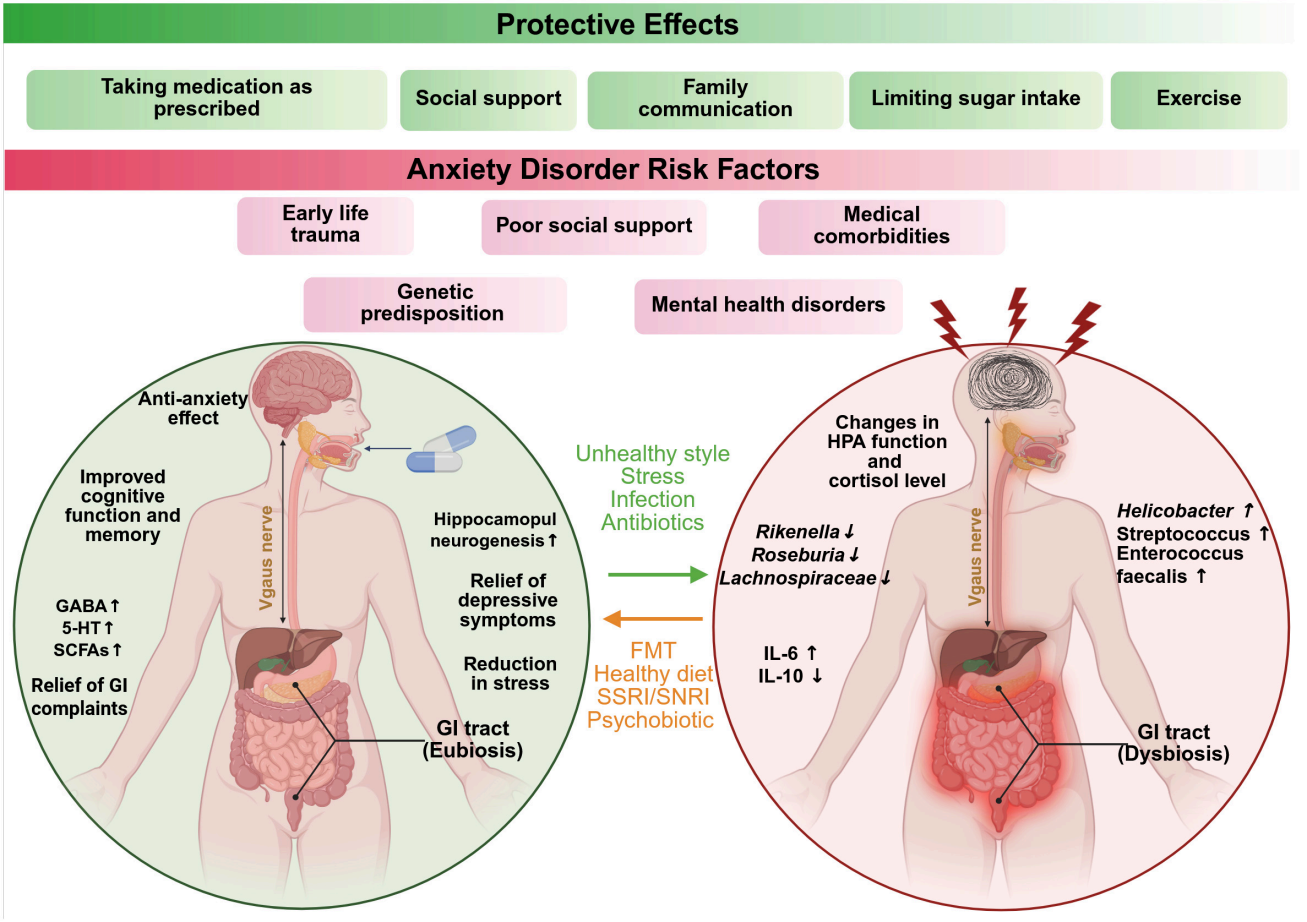
Summarizes the known risk factors in anxiety disorders. It demonstrates the various microbiome–gut–brain signaling pathways that encompass those physiological systems involved in the pathogenesis of anxiety and stress-related conditions (5-HT, 5-hydroxytryptamine; GABA, gamma-aminobutyric acid; SCFA, short-chain fatty acids; HPA, hypothalamic–pituitary–adrenal axis; FMT, fecal microbiota transplantation; SSRIs, selective serotonin reuptake inhibitors; SNRIs, serotonin and norepinephrine reuptake inhibitors). Created with BioRender.com.

Moreover, chronic stress exposure triggers systemic inflammatory activation through upregulation of pro-inflammatory cytokine cascades (Ravi et al., 2021). Elevated IL-6 and TNF-α concentrations in CMS exposed mice demonstrate stress-induced inflammatory signaling contribution toward anxiety-like behaviors (Tyagi et al., 2025). Similarly, mice exposed to CUMS for 6 weeks showed an increased interferon-gamma (IFN-γ) and decreased anti-inflammatory IL-10 levels, demonstrating anti-inflammatory suppression with further pro-inflammatory activation (Jia et al., 2024). Intriguingly, correlation analyses linked microbiota changes to alterations in stress hormones, neurotransmitters, inflammatory markers and anxiety-like behaviors, suggesting a gut–brain contribution to the observed behavioral manifestations in anxiety pathology (Jia et al., 2024; Tyagi et al., 2025; Figure 7).

#### 3.3.5 Microbiota-based interventions

##### 3.3.5.1 Psychobiotics

Psychobiotic interventions targeting the MGBA show promise for reversing stress-induced behavioral and physiological dysfunctions. A 4 week treatment with GABA-producing psychobiotic *Limosilactobacillus reuteri* reversed anxiety-like behaviors and MGBA disruption in male mice (Tyagi et al., 2025). The treatment normalized serum corticosterone levels, enhanced central GABA and serotonin concentrations and reduced peripheral inflammation and gut microbiota profiling revealed a reduction in potentially pathogenic genera (*Acinetobacter, Enterococcus, Bacillus*) and increased abundance of beneficial taxa (*Muribaculum, Alistipes, Lactobacillus*), indicating the therapeutic potential of targeted psychobiotic intervention for stress-related disorders (Tyagi et al., 2025). Interestingly, the treatment demonstrated dose-dependent therapeutic effects, with moderate dosages proving more effective than high doses (1,000 mg/kg), possibly due to pharmacodynamic principles, where excessive concentrations saturate biological pathways and diminish therapeutic outcomes (Tyagi et al., 2025; Figure 7). However, it is not clear whether the effects were long lasting. In a randomized trial, psychobiotic *Lactobacillus plantarum JYLP-326* administered for 3 weeks reduced anxiety, depression and insomnia symptoms in test-stressed college students (Zhu R. et al., 2023). 16S rRNA profiling revealed enrichment of SCFA-producing genera *Bifidobacterium* and *Prevotella*, supporting microbiota-mediated MGBA modulation (Zhu R. et al., 2023). However, the short intervention period and reliance on self-reported outcomes limit translational strength.

##### 3.3.5.2 Probiotics

In mice model of humid heat–induced anxiety, oral administration of probiotic *Lactobacillus murinus* for 14 days significantly reduced anxiety-like behaviors by restoring gut microbiota composition, which further downregulated neuroinflammatory markers (Weng et al., 2024). Similarly, mice treated with probiotic *Lactiplantibacillus plantarum* D-9 for 14 days showed reversed anxiety-like behaviors (Jia et al., 2024). The probiotic restored gut microbial diversity, increasing *L. murinus, L. johnsonii* and OTU richness. It has also regulated tryptophan–serotonin metabolism, modulated the HPA axis and shifted inflammatory markers (reduced IL-6, IFN-γ and increased IL-10), supporting the effect of probiotic on reducing anxiety-like behavior via MGBA regulation (Jia et al., 2024).

##### 3.3.5.3 Dietary interventions

Dietary strategies such as tryptophan supplementation may exert anxiolytic effects through gut brain axis. A tryptophan-rich diet for 35 days reduced anxiety-like behaviors in CUMS through gut–brain axis modulation (Weng et al., 2024). Tryptophan is an essential amino acid obtained solely from the diet and serves as a precursor for serotonin synthesis and it can produce seratonin via tryptophan hydroxylase 1 activity in the intestinal enterochromaffin cells (Jenkins et al., 2016). Treatment restructured gut microbiota by increasing beneficial *Lachnospiracea, Clostridium, Lactobacillus*, and *Bifidobacterium* while reducing pathogenic *Escherichia*. Tryptophan increased serum 5-HT levels, enhanced brain BDNF expression, reduced neuroinflammation, improved mitochondrial energy metabolism and restored gut barrier integrity by upregulating tight junction proteins (Weng et al., 2024; Figure 7). Evidence for gut microbiota involvement in anxiety disorders is consistent across human studies and strongly supported by preclinical mechanistic data, with therapeutic interventions showing early promise in randomized controlled trials, yet still requiring a large-scale validation.

### 3.4 Post-traumatic stress disorder (PTSD)

Gut microbiota alterations in PTSD are increasingly evident in human cohorts, with consistent depletion of SCFA-producing taxa and enrichment of inflammatory pathobionts, while taxa level signatures vary across geography and trauma type (Bajaj et al., 2019; Ke et al., 2023; Yirmiya et al., 2024; Li Y. I. et al., 2025). Preclinical PTSD models consistently implicate gut barrier dysfunction, immune–inflammatory activation, HPA axis dysregulation and neurotransmitter imbalance as key mechanistic through-lines (Sherin and Nemeroff, 2011; Tanelian et al., 2023; Yadav et al., 2023). Prebiotic supplementation has shown sex-specific improvement in PTSD symptoms, linked to enhanced SCFA production and gut microbiota modulation in males (Voigt et al., 2025).

#### 3.4.1 Background

PTSD is a chronic neuropsychiatric condition affecting 1.3–12.2% of the global population (Hu et al., 2024). PTSD is triggered by exposure to a traumatic event involving actual or threatened death, injury or sexual violence (Al Jowf et al., 2023). Remission is achieved by less than 30% of patients and pharmacological interventions remain slow-acting and lack effectiveness (Kelmendi et al., 2016). Core symptoms span intrusive memories, hyperarousal, avoidance and negative shifts in mood and cognition (Petakh et al., 2024), yet clinical presentation is highly heterogeneous—shaped by trauma type and individual neurobiology (Huckins et al., 2020). Structural and functional abnormalities in the amygdala, hippocampus and medial prefrontal cortex underlie persistent, overgeneralized fear responses (Harnett et al., 2020). Not everyone exposed to trauma would develop PTSD. Risk of developing PTSD is influenced by adverse childhood experiences, younger age and socioeconomic disadvantage (Xue et al., 2015) with prevalence rates twice as high in females than in males (Perrin et al., 2014). Furthermore, heritability estimates range from 30 to 40% (Girgenti et al., 2021) with genome-wide studies implicating immune-linked loci such as *ANKRD55* among others (Stein et al., 2016; Ugidos et al., 2019). At the systems level, PTSD is characterized by heightened peripheral inflammation, impaired regulatory T cell function (Jergović et al., 2014), dysregulation of the HPA axis, neurotransmitter systems and neuroinflammation (Michopoulos et al., 2015; Aliev et al., 2020). Elevated pre-existing inflammation has emerged as a predictive factor for PTSD onset. Individuals with high baseline CRP are more likely to develop PTSD symptoms following trauma exposure (Eraly et al., 2014).

#### 3.4.2 Microbiome alterations in PTSD patients

A growing body of evidence implicates the MGBA as a key modulator of PTSD vulnerability. Given that gut microbiota regulate immune function and inflammation, both key features of PTSD pathophysiology, this axis represents a promising therapeutic target. Early-life gut microbiota imbalances may predispose individuals to PTSD susceptibility after trauma exposure (Leclercq et al., 2016). Supporting this hypothesis, PTSD is more prevalent in individuals with GI inflammatory disorders including, IBD and Crohn's disease (Katrinli et al., 2022; Kent, 2024). Recent Mendelian randomization findings further validate this framework: *Dorea* and *Sellimonas* appear protective, whereas *Phascolarctobacterium* and *Ruminococcaceae UCG-004* are associated with increased PTSD risk (He et al., 2024). Multiple clinical studies have characterized the gut microbiome in PTSD patients, revealing patterns of microbial dysbiosis (Hemmings et al., 2017; Bajaj et al., 2019; O'Hare et al., 2024; Yirmiya et al., 2024; Li Y. I. et al., 2025). An early study found decreased abundance of Actinobacteria*, Lentisphaerae* and Verrucomicrobia in PTSD patients vs. trauma-exposed controls (Hemmings et al., 2017). Decreased Verrucomicrobia (which includes the beneficial bacterium *Akkermansia muciniphila*, important for gut barrier function) correlated with greater PTSD symptom severity in 26 Iraq/Afghanistan War Veterans from different ethnic backgrounds, though findings were based on self-reported behavioral symptoms (Li Y. I. et al., 2025).

SCFA-producing bacteria are consistently depleted among PTSD patients along with increased pathobionts indicate a shift toward inflammatory environment. SCFA producing *Lachnospiraceae* and *Ruminococcaceae* were reduced in US combat veterans with cirrhosis and PTSD compared to those without PTSD, together with increased pathobionts *Enterococcus* and *Escherichia/Shigella* (Bajaj et al., 2019), suggesting these microbial changes may reflect or even exacerbate inflammation-driven psychiatric vulnerability. Moreover, a longitudinal study following war-exposed Israeli children for over 15 years found that clinically diagnosed PTSD youth exhibited significantly lower α-diversity, decreased SCFA-producing bacteria (*Dialister, Eubacterium*) and increased pro-inflammatory pathobionts (*Collinsella, Veillonella*) compared to controls, along with increased nitrate reductase-associated metabolites linked to nitrogen metabolism pathways. Fecal microbiome transplantation from PTSD adolescents into germ-free mice induced significant anxiety behaviors, confirming functional contribution of gut microbiota to psychiatric symptomatology (Yirmiya et al., 2024). However, the study used only one behavioral test (Elevated Plus Maze). Future studies should employ multiple paradigms to capture the broader spectrum of PTSD-related phenotypes. These findings collectively highlight that PTSD is associated with reproducible patterns of gut dysbiosis, characterized by reduced SCFA-producing taxa and increased inflammatory pathobionts, supporting a functional link between microbial imbalance and psychiatric vulnerability.

Bacteria changes may be linked to geographic location where the study was carried. PTSD-diagnosed South African individuals showed higher levels of *Mitsuokella, Odoribacter, Olsenella*, and *Catenibacterium* compared to controls, positively correlating with childhood trauma severity. Intriguingly, these four genera are found in oral microbiota of patients with periodontitis. The authors hypothesized that early life adversity may alter oral microbiome composition, which can shift to gut via saliva, potentially creating a pro-inflammatory state that increases vulnerability to PTSD following subsequent trauma exposure (Malan-Muller et al., 2022). *Catenibacterium* was also identified in another South African study with self-assessed PTSD symptoms (O'Hare et al., 2024), indicating potential geographic location-specific associations (Parizadeh and Arrieta, 2023). Higher levels of *Catenibacterium, Collinsella*, and *Holdemanella* also correlated with higher symptom severity (O'Hare et al., 2024). These bacteria produce lactic acid. Lactate (the ionized active form of lactic acid) has been linked to PTSD through unclear mechanisms. One proposed mechanism is that lactate enhances hippocampal neuronal firing, potentially contributing to panic symptoms (Bergold et al., 2009). Moreover, elevated *Holdemanella* has been associated with reduced right amygdala and hippocampal volumes (Wanapaisan et al., 2022) brain regions consistently reduced in PTSD individuals (Ben-Zion et al., 2023). Future functional studies should assess whether associations between these bacteria concentrations and brain volumes reflect causal relationships. Together, these findings suggest that microbiome shifts associated with trauma exposure and geography may influence PTSD risk by modulating brain structure and neuroinflammatory signaling.

Additional population studies have expanded these findings. Whole-genome sequencing of 191 American nurses identified specific bacterial alterations in self-reported PTSD: increased *ParaBacteroides goldsteinii, Barnesiella intestinihominis*, and *Paraprevotella* unclassified, decreased *Eubacterium eligens* and *Akkermansia muciniphila* (Ke et al., 2023).

Differences in findings could be attributed to the fact that participants were from different geographic locations, which are known to influence gut microbiota composition through factors such as diet, environment, and lifestyle-potentially contributing to the observed variation. Furthermore, small sample sizes, different trauma types and symptom assessment methods limit clinical translation. Future studies should standardize methodological approaches across populations and increase sample sizes in order to distinguish between geographic and pathological microbiome variations.

#### 3.4.3 Mechanistic insights from preclinical models—barrier, immune, and neurotransmitter changes

Rodent models of PTSD provide causal evidence that psychological trauma disrupts the MGBA, leading to microbiota alterations, compromised gut barrier integrity, immune activation and neurotransmitter imbalance. Alterations in the Firmicutes and Bacteroidetes (F/B) ratio—a key marker of gut dysbiosis, have been reported in PTSD model rodents, exposed to psychological trauma using Repeated Social Defeat Stress and Single Prolonged Stress (SPS; Zhou et al., 2020; Yadav et al., 2023).

Across preclinical PTSD models, there is consistent enrichment of pathogenic or dysbiosis-associated bacteria. For instance, Zhou et al. (2020) reported elevated levels of *Cyanobacteria* and Proteobacteria in SPS exposed male rats. Similarly, female rats exposed to trauma exhibited increased abundance of *Anaerovorax* and *Flavonifractor*, latter being associated with GABA disruption (Tanelian et al., 2023). In male mice, Laudani et al. (2023) observed elevated *Ruminococcaceae* and *Lachnospiraceae* families—taxa known to influence dopaminergic signaling and myelination via production of the neurotoxic metabolite *p*-cresol, a compound implicated in PTSD related neuropathology (Torrisi et al., 2019; Jak et al., 2020). Similarly, a systematic depletion of beneficial and anti-inflammatory taxa was observed in trauma exposed animals. In female rats, trauma led to reduced abundance of *Bifidobacterium, Clostridium sensu stricto, Turicibacter*, and *Barnesiella*—genera some of which are involved in short-chain fatty acid (SCFA) production and serotonergic (*Turicibacter*) regulation (Laudani et al., 2023; Tanelian et al., 2023) reported reduced levels of Actinobacteria, Proteobacteria, Verrucomicrobia and *Bacteroides* in male mice with PTSD-like behaviors, consistent with findings from human studies linking similar depletions to PTSD symptomatology (Hemmings et al., 2017). Interestingly, protective baseline microbiome signatures were identified in stress-resilient rodents. Females resilient to trauma exhibited higher pre-trauma levels of *Roseburia, Oscillibacter*, and *Lachnospiraceae*, suggesting that baseline enrichment of SCFA-producing bacteria may confer resistance to PTSD onset (Tanelian et al., 2023).

PTSD involves gut–brain axis alterations that create cascading pathophysiological effects. Altered regulation of the HPA axis has been observed in PTSD pathology. Female rats exposed to predator-based psychosocial stress exhibited significantly reduced baseline corticosterone levels and enhanced dexamethasone suppression compared to controls, demonstrating that PTSD is characterized by altered HPA axis function (Zoladz et al., 2021). Furthermore, gut microbiota regulates HPA axis responsiveness. Germ-free mice exhibited exaggerated plasma corticosterone responses compared to specific pathogen-free controls, demonstrating that microbiota regulates HPA axis function (Vagnerová et al., 2019). Moreover, psychological trauma compromises gut barrier function and increases inflammation through stress hormone signaling (Sherin and Nemeroff, 2011). In PTSD mice models, trauma exposure increased tyrosine hydroxylase expression (the rate-limiting enzyme in catecholamine biosynthesis), elevating stress hormones epinephrine and norepinephrine in serum and colon, which upregulated claudin-2 expression, confirming gut barrier dysfunction (Yadav et al., 2023). Trauma exposure also induces intestinal inflammation (increased CD45^+^ leukocytes, CD68^+^ macrophages, and CD3^+^ T cells and upregulated phosphorylated Stat3 and NF-κB) signaling. This shows the causal pathway whereby psychological trauma compromises gut barrier function and intestinal inflammation through stress hormone signaling. The study hypothesized that pro-inflammatory gut environment further promotes changes in microbiota (Yadav et al., 2023). Furthermore, significantly elevated branch-chain fatty acids (BCFA), which are metabolic products of protein breakdown have been hypothesized to induce epithelium inflammation (Aguirre et al., 2016).

These microbial alterations coincided with PTSD symptomatology, BBB dysfunction and hippocampal neuroinflammation, which was confirmed by increased proinflammatory cytokine levels IL-1β and IL-6 (Tanelian et al., 2023). Collectively, these studies confirm that PTSD involves gut–brain axis disruption.

Alterations in gut microbiota can disrupt neurotransmitter homeostasis, particularly affecting serotonin (5-HT), norepinephrine (NE), and dopamine (DA) systems that are dysregulated in PTSD (Zhou et al., 2020). Microbial shifts in a PTSD male rat study coincided with a significant decrease in brain 5-HT and increases in DA and NE, indicating a potential hyperaroused state. Spearman correlation analysis linked the microbiota changes to fear- and anxiety-like behaviors, suggesting a gut–brain contribution to the observed neurotransmitter imbalance in PTSD rat models (Zhou et al., 2020). Intriguingly, serotonin regulation may be particular sensitive to the decreased levels of *Turicibacter* (particularly *T. sanguinis*) as this genus influences gut-derived serotonin (Tanelian et al., 2023), which is partly controlled by the microbiota (Fung et al., 2017) and is known contributor to PTSD symptoms (Tanelian et al., 2023). Similarly, trauma induced increased *Flavonifractor* may potentially compromise GABA availability, since these bacteria primarily utilize GABA for metabolism (Tanelian et al., 2023) potentially affecting this key inhibitory neurotransmitter and contributing to anxiety symptoms observed in PTSD (Strandwitz et al., 2018). Similarly, dopaminergic dysfunction was observed. Male rats with PTSD-like behaviors showed elevated *p*-cresol (a neurotoxic metabolite linked to overgrowth of *Ruminococcaceae* and *Lachnospiraceae*) in prefrontal cortex, leading to dopaminergic dysfunction with increased DA, increased dopamine metabolite 3,4-dihydroxyphenylacetic acid (DOPAC), and significantly elevated DA D3 receptor expression compared to controls (Laudani et al., 2023). The prefrontal cortex is highly sensitive to DA and even minor fluctuations can impair prefrontal cortex-dependent functions, making it particularly relevant in PTSD, as it plays a central role in suppressing fear responses, regulating stress reactivity, and enabling emotional control (Cools and D'Esposito, 2011). Collectively, these findings demonstrate that psychological trauma initiates a cascade of interconnected MGBA disruptions in PTSD, encompassing HPA axis dysregulation, microbial dysbiosis, compromised gut barrier integrity, chronic inflammation and neurotransmitter imbalances across serotonergic, dopaminergic, and GABAergic systems. Collectively, these findings demonstrate that PTSD arises through a complex interplay of gut dysbiosis, neuroendocrine disruption, immune activation, and altered neurotransmission, firmly implicating the microbiota–MGBA in the pathophysiology of trauma-related disorders.

#### 3.4.4 Gut microbiota-based therapeutic interventions

##### 3.4.4.1 Probiotics

Therapeutic targeting of the gut–brain axis shows promise for PTSD treatment across multiple intervention modalities. A multi-strain probiotic formulation containing *Streptococcus faecalis, Clostridium butyricum, Bacillus mesentericus*, and *Lactobacillus sporogenes*, administered for 14 days reversed PTSD-like behaviors via MGBA restoration in mice (Khan et al., 2025). Treatment increased beneficial *Bacteroides acidifaciens* and reduced pathogenic *Clostridiales bacterium* and Proteobacteria. It also normalized intestinal permeability, restored cortical BDNF to control levels and significantly improved behavioral parameters in PTSD-model male mice, confirming probiotic efficacy via MGBA modulation. However, the durability of these neuroprotective effects remains unclear, as the study did not evaluate whether benefits persisted after probiotic discontinuation. Future work should adopt longitudinal designs to assess therapeutic effects across temporal windows. Furthermore, an anti-inflammatory probiotic *Lactobacillus reuteri*, administered over 8 weeks reduced CRP and stress-induced heart rate in Veterans with PTSD and mild traumatic brain injury compared to placebo (Brenner et al., 2020). Given that elevated CRP is a putative predictor of PTSD development (Eraly et al., 2014) reductions observed may reflect therapeutic potential. However, no significant changes in gut microbiota composition were detected (Brenner et al., 2020).

##### 3.4.4.2 Prebiotics

In a male rat model of PTSD-like symptoms induced via amygdala hyperactivation (a key pathophysiological mechanism of PTSD)−3 weeks of treatment with the prebiotic GOS restored microbial homeostasis by enriching beneficial *Bifidobacterium animalis* and *Limosilactobacillus reuteri*, while eliminating harmful *Enterococcus casseliflavus* (Ruciński et al., 2025). This was accompanied by reduced TNF-α and elevated IL-10, indicating suppressed inflammation and corresponding reductions in anxiety-like behavior. Intriguingly, GOS upregulated IL-10 more effectively than Citalopram, a selective serotonin reuptake inhibitor (Miranda et al., 2025) highlighting its therapeutic promise via the MGBA pathway. However, sex differences were not assessed, and prebiotic efficacy may vary by sex. Such gender differences were observed in a 12-week clinical trial combining prebiotic supplementation (20 g/day fiber) with CBT, where only men PTSD veterans exhibited increased abundance of SCFA-producing taxa such as *Bifidobacterium* and *Faecalibacterium*, with no microbiota changes observed in female participants (Voigt et al., 2025). Notably, symptom improvements in prebiotic-treated males persisted at weeks 2 and 12, contrasting with transient effects in placebo-treated males (Voigt et al., 2025). This sustained benefit aligned with *Bifidobacterium* enrichment in prebiotic recipients, vs. reductions in the placebo group, suggesting that prebiotics may serve as a sex-specific adjunct therapy in PTSD, particularly for males (Voigt et al., 2025).

##### 3.4.4.3 Dietary interventions

**Figure 8:**
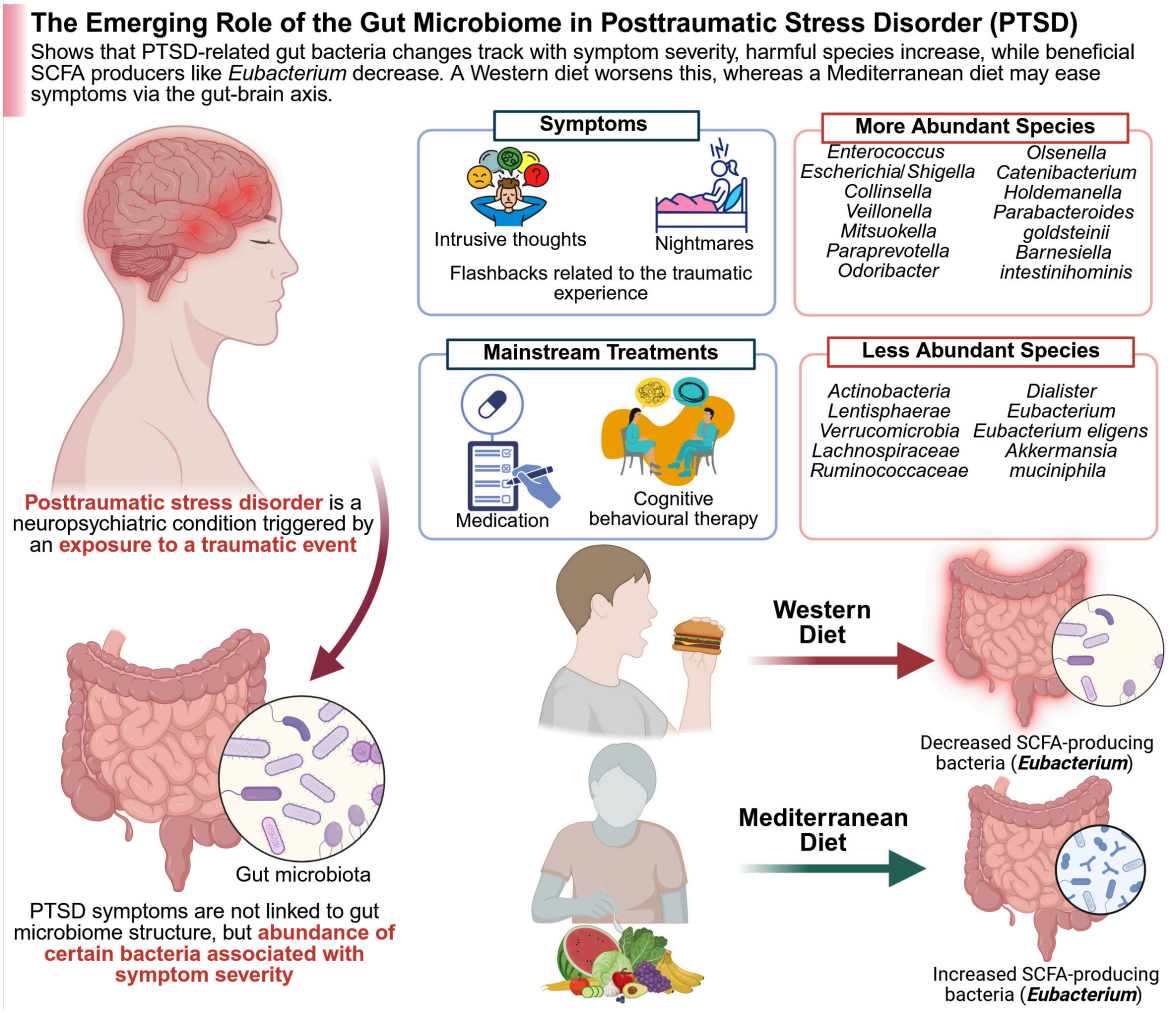
PTSD severity is linked to gut microbiota imbalances, with increased harmful bacteria and reduced beneficial SCFA producers, influenced by dietary patterns. Created with BioRender.com.

MeDi interventions also demonstrate efficacy in PTSD management (Figure 8). A 10-week MeDi intervention in World Trade Center responders with PTSD significantly reduced inflammatory markers such as CRP and lessened PTSD symptom severity compared to standard nutritional advice (Arcan et al., 2024). Similarly, women with higher adherence to the MeDi exhibited greater abundance of the PTSD-protective bacterium *Eubacterium eligens*, a short-chain fatty acid producer from dietary fiber, coinciding with lower PTSD severity, indicating that Mediterranean dietary patterns may support gut-mediated neuroprotection (Ke et al., 2023). Collectively, evidence for MGBA involvement in PTSD is consistent across human and preclinical studies, with strong mechanistic convergence, while interventional findings, despite early promise, remain preliminary and require validation in larger, controlled trials.

### 3.5 Chronic fatigue syndrome (CFS)

A growing body of clinical evidence shows that the gut microbiota plays a critical role in CFS, with consistently observed reductions in butyrate-producing bacteria and enrichment of *Enterobacteriaceae*, together with frequent depletion of anti-inflammatory taxa such as *Faecalibacterium* (König et al., 2021; Xiong R. et al., 2023).

#### 3.5.1 Background

CFS, also known as Myalgic Encephalomyelitis (CFS/ME), is a complex disorder marked by persistent fatigue, cognitive impairment, and a range of physical symptoms that are not alleviated by rest (König et al., 2021; Wang J.-H. et al., 2024). The underlying mechanisms are multifactorial and not fully understood, but increasing evidence highlights the gut–brain axis and the intestinal microbiome as critical contributors to disease onset and progression (He et al., 2023; El-Sehrawy et al., 2025). Recent advances in microbiome research have opened new avenues for prevention and intervention, focusing on how gut microbial communities influence immune, metabolic, and neural pathways relevant to CFS/ME. Taken together, these studies underscore the potential of gut-targeted therapies in managing the multi-systemic symptoms of CFS/ME.

#### 3.5.2 Microbiome alterations and the gut–brain axis in CFS/ME

Multiple studies have established that individuals with CFS/ME exhibit significant alterations in their gut microbiome composition compared to healthy controls. Dysbiosis in CFS/ME is characterized by reduced microbial diversity and a shift in the abundance of specific bacterial taxa. For example, patients often have lower levels of anti-inflammatory bacteria such as *Faecalibacterium* and higher proportions of pro-inflammatory species, which may contribute to systemic inflammation and symptom severity (König et al., 2021; Xiong R. et al., 2023). A recent meta-analysis confirmed that CFS/ME is associated with distinct gut microbial signatures, including reduced levels of butyrate-producing bacteria and increased abundance of *Enterobacteriaceae* (Jurek and Castro-Marrero, 2024). These alterations are thought to impact gut barrier integrity and immune function, both of which are implicated in CFS/ME pathophysiology. In CFS/ME, disruptions in this axis are believed to play a central role in the manifestation of fatigue, cognitive dysfunction, and mood disturbances (König et al., 2021). Microbial metabolites such as SCFAs and tryptophan catabolites can influence brain function directly or via modulation of systemic inflammation. For instance, butyrate, an SCFA produced by certain gut bacteria, supports the integrity of the intestinal barrier and exerts anti-inflammatory effects (Guo et al., 2023; Jurek and Castro-Marrero, 2024). Reduced butyrate levels in CFS/ME patients may contribute to increased gut permeability (“leaky gut”), allowing microbial products like LPS to enter the circulation and trigger systemic immune activation (Wang J.-H. et al., 2024). This immune activation may, in turn, affect the CNS, exacerbating fatigue and cognitive symptoms. This highlights the complexity of host–microbe interactions in CFS/ME and underscores the importance of personalized medicine approaches.

#### 3.5.3 Microbial metabolites, immune modulation, and disease progression

Altered microbial metabolism in CFS/ME extends beyond SCFAs. Dysbiosis can affect the kynurenine pathway of tryptophan metabolism, leading to an imbalance in neuroactive metabolites that influence mood, cognition, and pain perception (He et al., 2023). The gut microbiome modulates the balance of pro- and anti-inflammatory immune responses, which is crucial in light of the chronic low-grade inflammation observed in many CFS/ME patients (Seton et al., 2023). Recent multi-omics studies have revealed that patients with CFS/ME, especially in early disease stages, display pronounced disruptions in both gut microbial composition and metabolic profiles (Wang J.-H. et al., 2024). These disruptions include a reduction in SCFA producers and an increase in bacteria associated with inflammation and metabolic endotoxemia, which may serve as potential biomarkers for disease progression and severity. As the disease progresses, some patients may experience a partial restoration of microbial balance (eubiosis); however, this restoration does not consistently correlate with clinical improvement, highlighting the importance of early intervention (Seton et al., 2023). Moreover, GI comorbidities such as IBS are frequently observed in CFS/ME, further supporting the hypothesis that gut microbiota imbalances are not merely a consequence of illness but may actively contribute to its pathogenesis (He et al., 2023). In addition, emerging data suggest that specific microbial taxa may be causally linked to CFS/ME risk. For example, reductions in *Ruminococcaceae* and increases in *Paraprevotella* have been associated with greater disease severity (Guo et al., 2023). Notably, animal models have demonstrated that transferring dysbiotic microbiota from CFS/ME patients to germ-free mice can induce fatigue-like behaviors, supporting a causal role for the microbiome in disease pathogenesis (Bansal et al., 2025). Taken together, these studies demonstrate that alterations in the gut microbiome and its metabolic products are consistently associated with immune dysregulation and symptom severity in CFS/ME, suggesting that targeting the microbiome may offer promising new avenues for diagnosis and treatment.

#### 3.5.4 Gut microbiota-based therapeutic interventions

##### 3.5.4.1 Probiotics

Given these findings, probiotic supplementation, particularly with strains of *Lactobacillus* and *Bifidobacterium*, has shown potential to restore microbial balance, enhance SCFA production, and reduce inflammation in CFS/ME patients (Carrasco-Querol et al., 2024; Skjevling et al., 2024). Furthermore, multistrain protocols, including *Saccharomyces boulardii* and *Lactobacillus acidophilus*, have demonstrated reductions in fatigue and improvements in cognition and mood (Bourgonje et al., 2024; Stallmach et al., 2024). Synbiotics, combinations of probiotics and prebiotics, are also promising. For instance, randomized controlled trial using a synbiotic formulation containing *Lacticaseibacillus rhamnosus, Lactiplantibacillus plantarum, Bifidobacterium lactis, Bifidobacterium longum*, fructo-oligosaccharides, and zinc showed significant reductions in post-exertional malaise and increased brain metabolites in post-COVID-19 CFS patients (Varesi et al., 2021; Hsu et al., 2025).

##### 3.5.4.2 Dietary intervention

Dietary patterns rich in plant-based fibers, polyphenols, and fermented foods support a diverse and resilient microbiome. Several studies have reported symptomatic improvements in CFS/ME patients who adopt Mediterranean-style diets or increase their intake of prebiotic-rich foods (Kitami et al., 2020; Lupo et al., 2021). Conversely, high-fat, low-fiber diets are associated with worsened dysbiosis and increased inflammation.

##### 3.5.4.3 Prevention and intervention

A key consideration is that overuse of antibiotics, particularly in childhood, can disrupt the developing microbiome and may increase susceptibility to CFS/ME later in life. Preventive strategies should emphasize prudent antibiotic use (Ranisavljev et al., 2024). Additionally, therapeutic approaches targeting microbial metabolites, such as SCFA supplementation or modulation of tryptophan metabolism, are under investigation and may offer novel preventive or therapeutic options (Bourgonje et al., 2024; Stallmach et al., 2024). Thus, harnessing these microbial interventions could significantly shift the paradigm of CFS/ME prevention and management. Despite robust associations between the microbiome and CFS/ME, causality is difficult to establish due to confounding factors such as diet, medication use, and reduced physical activity. The heterogeneity of CFS/ME, encompassing genetic, environmental, and microbial influences, necessitates a multidisciplinary and individualized approach to therapy (Sampson, 2023; Uhde et al., 2023). There is a pressing need for standardized diagnostics and longitudinal studies to clarify the temporal relationship between microbiome changes and disease onset, as well as to identify reliable microbial or metabolic biomarkers for early detection and targeted intervention (Dai et al., 2024; Liu T. et al., 2024). Looking ahead, the MGBA represents a promising frontier in both the prevention and management of CFS/ME. Ongoing research into the specific microbial and metabolic pathways involved will be crucial for developing effective, evidence-based interventions that can improve the quality of life for individuals affected by this challenging disorder (Vogl et al., 2022; El-Sehrawy et al., 2025; Iqbal et al., 2025).

### 3.6 Stroke and post-stroke cognitive impairment (PSCI)

Post-stroke patients show gut dysbiosis with reduced microbial diversity, increased pro-inflammatory bacteria, and decreased levels of SCFA-producing bacteria such as *Faecalibacterium* (Yin et al., 2015). Changes are quite likely more related to increased systemic inflammation, BBB disruption, and cognitive outcome deterioration (Silva et al., 2020). Gut microbiota changes are linked to stroke outcomes, with mechanisms involving immune and inflammatory pathways; though interventions are in early stages, they represent a promising future direction.

#### 3.6.1 Background

Stroke is a leading cause of adult-onset neuropsychiatric and neurodegenerative disability, with substantial global prevalence and profound long-term consequences (Elendu et al., 2023). PSCI affects a significant proportion of survivors, manifesting as deficits in memory, executive function, and attention that markedly diminish quality of life and functional independence. In addition to well-established cerebrovascular and inflammatory mechanisms, emerging research highlights the gut microbiota's role in shaping stroke outcomes through the MGBA, influencing neuroinflammation, BBB integrity, and neuronal recovery (Wang et al., 2023a). This chapter will examine how stroke and PSCI are increasingly understood within the context of gut–brain interactions, and explore potential opportunities for intervention by targeting gut dysbiosis to improve neurological recovery and cognitive health.

#### 3.6.2 Gut–brain axis alterations in stroke

Growing evidence reveals that stroke not only disrupts CNS homeostasis but also profoundly alters the MGBA, leading to bidirectional interactions that can exacerbate neuroinflammation and hinder recovery. A systematic review of 18 studies highlighted the critical role of aging, inflammation, and shifts in gut microbiota, particularly involving Firmicutes, Bacteroidetes, SCFAs, and TMAO, in the pathogenesis and outcomes of ischemic stroke. This underscores how microbiome alterations may both predispose individuals to stroke and influence recovery (Lee Y. T. et al., 2021). These findings collectively suggest that restoring gut microbial balance could become an integral component of stroke prevention and recovery strategies. In a mouse model of ischemic stroke, Benakis et al. (2016) demonstrated that antibiotic-induced alterations of the gut microbiota significantly reduced brain infarct size. This neuroprotection was associated with increased intestinal regulatory T cells and reduced IL-17–producing γδ T cells, ultimately suppressing the trafficking of pro-inflammatory T cells from the gut to the leptomeninges after stroke. Their findings highlight a crucial MGBA in which intestinal immune modulation directly influences the severity of ischemic brain injury (Benakis et al., 2016). Singh et al. (2016) demonstrated in mouse models of middle cerebral artery occlusion that large ischemic strokes induce gut dysbiosis characterized by reduced diversity and Bacteroidetes overgrowth, leading to intestinal barrier dysfunction. Transplanting this dysbiotic microbiota into germ-free mice worsened stroke outcomes by driving proinflammatory T-cell responses and promoting lymphocyte migration to the injured brain. These findings underscore a bidirectional brain–gut–immune axis in stroke pathology (Singh et al., 2016). These mechanistic studies emphasize that targeting gut-driven immune responses could modulate neuroinflammation and improve stroke outcomes. Dysbiosis-driven disruptions of the MGBA have been implicated in both the development of common stroke risk factors, such as obesity, diabetes, and atherosclerosis, and in poorer recovery post-stroke, with age-related changes further compounding MGBA dysfunction (Honarpisheh et al., 2022). Recent research underscores that stroke-induced gut dysbiosis not only increases intestinal permeability and triggers systemic inflammation, but also facilitates translocation of microbes and immune cells across a compromised BBB, exacerbating neural injury; paradoxically, certain gut-derived metabolites may help limit post-stroke inflammation and support neurorepair (Hu et al., 2022). This highlights the complex bidirectional relationship between the gut microbiota and stroke pathology. Gut microbiota-derived metabolites, particularly SCFAs like butyrate and acetate, play critical roles in maintaining metabolic and immune homeostasis, with dysbiosis-linked alterations implicated in the pathophysiology of numerous neurological disorders including stroke, AD, and PD (Mirzaei et al., 2021). Emerging evidence suggests that modulation of SCFA production may offer therapeutic avenues for neuroinflammatory and neurodegenerative conditions. Together, these insights point toward microbiota-centered interventions—including strategies to enhance SCFA production—as promising approaches to mitigate neuroinflammation and promote brain recovery. In summary, growing evidence underscores that gut microbiota dysbiosis plays a pivotal role in both the development and outcome of ischemic stroke through immune, barrier, and metabolic pathways, suggesting that restoring gut microbial balance could become an essential strategy for stroke prevention and recovery.

#### 3.6.3 Mechanisms linking gut dysbiosis to PSCI

##### 3.6.3.1 Neuroinflammation

Gut dysbiosis and increased intestinal permeability following ischemic stroke (IS) have been linked to systemic endotoxemia and altered microbial metabolite signaling, potentially exacerbating neuroinflammation and injury (Zhang W. et al., 2023). Yin et al. (2015) demonstrated that patients with large-artery atherosclerotic ischemic stroke and transient ischemic attack (TIA) exhibited significant gut dysbiosis, characterized by increased opportunistic pathogens (e.g., *Enterobacter, Megasphaera, Desulfovibrio*) and reduced beneficial genera like *Bacteroides* and *Faecalibacterium*. Despite clear microbial shifts correlating with disease severity, these patients had lower plasma TMAO levels than asymptomatic controls, suggesting a complex interplay between gut microbiota composition and metabolic risk pathways. These findings highlight that gut microbial alterations, beyond just TMAO elevation, may contribute to cerebrovascular disease progression (Yin et al., 2015). Recent insights into the gut–brain and lung–brain axes highlight how abnormal intestinal microbiota, altered mucosal immunity, and even lung complications can exacerbate neuroinflammation and worsen outcomes after ischemic stroke (Xie et al., 2024). This underscores the complex bidirectional interplay between peripheral organs and the brain following stroke, mediated through immune and barrier mechanisms. For instance, it has been demonstrated in a mouse model of ischemic stroke that gut-derived bacteria can translocate to the lungs, leading to post-stroke infections. Using high-throughput 16S rRNA sequencing, Stanley et al. (2016) showed that stroke-induced gut barrier dysfunction preceded the dissemination of specific intestinal bacteria to peripheral tissues, highlighting a novel gut–lung axis in stroke pathology. These findings collectively emphasize that gut dysbiosis, increased intestinal permeability, and even gut–lung microbial translocation can amplify neuroinflammation and worsen ischemic stroke outcomes, underscoring the need to consider the gut–brain–lung axis as a potential target for improving stroke prognosis.

##### 3.6.3.2 Microbial metabolites

A substantial body of research highlights the role of gut microbiota and their metabolites, particularly SCFAs, in regulating metabolic, endocrine, and immune functions, with emerging evidence pointing to their involvement in neuro-immunoendocrine pathways through the MGBA (Silva et al., 2020). However, the precise mechanisms by which SCFAs influence brain physiology and behavior remain to be fully clarified, underscoring a promising avenue for developing novel CNS therapies. Longitudinal studies in spontaneously hypertensive stroke-prone have demonstrated that gut dysbiosis precedes the onset of hypertension and is marked by shifts in bacterial community structure and altered tryptophan-kynurenine metabolism, implicating microbial pathways in the early development of hypertension and related neurovascular complications (Shi et al., 2022). These findings suggest a potential mechanistic link between gut microbiota, systemic inflammation, and BBB vulnerability in stroke models.

##### 3.6.3.3 Immune modulation

Long-term use of proton pump inhibitors (PPI), while effective for acid-related disorders, has been shown to alter gut microbiota composition and increase the risk of infections such as SIBO and C. difficile, with emerging evidence suggesting that probiotic supplementation may help mitigate these adverse effects (Kiecka and Szczepanik, 2023). Emerging evidence suggests that microglia, as key neuroimmune modulators within the CNS, influence the incidence and progression of cardiovascular diseases, including hypertension, myocardial infarction, and ischemia/reperfusion injury, through mechanisms potentially linked to altered autonomic nervous system activity (Wang M. et al., 2022). These findings highlight microglia as promising therapeutic targets at the intersection of neuroinflammation and cardiovascular pathology. This highlights the need to consider gut microbial balance when evaluating prolonged PPI therapy. Recent experimental studies demonstrate that stabilizing mast cells with cromolyn after ischemic stroke reduces peripheral and central inflammation, preserves gut barrier integrity, mitigates dysbiosis, and improves functional outcomes, underscoring the pivotal role of gut-derived mast cells and histamine in post-stroke neuroinflammation (Conesa et al., 2023). These findings highlight an emerging gut–immune–brain pathway that may be targeted to improve stroke recovery.

#### 3.6.4 Interventions

##### 3.6.4.1 Probiotics

Gut dysbiosis has been increasingly recognized as a contributor to cardiovascular disease (CVD) pathogenesis through mechanisms involving systemic inflammation and metabolic disruption, with probiotics and prebiotics emerging as promising strategies to restore microbiota balance and improve cardiovascular markers such as LDL cholesterol and high-sensitivity C-reactive protein (hs-CRP; Wu and Chiou, 2021). This highlights the potential of microbiota-targeted interventions to mitigate CVD risk by maintaining immune and metabolic homeostasis. In a randomized, double-blind, placebo-controlled trial, daily supplementation with *Lactobacillus plantarum* ECGC 13110402 for 12 weeks in adults with moderate hypercholesterolemia resulted in a significant reduction in LDL cholesterol and hs-CRP compared to placebo, suggesting a direct benefit of probiotics on cardiovascular risk markers in humans (Costabile et al., 2017). Additionally, in a mouse model of ischemic stroke, administration of the prebiotic Puerariae Lobatae Radix-resistant starch (PLR-RS) improved neurological outcomes by restoring gut microbiota balance, enhancing gut barrier function, and increasing melatonin production. FMT from PLR-RS–treated mice to stroke mice reproduced these protective effects, highlighting a gut microbiota–melatonin axis as a therapeutic mechanism (Zhou et al., 2023). The gut microbiome, shaped by genetic, environmental, and lifestyle factors, plays a crucial role in immune, metabolic, and neural development, with dysbiosis increasingly implicated in the pathogenesis of neurological disorders such as stroke, PD, and AD (Sorboni et al., 2022). In a mouse model of AD, probiotic treatment with Lactobacillus and Bifidobacterium strains improved cognitive function and reduced amyloid-beta deposition, supporting a neuroprotective role of gut microbiota modulation (Bonfili et al., 2020). Growing evidence also supports microbiota-targeted interventions—including probiotics, prebiotics, synbiotics, and FMT—as promising therapeutic and diagnostic avenues in these conditions.

##### 3.6.4.2 Dietary interventions

Stroke not only causes direct brain injury but also induces systemic changes including gut dysbiosis and impaired intestinal barrier function, which can contribute to infection risk and influence stroke severity and recovery. Clinical and experimental studies reveal altered gut microbial diversity and metabolite profiles—such as elevated trimethylamine N-oxide and reduced short-chain fatty acids—highlighting the gut microbiota as a promising therapeutic target in stroke prevention and treatment (Peh et al., 2022). A cross-sectional analysis found that MeDi adherence was associated with increased gut microbial diversity and higher levels of short-chain fatty acids, metabolites linked to reduced inflammation and improved vascular health (De Filippis et al., 2016). Numerous studies have demonstrated that adherence to a MeDi, rich in diverse nutrients and phytochemicals, exerts anti-inflammatory, antioxidant, and anti-atherosclerotic effects, thereby lowering cardiovascular and cerebrovascular risk (Tuttolomondo et al., 2019). Growing evidence indicates that dietary herbs can modulate gut microbiota composition and generate bioactive metabolites that mitigate inflammation, oxidative stress, and apoptosis following stroke, underscoring a promising gut-mediated mechanism for herbal interventions (Li X. et al., 2025). This highlights the potential of gut microbiota–herb interactions in developing novel therapeutic and preventive strategies for stroke. In a rat model of ischemic stroke, administration of the herbal compound Salvia miltiorrhiza extract modulated gut microbiota composition, reduced infarct size, and decreased markers of inflammation and oxidative stress, indicating a gut-mediated neuroprotective effect (Wu J. et al., 2022). Polyphenols, widely consumed plant-derived compounds, have demonstrated protective effects on the cardiovascular and cerebrovascular systems, with emerging evidence from preclinical stroke models highlighting their neuroprotective potential, even when administered post-stroke (Parrella et al., 2020). These findings support the exploration of polyphenols not only in stroke prevention but also as adjuncts to enhance post-stroke recovery.

Animal models show microbiota modulation improves neuroinflammatory responses and functional outcomes. Human data are exploratory, but microbiota-targeted therapies like probiotics or dietary interventions could potentially support post-stroke recovery and cognitive health.

### 3.7 Radiation-induced neurotoxicity and MGBA disruption

#### 3.7.1 Background

Radiation therapy (RT) has redefined the management principles of several types of cancer at various stages, whether a primary neoplasm or metastatic lesion. Although it is effective in targeting cancer cells, it can alter many other tissues like CNS and GI tissue (Bai et al., 2021). The MGBA represents the mutual communication system between the GI tract and the CNS, using various mediators like neural and endocrine signals (Yang Q. et al., 2023). Recent evidence shows that gut microbiota plays a significant role in maintaining homeostasis and regulating the MGBA, which influences the neural and GI responses to RT (Bai et al., 2021). This review focuses on the relationship between gut microbiota, MGBA, and RT, highlighting the microbiota's potential to influence RT-induced neural and intestinal side effects.

#### 3.7.2 MGBA disruption by RT

Using RT in the abdomino-pelvic region can cause gut dysbiosis, due to the reduction of Firmicutes and Bacteroidetes species accompanied by the surge in Proteobacteria species. This imbalance is strongly linked to the breakdown of the mucosal barrier and high systemic LPS levels (Wang L. et al., 2021). The microbial translocation through the disrupted tight junctions of GI cells promotes systemic inflammation and can alter the BBB or cause other direct CNS effects via the Vagus nerve. Clinically, RT patients often report neuropsychological symptoms such as depression and fatigue, which may be mediated by microbiota-induced BBB dysfunction (Liu X. et al., 2024).

Venkidesh et al. (2023) did a pivotal experiment on rats using a 6 Gy pelvic irradiation causing a significant decrease in the microbiota's diversity, impaired GI morphology, neuroinflammation, and neuronal cell death. Moreover, they documented reduced hippocampal plasticity genes—alongside a change in the rodents behavior (Venkidesh et al., 2023). This study showed that pelvic irradiation can substantially promote pyknosis in the neurons of DG and CA2 regions of the hippocampus, which strongly supports that pelvic irradiation can cause memory and cognitive effects indirectly. Similarly, another study conducted by suggest that the use of antibiotics before cranial irradiation can be a contraindication. The study shows that applying cranial RT to mice after the administration of antibiotics reduced cytokine expression, especially IL-1β and TNF-α in the hippocampus and preserved cognitive functions (Luo et al., 2022). The authors believe that the transmigration of some of the SCFA bacteria, like the Firmicutes and Bacteroidetes species across the damaged tight junctions may exacerbate radiation-induced neurotoxicity (Luo et al., 2022).

#### 3.7.3 The role of microbiota in gut–brain communication

The gut microbiota produces key metabolites, such as SCFAs, neurotransmitter precursors, and tryptophan catabolites, which all influence the CNS. Butyrate and propionate, subtypes of SCFAs, are produced by anabolic commensals, such as *Faecelibacterium* and *Roseburia*, when they ferment dietary fiber (Wang L. et al., 2021). Butyrate has anti-inflammatory properties and serves as an epigenetic regulator by activating G-protein coupled receptors (GPR41 and GPR43) and inhibiting histone deacetylase activity (Du et al., 2024). Moreover, an experiment on rodents found that providing Butyrate supplementation effectively reduced hippocampus inflammation and preserved hippocampal neurogenesis following RT exposure (Lee et al., 2019a). Furthermore, species like *Bifidobacterium longum* have been shown to produce tryptophan metabolites that influence serotonergic signaling (Tian et al., 2019a), whereas *Lactobacillus* Spp. synthesize Gamma-aminobutyric acid (GABA), a crucial inhibitory neurotransmitter (Zheng et al., 2023). This suggests that any disruption of the microbial functions following RT may contribute to some neuropsychological symptoms like fatigue, depression, and cognitive dysfunction (Zhang Y. et al., 2023).

The MGBA can be influenced by many other microbial-derived metabolites, not only SCFAs (Deng et al., 2024). One example is indole-3-propionic acid (IPA), which is one of the iodole derivatives produced by the gut bacteria during tryptophan catabolism. IPA exhibits neuroprotective effects via activation of the aryl hydrocarbon receptor (AhR; Xiao et al., 2020). In preclinical models, IPA has been shown to enhance the expression of tight junction proteins in the BBB, reduce the severity of neuroinflammation, and preserve cognitive performance after CNS injury (Deng et al., 2024). In the radiation setting, any depletion in the IPA-producing species (e.g., *Clostridium* Spp. and *Peptostreptococcus*) strongly links to behavioral defects and the rise in pro-inflammatory cytokines in the CNS (Wlodarska et al., 2017; Xiao et al., 2020). Other microbial metabolites, like lactate and secondary bile, have the ability to modulate the neuroinflammatory response and influence synaptic plasticity, even though their roles in the post-radiation pathologies are still unspecified. Overall, microbially produced metabolites act as potent CNS regulators, either by modulating the vagus signaling and systemic immunity or by directly crossing the BBB (Deng et al., 2024). Understanding these complex interactions will open the gates to more promising therapeutic venues to enhance the overall quality of life of patients undergoing RT.

#### 3.7.4 Radiation-induced injuries and microbial mediation

**Figure 9:**
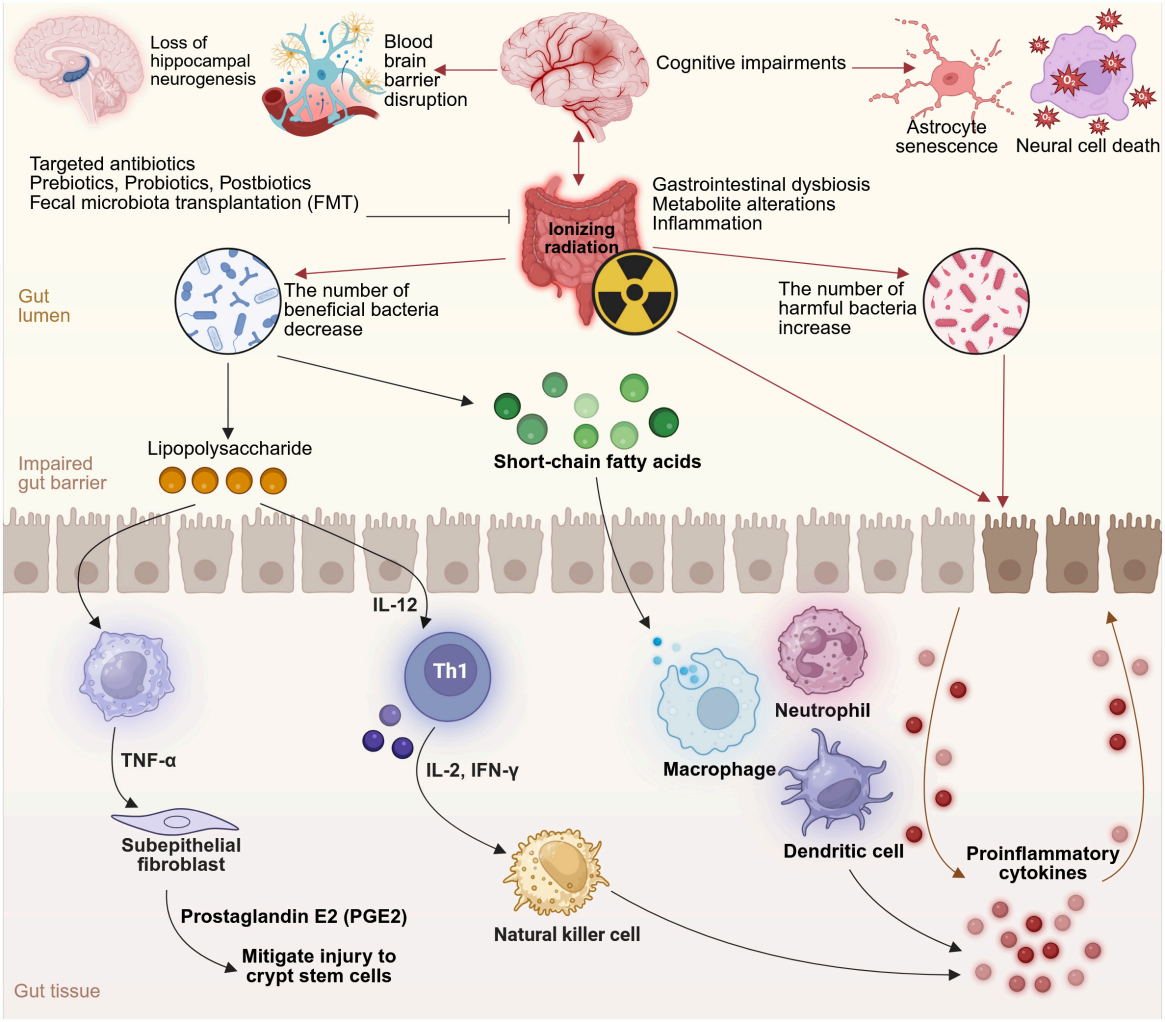
Ionizing radiation disrupts gut microbiota balance by decreasing beneficial bacteria and increasing harmful species, which damages the gut barrier and triggers inflammation and immune activation. This disturbance in the gut–brain axis leads to neural cell death and cognitive impairments. Interventions such as probiotics and FMT can help restore microbial balance and support gut and brain health. Created with BioRender.com.

Radiation-induced injuries initiate local and systemic responses, which are closely related to the gut microbiome's integrity. Wang Q. et al. (2025) found that the relation between gut microbiota and radiation-induced injuries could be direct and indirect. Direct interactions are related primarily to gut radiation and microbiota homeostasis, while indirect interactions are more related to other axes, like the MGBA. Pre-clinical studies done on C57BL/6J mice using total body irradiation (9 Gy) found that radiation reduced α-diversity of gut microbiota, while urolithin A (UroA), a gut microbial metabolite, helps in alleviating radiation-induced apoptosis in intestinal cells by suppressing P53 signaling pathway (Zhang et al., 2021c; Wang Q. et al., 2025). This explains that the direct relationship between the gut microbiota and radiation-induced injuries is based on microbial homeostasis (Figure 9). Also, clinical studies done on patients with cervical cancer using pelvic radiation explained that Firmicutes-Proteobacteria ratio (F/P) reflects the severity of radiation toxicity, where it showed that patients with chronic radiation enteritis exhibited a reduction in Firmicutes and an increase in Proteobacteria. Moreover, the same study found that patients with severe enteritis showed high abundance of *Shigella* and *Lachnospiraceae Clostridium*. This supports a direct interaction, although their underlying mechanisms are still in the early stages of investigation and further studies with larger sample sizes or validation are required (Wang Q. et al., 2025).

Several pre-clinical studies illustrated the indirect interactions between gut microbiota and radiation injuries, especially brain injuries via the MGBA. A preclinical study used C57BL/6 J mice exposed to head radiation found that the predominet components of the subjects' gut microbiota following radiation were *Bacteroidales, Muribaculaceae, Erysipelotrichales*, and *Ruminococcus*. Gut microbiota-derived metabolites, like SCFAs, were shown to play a pivotal role in the MGBA by elevating pro-inflammatory cytokines in the subjects' blood, worsening radiation-induced brain injury (Luo et al., 2022; Hu et al., 2023; Wang Q. et al., 2025). Another pre-clinical study done on C57BL/6J mice using abdominal irradiation (10 Gy) found that the expression level of miR-34a-5p in the small intestine tissue and peripheral blood significantly increased, which mediated distant cognition impairment. Cognitive functions can be maintained by intravenous administration of miR-34a-5p antagomic immediately post-radiation, which blocks miR-34a-5p upregulation in peripheral blood, resulting in restoring the hippocampal expression of BDNF (Cui et al., 2017a; Wang Q. et al., 2025).

#### 3.7.5 Host-microbiota immune crosstalk during radiation exposure

The communication between microbial dynamics and the host's immune system play a major role in determining the strength of the systemic inflammation during RT. Ionizing radiation has been proven to increase the expression of TLR on intestinal epithelial and immune cells, making them more sensitive to microbial ligands like LPS ad flagellins. In radiation settings, upregulated expression of TLR4 enhances the sensitivity and response to Proteobacteria-derived ligands, amplifying downstream NF-κB signaling and cytokine secretion (Lu et al., 2020). Recent experiments showed a surge in IL-6, IL-1β, and TNF-α levels within irradiated mice serum, which is revoked in MyD88-deficient animals. This highlights the importance of the innate immune system responses in driving systemic inflammation following RT. Simultaneously, the upregulation of co-stimulatory molecules (CD80/86) of dendritic cells conditioned in the irradiated gut mucosa suggests a pro-inflammatory antigen-presenting phenotype. The previous interactions contribute to the activation of the peripheral immune system and primes CNS microglia, creating a neuroinflammatory environment that aggravate behavioral deficits (Manda et al., 2012; Wolff et al., 2021). Notably, certain commensals, like *Clostridium* cluster XIVa and segmented filamentous bacteria, have the ability to push the host immune system toward regulatory responses via regulatory Treg expansion and IL-10 production (Atarashi et al., 2011). Deficiency in these taxa following RT is significantly connected with prolong tissue injury and impaired mucosal tolerance (Touchefeu et al., 2014). These findings show that microbial-immune interactions have dual role, mediators and potential modulators of radiation toxicity, along the MGBA.

#### 3.7.6 Preventive measures and microbiota-preserving strategies

Given the importance of gut microbiota and its mitigate radiation-induced toxicity., implementing preventative strategies focusing on preserving the microbial diversity and barrier quality is crucial. In murine models, FMT has shown therapeutic benefits by restoring Firmicutes species and attenuating systemic inflammation post-RT. Timing is pivotal; administering FMT pre-RT has been shown to provide more benefits compared to post-RT (Cui et al., 2017b). FMT is beginning to move toward clinical application. Pilot clinical trials found that FMT might be a safe and effective approach for patients that suffer from post-RT enteritis, although it can alter the patients' gut microbiota composition (Ding et al., 2020). Dietary interventions enrich with microbial substrates, such as polyphenols and dietary fiber, may enhance endogenous synthesis of beneficial metabolites like SCFAs and IPA. Recent studies done on animals found that high fiber diets helped in mitigating hippocampal inflammation following abdominal radiation, while preserving goblet cell integrity and tight junction protein expression (Church et al., 2023; Lu et al., 2024). Stress management techniques and exercising regularly have been shown to promote microbial diversity and cognitive resilience, potentially leading to improved neurocognitive outcomes following RT (Matei et al., 2023). Limiting non-indicated use of antibiotics during RT is crucial to prevent excessive loss of microbiota (Poonacha et al., 2022). Personalized multimodal strategies—integrating lifestyle, nutrition, and targeted therapies—require further development and validation in clinical trials.

#### 3.7.7 Scientific gaps

Although pre-clinical studies are promising, several gaps remain in understanding the exact microbial species and metabolites responsible for reducing RT side effects on the gut and brain. Many clinical studies fail to incorporate longitudinal assessments of both microbiome and neurocognitive data. The limited incorporation of advanced ‘omics platforms, like metabolomics and metatranscriptomics has yet to reliably identify biomarkers of RT response and toxicity (Liu et al., 2021). Fourth industrial revolution tools, such as artificial intelligence and machine learning, are crucial in linking all the variables and further understand their mechanism of action. This is accomplished by developing generalized robust models, that minimize bias, along with explained machine learning techniques for multifactorial longitudinal data to analyze in more accurate manner and ensure future research success. Using these tools will aid the precision of radiation therapy, minimizing the risks of radiation-induced toxicities and injuries, and making medical care more personalized (Herrera-Quintana et al., 2024). Moreover, most of the existing preclinical studies have relied on male rodents, neglecting sex-specific variants in gut microbiota compositions and neuroinflammatory responses (Chakraborty et al., 2025). The field currently lacks the application of standardized approaches for microbiota modulation, like FMT and precision nutrition, which further hinder reproducibility and clinical application (Lu et al., 2024). Subsequent research must further clarify the role of the MGBA axis across varying malignancies, radiation site, and dose parameters (Li et al., 2022). Cross-disciplinary collaboration will be the key in achieving applicable realistic progress in personalized radiation oncology intervention (Yi et al., 2023).

The MGBA represents a dynamic modulator, where microbiota can influence the host's pathophysiological responses to RT. Preclinical evidence shows the significance of microbial metabolites and immune signaling in driving these effects, and recent clinical studies further supports these findings (Touchefeu et al., 2014; Wang et al., 2015). Implementing strategies that targets the MGBA axis can significantly reduce RT-associated morbidities. A clear Interdisciplinary approach containing microbiology, neuroimmunology, and behavioral sciences is needed to turn these theoretical data into realistic therapies. As we refine our knowledge about microbiota-RT interactions, microbiome modulation strategies are poised to play a key role in personalized oncology radiation treatments.

### 3.8 Xenobiotics and neurotoxicity

#### 3.8.1 Background

**Figure 10:**
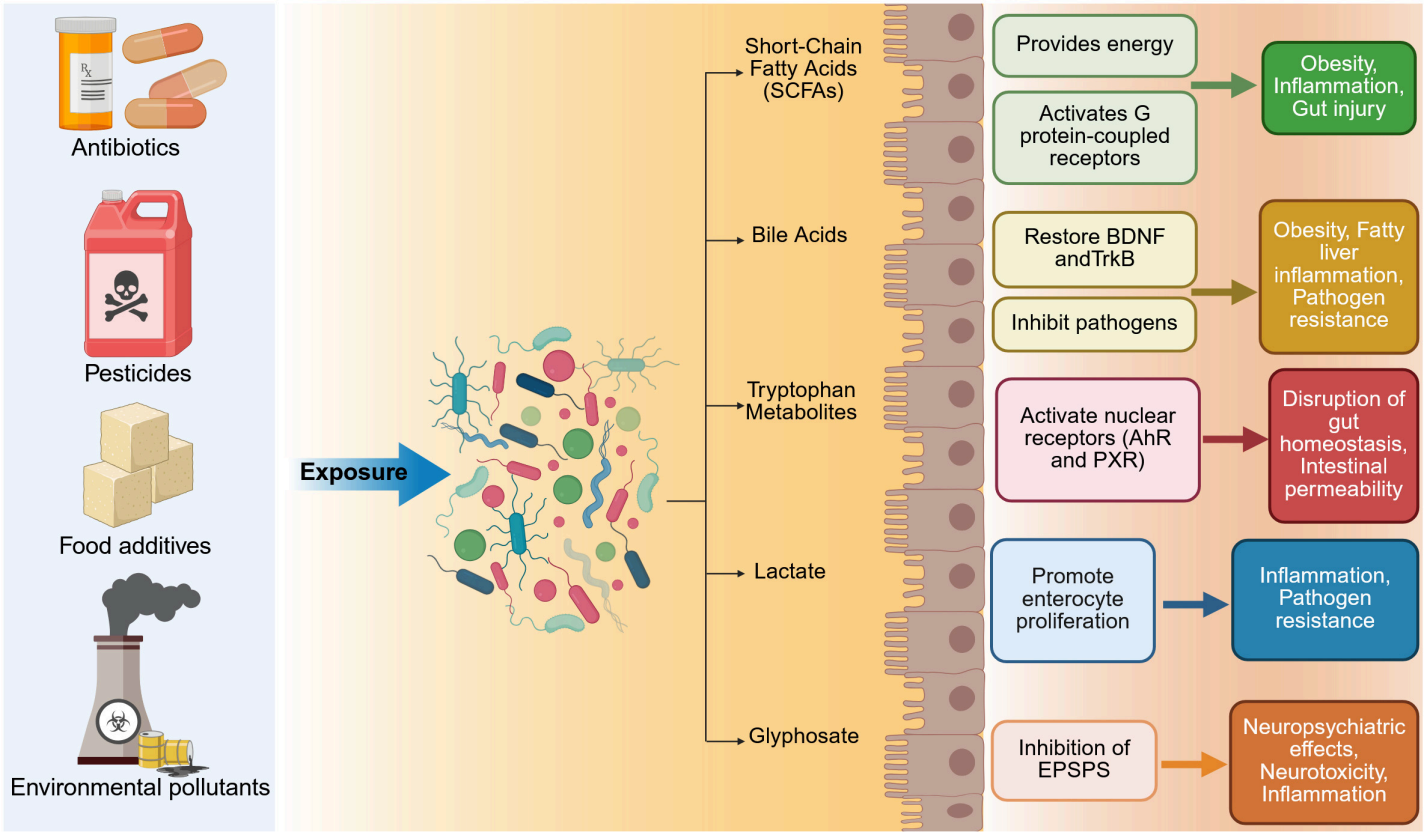
A diagram showing how xenobiotics may disrupt gut microbiota and contribute to adverse health outcomes. Created with BioRender.com.

Xenobiotics are chemical substances foreign to the human body, commonly found in pharmaceuticals, food additives, pesticides and environmental pollutants—making exposure unavoidable (Jin et al., 2023). These compounds enter via ingestion, inhalation or dermal absorption and primarily accumulate in the GI tract (Hawkins et al., 2020). The gut microbiota, comprising diverse bacterial species, plays a central role in metabolizing xenobiotics through detoxification, bio activation or conversion into excretable forms (Catron et al., 2019). Given the vast number and variety of xenobiotics encountered by the gut microbiota—over 25,000 compounds, understanding these interactions is critical (Lindell et al., 2022). Xenobiotics can reshape microbial communities, alter spatial organization, and disrupt host–microbiota signaling (Sutherland et al., 2020; Figure 10). These disruptions have been found to impair communication in the MGBA, a pathway increasingly linked to psychiatric symptoms (De Filippis et al., 2024).

Although emerging evidence links xenobiotics, such as antibiotics (most commonly prescribed pharmaceuticals), glyphosate (most widely used herbicide), and microplastics (MPs)—(ubiquitous emerging environmental contaminant) with anxiety and depression, studies remain limited and mechanisms of action are poorly understood (Zhang et al., 2021a; Hsiao et al., 2023; Luo and Lin, 2025). Given that these mental health conditions are projected to become the leading contributor to the global disease burden by 2030 (Liu J. et al., 2024), understanding how xenobiotics disrupt gut–brain communication is urgently needed. This review examines how these three major xenobiotic classes disrupt gut–brain axis communication, potentially contributing to anxiety and depression-like symptoms.

#### 3.8.2 Microplastics

Plastic manufacturing has undergone a rapid expansion since 1950. In 2022, global production reached 400 million tons, and it is expected to double by 2050 (Houssini et al., 2025). These materials degrade slowly, fragmenting into MPs (<5 mm) and nanoplastics (NPs; 1–100 nm), which have emerged as widespread environmental pollutants and a potential threat to human health (Zheng et al., 2024). MPs/NPs are either directly used in cosmetics, detergents, lotions, shampoos, synthetic textiles, and industrial processes, or formed secondarily through the degradation of larger plastic items such as packaging or tire (Zheng et al., 2024). Common polymers include polyethylene (PE), polypropylene (PP), polystyrene (PS), and polyvinyl chloride, often combined with additives such as plasticisers or flame retardants (Zheng et al., 2024). MPs/NPs have been detected across aquatic species including molluscs, fish, and crustaceans, raising concern about bioaccumulation in humans, with estimated intake ranging from 0.1 to 5 g/week via contaminated food, water and air (Li G. et al., 2024; Zheng et al., 2024). This exposure concern has been validated by direct tissue analysis. Postmortem human study demonstrated MPs/NPs were 7–30 times higher in frontal cortex than kidney or liver, with 75% of particles identified as PE (Nihart et al., 2025). Frontal cortex concentrations increased by 50% from 2016 to 2024, with dementia cases exhibiting higher accumulation than controls (Nihart et al., 2025).

Furthermore, a preclinical study demonstrated that circulating MPs may impair brain function via immune-mediated vascular occlusion (Huang et al., 2025). Following MP phagocytosis, immune cells obstructed cortical capillaries, producing neurological deficits that persisted for 4 weeks (Huang et al., 2025). The authors demonstrated that larger particles (5 μm) caused more severe obstruction than smaller ones (2 μm, 80 nm), suggesting size-dependent neurovascular toxicity. Interestingly, while maternal PE MPs/NPs exposure increased umbilical flow by 43% in mice, it did not affect fetal growth or brain circulation—suggesting effective compensatory mechanisms (Hanrahan et al., 2024). Collectively, these studies suggest MPs/NPs potentially exhibiting neurotoxic effects on cerebral tissue and vascular integrity, while negative effects during fetal exposure may be mitigated by placental adaptation.

Human exposure to microplastics can occur via respiratory inhalation, transdermal absorption and oral ingestion via food and plastic packaging (Wu P. et al., 2022) with the gut being a primary site of accumulation (Sun et al., 2025). MPs were found in every stomach examined at autopsy, averaging 9.4 particles per person, with an estimated daily intake of 32.2 particles (Özsoy et al., 2024). Recent preclinical studies found that gut microbiota participates in MPs and NPs induced anxiety and depression-like behaviors by modulating the gut–brain axis. MPs disrupt the Firmicutes/Bacteroidetes (F/B) ratio—a key marker of microbial balance in mice (Chen et al., 2023; Yang J.-Z. et al., 2023; Wang J. et al., 2024) and zebrafish (Qian et al., 2025) suggesting a conserved disruption of dominant phyla across species in response to MPs exposure. This was often accompanied by enrichment of pro-inflammatory Proteobacteria and depletion of protective phyla such as Verrucomicrobia, indicating a shift toward inflammation and barrier dysfunction (Stolfi et al., 2022). Furthermore, MPs induce gut dysbiosis by decreasing probiotic bacteria and increasing pro-inflammatory. In male mice even low-dose exposure to PS-MPs and PS-NPs (0.5 mg/day for 60 days) reduced *Lactobacillus*, a genus important for epithelial maintenance (Chen et al., 2023). Similar reductions followed in male mice study exposed to low-density polyethylene MPs (LDPE-MPs) and oxidized low-density polyethylene MPs (Ox-LDPE-MPs) for 28 days (Wang et al., 2023b; Wang J. et al., 2024) suggesting microbial vulnerability across polymer types. Similarly, SCFA-producing genera were consistently depleted in male rodent models exposed to PS-MPs and PS-NPs including *Faecalibaculum* and *Akkermansia* (Yang J.-Z. et al., 2023)*, Oscillibacter* and *Ruminococcus* (Jiang et al., 2023), and *Lachno Clostridium* (Chen et al., 2023). These losses occurred irrespective of dose, duration, or particle size. Moreover, an overgrowth of pro-inflammatory gut bacteria was shown across rodent models and doses. This included *Desulfovibrio* (Chen et al., 2023), *Mucispirillum, Helicobacter, Paraprevotella*, and *Tuzzerella* (Yang J.-Z. et al., 2023). Moreover, gut barrier dysfunction was consistently observed in rodent models following microplastics exposure, marked by reduced expression of tight junction proteins—ZO-1, Occludin, Claudin-1, and Claudin-5 (Jiang et al., 2023; Yang J.-Z. et al., 2023; Wang J. et al., 2024). This epithelial disruption was linked to elevated serum LPS, confirming increased intestinal permeability and microbial translocation. Increased levels of IL-1β, IL-6, and TNF-α further demonstrated systemic immune activation (Yang J.-Z. et al., 2023; Wang J. et al., 2024). Interestingly, Ox-LDPE-MPs showed greater inflammatory responses and intestinal damage compared to LDPE-MPs which was hypothesized to result from their easier cellular accumulation (Wang et al., 2023b).

Furthermore, peripheral immune changes were associated with neuroinflammatory responses in mood-related brain regions. Hippocampal TLR4/MyD88/NF-κB signaling was activated (Yang J.-Z. et al., 2023) the cAMP/PKA/p-CREB pathway was altered in the amygdala alongside neuronal apoptosis (Jiang et al., 2023), and cortical cytokines—including IL-1β, IL-6, and TNF-α were elevated (Wang J. et al., 2024), each coinciding with anxiety- and depression-like behaviors. Exposure to MPs consistently disrupted monoaminergic and cholinergic signaling. In rats, long-term low-dose PS-NPs exposure reduced amygdalar metabolites-N-acetylserotonin, N-acetyl-L-tyrosine, and 4-aminobutyric acid, suggesting impaired neurotransmitter metabolism (Jiang et al., 2023). In mice, 28-day exposure to LDPE and Ox-LDPE MPs reduced acetylcholine levels in the cortex and hippocampus (Wang J. et al., 2024). In zebrafish, 90-day exposure to Polyactic Acid (PLA) and Aged Polyactic Acid (APLA) MPs (1–20 mg/L) suppressed 5-HT and DA, decreased acetylcholinesterase activity in the brain and gut and downregulated BDNF/TrkB signaling, coinciding with neuronal apoptosis. Interestingly, these effects were more pronounced in the high-dose APLA group compared to PLA, suggesting dose- and type-dependent neurotoxicity (Qian et al., 2025). Behavioral alterations from MPs exposure demonstrated clear dose-response and particle-specific patterns (Jiang et al., 2023) reported no alteration in neuropsychiatric symptoms at 12 weeks but observed clear effects after 24 weeks of exposure, supporting cumulative neurotoxicity. There were greater impairments with higher-dose APLA vs. PLA, suggesting dose- and polymer-specific toxic effects on neuropsychiatric symptoms (Qian et al., 2025). Furthermore, Ox-LDPE caused more pronounced anxiety-like behaviors compared to LDPE, with oxidized particles producing more severe behavioral impairments (Wang J. et al., 2024). MPs caused more pronounced anxiety-like behaviors compared to NPs after 30 days of exposure, hypothesizing that NPs are more easily absorbed and removed due to their smaller size, resulting in less neurotoxicity than MPs (Chen et al., 2023). The behavioral toxicity threshold of MPs remains to be defined. Future studies should investigate the effects across polymer types, doses and exposure durations. Several interventions have reversed MPs-induced neurotoxicity by targeting the gut microbiota, supporting a causal role of the MGBA (Yang J.-Z. et al., 2023). A treatment with epigallocatechin gallate (EGCG), a polyphenol from green tea, restored microbial composition, preserved tight junction expression, and reduced systemic inflammation (Yang J.-Z. et al., 2023). Moreover, rats treated with 1% neohesperidin dihydrochalcone for 1 month showed reduced amygdala neuroinflammation via suppression of cAMP/PKA pathway activity, restoration of cortical 5-HT, DA, and NE levels, and reversal of anxiety- and depression-like behaviors (Jiang et al., 2023). Similarly, bile acid treatment rescued microbial diversity and restored 5-HT, DA, acetylcholine, BDNF, and tropomyosin receptor kinase B levels in both brain and gut of zebrafish, alleviating behavioral symptoms (Qian et al., 2025). Moreover, supplementation with *Lactobacillus plantarum* (DP189) and GOS restored gut microbial composition, improved gut and BBB integrity, reduced cortical inflammation and elevated acetylcholinesterase activity, reversing anxiety and depression-like behaviors in mice (Wang J. et al., 2024).

While human studies remain scarce, emerging *in vitro* models simulating human gut conditions reveal variable microbiota responses to MPs, ranging from dysbiosis and inflammatory metabolite production to probiotic enrichment and metabolic adaptation (Fournier et al., 2023; Jiménez-Arroyo et al., 2023). In the M-ARCOL model (Mucosal Artificial Colon, an advanced *in vitro* system using microbiota from healthy donors), daily exposure to PE—MPs for 14 days increased pathobiont *Desulfovibrionaceae* (Fournier et al., 2023). Elevated levels may contribute to the development of depression by promoting hydrogen sulfide–mediated inflammation (Yang et al., 2017). Furthermore, donor-dependent rises in *Enterobacteriaceae*, alongside reductions in protective taxa *Christensenellaceae* and *Akkermansiaceae* were found (Fournier et al., 2023). *Christensenellaceae* exhibits a capacity to influence the HPA axis, a central regulator of the body's stress response (Agusti et al., 2024) and depleted levels have been found in individuals with MDD, supporting their proposed role as protective taxa within the MGBA (Liu R. T. et al., 2020).

Moreover, skatole, a tryptophan-derived metabolite typically low in healthy individuals, was also elevated, suggesting disruption of gut homeostasis (Fournier et al., 2023). However, biodegradable plastics show contrasting effects. Interestingly, certain MPs may be metabolically processed by the human gut microbiota without causing detrimental alterations. In the SIMGI^®^ model (an *in vitro* simulator of human digestion), a single dose of 0.166 g of PLA—a widely used biodegradable plastic was followed by 72-h colonic fermentation. An increase in probiotic *Bifidobacterium* was detected in all donors (Jiménez-Arroyo et al., 2023). During this process, PLA particles were colonized by gut microbes, forming surface biofilms and increasing pullulanase activity—an enzyme that degrades branched polysaccharides—the authors suggested this may have broken down PLA into smaller carbon-rich molecules, potentially supporting *Bifidobacterium* growth, without triggering pathogenic overgrowth or inflammatory responses (Jiménez-Arroyo et al., 2023). These findings were challenged by a more recent *in vitro* study, using fecal microbiota from healthy donors (Peng et al., 2024). Biodegradable MPs such as PLA and Poly(ε-caprolactone) underwent degradation and oligomerization while significantly depleting beneficial bacteria including *Bifidobacterium, Lactobacillus, Faecalibacterium, Blautia*, and *Ruminococcus* (Peng et al., 2024). These play a significant role in neurotransmitter production, inflammation control and barrier regulation and their depletion is frequently observed in individuals with depression and anxiety further suggesting their relevance to mental health (Borkent et al., 2022; Xiong R.-G. et al., 2023). Moreover, biodegradable MPs increased potentially harmful *Megamonas* and *Prevotella* with SCFA production impairment, authors hypothesizing that biodegradable plastics may cause greater harm than conventional plastics because their degradation creates oligomers—smaller breakdown products that can more easily penetrate biological barriers and further exhibit detrimental effects on gut microbiota (Peng et al., 2024). However, small donor sample sizes limit generalizability, as they may not capture donor-dependent shifts or reflect broader diversity in age, diet, or health. Moreover, these models used adult microbiota, despite evidence that infants are found to contain higher levels of MPs in fecal samples (Zhang et al., 2021b). Future studies should include larger, diverse cohorts and age-relevant microbiota.

#### 3.8.3 Glyphosate

Glyphosate is a widely used herbicide acting via inhibition of the enzyme 5-enolpyruvylshikimate-3-phosphate synthase, which blocks the shikimate pathway responsible for synthesizing aromatic amino acids including phenylalanine, tyrosine and tryptophan (Chiantia et al., 2025). These are known precursors to neurotransmitters that modulate mood, behavior and cognition. The shikimate pathway is absent in mammals; therefore, glyphosate has been considered safe for human health, however, this pathway is present in plants and many microorganisms, including members of the gut microbiota (Mesnage et al., 2021). As some gut bacteria rely on the shikimate pathway for nutrient synthesis or metabolic cross-feeding, glyphosate exposure may alter microbial composition, as 12–26% of human gut bacterial species may be sensitive to glyphosate (Leino et al., 2021; Puigbò et al., 2022). Therefore, GBA may be proposed as a potential mediator of glyphosate-associated neuropsychiatric effects in humans (Lehman et al., 2023). Although glyphosate has been classified as a potentially carcinogenic substance (Guyton et al., 2015) and has been linked to psychiatric symptoms and neurotoxicity (Costas-Ferreira et al., 2022) the debate on glyphosate's detrimental effects on human health continuously persists in the scientific community with scarce number of studies and inconsistent findings.

Preclinical studies found that glyphosate exposure can induce anxiety and depression-like behaviors by disrupting gut microbiome composition, impairing neurotransmitter levels and increasing neuroinflammation (Aitbali et al., 2018). Adult mice exposed to low-dose glyphosate exposure at environmentally relevant levels (10 μg/ml) for 90 days showed reduced levels of beneficial bacteria *Lactobacillus* and *Bifidobacterium* (Lehman et al., 2023). These bacteria are found to exhibit a greater sensitivity to glyphosate exposure (Shehata et al., 2013) and are often found at reduced levels in individuals with MDD and may play a role in the development of mood disorders (Aizawa et al., 2019). Moreover, detrimental effects of glyphosate on gut–brain axis exhibit transgenerational effects. Reduced beneficial bacteria *Akkermansia* and *ParaBacteroides* and overgrowth of Alistipes and Blautia were found in offspring of mice exposed to glyphosate during pregnancy and lactation (Buchenauer et al., 2023). This microbial shift disrupts multiple gut–brain pathways simultaneously: these shifts coincided with gut barrier disruption (elevated serum endotoxin), triggering elevated hippocampal cytokines (IL-6, TNF-α), which in turn suppress Tph2 expression and induce Tph2 hypermethylation (suggesting neuroinflammation and serotonergic suppression), ultimately manifesting as anxiety and depression-like behaviors (Buchenauer et al., 2023). These effects were dose-dependent, emerging only at 50 mg/kg/day—a level still classified as safe in animals by regulatory standards. Furthermore, the effects were sex-dependant—recorded in female offspring only. The selective vulnerability in females led the authors to hypothesize a role for estrogen-linked mechanisms, although this was not tested. Future studies should determine whether hormonal factors mediate this sex-specific sensitivity (Buchenauer et al., 2023). Moreover, exposing mice to a low dose which reflects a typical American diet (0.01 mg/kg/day), caused gut microbiota alterations across two generations-consistent with earlier findings, *Akkermansia muciniphila* was depleted, while *ParaBacteroides distasonis* and *Christensenellaceae* were elevated in the second generation (Barnett et al., 2024). Interestingly, *ParaBacteroides* responses appear to vary between studies, as Buchenauer et al. (2023) observed *ParaBacteroides* depletion rather than enrichment, suggesting exposure timing or generation-dependent effects. Whilst these microbial shifts coincided with gut barrier disruption (loss of ZO-2 tight junction protein and goblet cell depletion), colonic immune activation (elevated pro-inflammatory cytokines), and altered levels of gut-derived metabolites relevant to neuropsychiatric function (reduced GLP-1 and serum kynurenine), affective symptoms were not detected at the time of testing (Barnett et al., 2024), possibly due to delayed onset of behavioral abnormalities (Del Castilo et al., 2022). Future studies should employ longitudinal study designs to clarify the delayed effects of glyphosate on gut–brain interactions.

#### 3.8.4 Antibiotics

Antibiotics are commonly prescribed medications in clinical settings and may represent one of the most powerful disruptors of the MGBA (Hao et al., 2021). Antibiotic-induced depression followed by an attempt to suicide was first reported in 2010 (Ahmed et al., 2011). Since then, large human cohort studies have linked antibiotic exposure to increased risk of depression and anxiety, with disruption of the MGBA proposed as a key mechanism (Gudnadottir et al., 2024; Lee et al., 2024). This proposed mechanism has been directly validated in animal models where the surgically severed vagus nerve attenuated anxiety- and depression-like behaviors in mice after oral antibiotic administration, providing direct evidence for neuropsychiatric effects induced by antibiotics through the gut–brain axis (Joo et al., 2023). Preclinical studies have demonstrated that antibiotic treatment induces gut dysbiosis-marked by reduced alpha diversity, a decreased Firmicutes/Bacteroidetes ratio, depletion of beneficial genera, and enrichment of pro-inflammatory taxa (Tejkalová et al., 2023; Thabet et al., 2024). These microbial shifts trigger cascading gut–brain disruption by impairing neurotransmitter signaling, suppressing SCFA production, weakening epithelial barrier integrity, and activating neuroimmune pathways, ultimately resulting in anxiety- and depression-like behaviors in rodent models (Tejkalová et al., 2023; Li N. et al., 2024; Bibi et al., 2025). In mice treated with amoxicillin or ciprofloxacin, depletion of *Lactobacillus reuteri* (Firmicutes) led to reduced brain and serum GABA, downregulation of GABA-A receptors, and diminished 5-HT and DA, neurochemical changes that coincided with anxiety- and depression-like behaviors (Bibi et al., 2025). Intergenerational exposure to vancomycin and streptomycin resulted in progressive microbial loss, with the most pronounced shifts observed in third-generation offspring (Li N. et al., 2024). Depletion of beneficial taxa such as *Odoribacter* (Bacteroidetes) and *Lachno Clostridium* (Firmicutes), along with increased abundance of pro-inflammatory taxa including *Ileibacterium* and *Olsenella*, was associated with reduced SCFA gene expression, downregulation of tight junction proteins (occludin and claudin-1), and microglial activation in the amygdala and arcuate nucleus, indicating gut barrier disruption and neuroinflammation that coincided with anxiety-like behavior. Soil microbiota restoration reversed anxiety-like behavior, attenuated microgliosis, and restored gut microbial composition in affected mice (Li N. et al., 2024). However, findings remain inconsistent across studies. A recent two-hit rat model study found that antibiotic-induced dysbiosis, including increased *Proteobacteria* and reduced *Bacteroidetes*, failed to amplify anxiety-like behaviors in immune-primed rodents, contradicting prior evidence and highlighting the complexity of gut–brain interactions (Tejkalová et al., 2023). Future studies should clarify the role of immune priming in modulating behavioral responses to antibiotic-induced gut–brain effects.

## 4 Conclusion and future perspectives

Increasing information has pointed out the importance of MGBA in all stages of neurodevelopment and progression in a wide variety of neuropsychotic diseases and neurodegenerative disorders, as well as their treatment. Dysbiosis is common among conditions like AD, PD, MS, MDD, BD, anxiety, PTSD, and stroke. Gut microbiota disruption has the potential to provoke a number of processes, including neuroinflammation, BBB dysfunction, dysregulation in immunity, and new patterns in neurotransmitter metabolism. These pathologies are mediated by microbial metabolites, such as SCFAs, microbial endotoxins, and others, that neurologically act on human brain health directly and indirectly. This review demonstrates various roles of MGBA in different disease process mechanisms. For example, in both neurodegenerative diseases like Alzheimer's and Parkinson's, the presence of fewer bacteria generating SCFA and the increased density of proinflammatory microbes lead to neuroinflammation and cognitive decline. In affective disorders such as depression, BD, and PTSD, alterations in serotonergic, dopaminergic, and GABAergic signaling often regulated by microbial misbalanced signaling render gut microbiome as strongly connected to emotional and behavioral aptness. Gut-derived immune activation exacerbates central pathology in stroke and in autoimmune diseases like MS, while there is evidence that dietary interventions and probiotics can also attenuate disease severity and improve functional outcome. While pre- and probiotics are currently limited to being functional foods for promoting health, the future of live biotherapeutic products (LBPs) holds much promise for the prevention and management of neuropsychiatric and neurodegenerative disorders. The rigorous characterization, safety evaluation, and standardization of pre- and probiotics are instrumental in developing them as LBPs such that therapeutic effects can be reliably obtained. This approach could offer precision in modulating the specific microbial taxa or metabolic activities involved in the disease processes, paving the way for personalized therapeutic strategies. However, these are some of the converging themes despite the nuances posed by each specific disease. First, it would appear that gut dysbiosis is a cause and consequence of CNS pathology and hence creates self-propelling loops among inflammation, immune activation, and barrier dysfunction. Second, the MGBA is bidirectional and the gut highly modulates, as much as the brain influences, state of gut physiology. Thirdly, among interventions that target the microbiome are probiotics, prebiotics, SCFA supplementation, dietary modifications (e.g., Mediterranean diets or those rich in fibers), FMT, and emerging LBPs, all of which combine toward what could be considered a promising frontier in therapeutic potential. Still in its infancy, preclinical and clinical investigations have indicated that these therapies can change the course of diseases, relieve or ameliorate symptoms, or even change treatment response to a certain extent. Going forward, future research should address the following gaps that need to be filled. First, they need to be well-detailed mechanistic studies that disentangle causal relationships between specific microbes and neurological outcomes rather than mere correlational evidence that would lead to therapeutic targets. Additionally, appropriate human longitudinal studies and standardized protocols for measuring durability and safety of microbiota-based interventions among different patient populations will be important. Finally, it is envisaged that personalized approaches integrating individual microbiome profiles, genetic background, and lifestyle factors will be critical in using this knowledge to develop effective, tailored therapies. Such LBPs may also help transform microbiome-targeted therapies from adjuncts to scientifically validated, medical-grade modalities in preventing or slowing neurodegenerative and neuropsychiatric disease progression. The gut microbiota is, thus, modifiable as well as a clinically actionable component. In moving toward a systems level understanding of the MGBA and leveraging advances in microbiome science-the LBPs-it is all possible to usher in a new era of preventive and therapeutic agendas toward complex neuropsychiatric and neurodegenerative disorders.

## References

[B1] Abautret-Daly Á. Dempsey E. Parra-Blanco A. Medina C. Harkin A. (2018). Gut–brain actions underlying comorbid anxiety and depression associated with inflammatory bowel disease. Acta Neuropsychiatr. 30, 275–296. 10.1017/neu.2017.3 28270247

[B2] Aguirre M. Eck A. Koenen M. E. Savelkoul P. H. M. Budding A. E. Venema K. (2016). Diet drives quick changes in the metabolic activity and composition of human gut microbiota in a validated *in vitro* gut model. Res. Microbiol. 167, 114–125. 10.1016/j.resmic.2015.09.006 26499094

[B3] Agusti A. Molina-Mendoza Gv. Tamayo M. Rossini V. Cenit Mc. Frances-Cuesta C.. (2024). Christensenella minuta mitigates behavioral and cardiometabolic hallmarks of social defeat stress. Biomed. Pharmacother. 180:117377. 10.1016/j.biopha.2024.117377 39316970

[B4] Ahmed A. I. A. Van Der Heijden F. M. M. A. Van Den Berkmortel H. Kramers K. (2011). A man who wanted to commit suicide by hanging himself: an adverse effect of ciprofloxacin. General Hosp. Psychiatry 33, 82.e5–82.e7. 10.1016/j.genhosppsych.2010.07.002 21353135

[B5] Aho V. T. E. Pereira P. A. B. Voutilainen S. Paulin L. Pekkonen E. Auvinen P.. (2019). Gut microbiota in Parkinson?s disease: temporal stability and relations to disease progression. eBioMedicine 44, 691–707. 10.1016/j.ebiom.2019.05.064 31221587 PMC6606744

[B6] Aitbali Y. Ba-M'hamed S. Elhidar N. Nafis A. Soraa N. Bennis M. (2018). Glyphosate based- herbicide exposure affects gut microbiota, anxiety and depression-like behaviors in mice. Neurotoxicol. Teratol. 67, 44–49. 10.1016/j.ntt.2018.04.002 29635013

[B7] Aizawa E. Tsuji H. Asahara T. Takahashi T. Teraishi T. Yoshida S.. (2019). Bifidobacterium and Lactobacillus counts in the gut microbiota of patients with bipolar disorder and healthy controls. Front. Psychiatry 9:730. 10.3389/fpsyt.2018.00730 30713509 PMC6346636

[B8] Al Jowf G. I. Ahmed Z. T. Reijnders R. A. De Nijs L. Eijssen L. M. T. (2023). To predict, prevent, and manage post-traumatic stress disorder (PTSD): a review of pathophysiology, treatment, and biomarkers. IJMS 24:5238. 10.3390/ijms24065238 36982313 PMC10049301

[B9] Al-Chalabi A. Hardiman O. (2013). The epidemiology of ALS: a conspiracy of genes, environment and time. Nat. Rev. Neurol. 9, 617–628. 10.1038/nrneurol.2013.203 24126629

[B10] Aleksic D. Poleksic J. Agatonovic G. Djulejic V. Vulovic M. Aksic M.. (2023). The long-term effects of maternal deprivation on the number and size of inhibitory interneurons in the rat amygdala and nucleus accumbens. Front. Neurosci. 17:1187758. 10.3389/fnins.2023.1187758 37434764 PMC10330809

[B11] Aliev G. Beeraka N. M. Nikolenko V. N. Svistunov A. A. Rozhnova T. Kostyuk S.. (2020). Neurophysiology and psychopathology underlying PTSD and recent insights into the PTSD therapies—a comprehensive review. JCM 9:2951. 10.3390/jcm9092951 32932645 PMC7565106

[B12] Allegretti J. R. Kassam Z. Osman M. Budree S. Fischer M. Kelly C. R. (2018). The 5D framework: a clinical primer for fecal microbiota transplantation to treat Clostridium difficile infection. Gastrointest. Endosc. 87, 18–29. 10.1016/j.gie.2017.05.036 28583769

[B13] Alonso J. Petukhova M. Vilagut G. Chatterji S. Heeringa S. Üstün T. B.. (2011). Days out of role due to common physical and mental conditions: results from the WHO World Mental Health surveys. Mol. Psychiatry 16, 1234–1246. 10.1038/mp.2010.101 20938433 PMC3223313

[B14] Arcan C. Hou W. Hoffman K. Reichardt A. Yang X. Clouston S. A. P.. (2024). Mediterranean diet intervention among World Trade Center responders with post-traumatic stress disorder: feasibility and outcomes of a pilot randomized controlled trial. Obes. Sci. Pract. 10:e725. 10.1002/osp4.725 38263989 PMC10804354

[B15] Arnaud A. M. Brister T. S. Duckworth K. Foxworth P. Fulwider T. Suthoff E. D.. (2022). Impact of major depressive disorder on comorbidities: a systematic literature review. J. Clin. Psychiatry 83:43390. 10.4088/JCP.21r14328 36264099

[B16] Arnold F. J. Putka A. F. Raychaudhuri U. Hsu S. Bedlack R. S. Bennett C. L.. (2024). Revisiting glutamate excitotoxicity in amyotrophic lateral sclerosis and age-related neurodegeneration. Int. J. Mol. Sci. 25:5587. 10.3390/ijms25115587 38891774 PMC11171854

[B17] Arnold S. E. Arvanitakis Z. Macauley-Rambach S. L. Koenig A. M. Wang H.-Y. Ahima R. S.. (2018). Brain insulin resistance in type 2 diabetes and Alzheimer disease: concepts and conundrums. Nat. Rev. Neurol. 14, 168–181. 10.1038/nrneurol.2017.185 29377010 PMC6098968

[B18] Ashe K. H. (2020). The biogenesis and biology of amyloid β oligomers in the brain. Alzheimer. Dementia 16, 1561–1567. 10.1002/alz.12084 32543725 PMC7984270

[B19] Asher S. Priefer R. (2022). Alzheimer's disease failed clinical trials. Life Sci. 306:120861. 10.1016/j.lfs.2022.120861 35932841

[B20] Atarashi K. Tanoue T. Shima T. Imaoka A. Kuwahara T. Momose Y.. (2011). Induction of colonic regulatory T cells by indigenous *Clostridium* species. Science 331, 337–341. 10.1126/science.1198469 21205640 PMC3969237

[B21] Bahr S. M. Weidemann B. J. Castro A. N. Walsh J. W. deLeon O. Burnett C. M. L.. (2015). Risperidone-induced weight gain is mediated through shifts in the gut microbiome and suppression of energy expenditure. EBioMedicine 2, 1725–1734. 10.1016/j.ebiom.2015.10.018 26870798 PMC4740326

[B22] Bai J. Barandouzi Z. A. Rowcliffe C. Meador R. Tsementzi D. Bruner D. W. (2021). Gut microbiome and its associations with acute and chronic gastrointestinal toxicities in cancer patients with pelvic radiation therapy: a systematic review. Front. Oncol. 11:745262. 10.3389/fonc.2021.745262 34938654 PMC8685326

[B23] Bajaj J. S. Sikaroodi M. Fagan A. Heuman D. Gilles H. Gavis E. A.. (2019). Posttraumatic stress disorder is associated with altered gut microbiota that modulates cognitive performance in veterans with cirrhosis. Am. J. Physiol. Gastrointestinal Liver Physiol. 317, G661–G669. 10.1152/ajpgi.00194.2019 31460790 PMC6879889

[B24] Baldini F. Hertel J. Sandt E. Thinnes C. C. Neuberger-Castillo L. Pavelka L.. (2020). Parkinson's disease-associated alterations of the gut microbiome predict disease-relevant changes in metabolic functions. BMC Biol. 18:62. 10.1186/s12915-020-00775-7 32517799 PMC7285525

[B25] Bambling M. Edwards S. C. Hall S. Vitetta L. (2017). A combination of probiotics and magnesium orotate attenuate depression in a small SSRI resistant cohort: an intestinal anti-inflammatory response is suggested. Inflammopharmacology 25, 271–274. 10.1007/s10787-017-0311-x 28155119

[B26] Bansal A. S. Seton K. A. Brooks J. C. W. Carding S. R. (2025). Cognitive dysfunction in myalgic encephalomyelitis/chronic fatigue syndrome-aetiology and potential treatments. Int. J. Mol. Sci. 26:1896. 10.3390/ijms26051896 40076522 PMC11899462

[B27] Barandouzi Z. A. Starkweather A. R. Henderson W. A. Gyamfi A. Cong X. S. (2020). Altered composition of gut microbiota in depression: a systematic review. Front. Psychiatry 11:541. 10.3389/fpsyt.2020.00541 32587537 PMC7299157

[B28] Barati M. Ghahremani A. Namdar Ahmadabad H. (2023). Intermittent fasting: a promising dietary intervention for autoimmune diseases. Autoimmun. Rev. 22:103408. 10.1016/j.autrev.2023.103408 37572827

[B29] Barnett J. A. Josephson J. K. Haskey N. Hart M. M. Soma K. K. Verdugo A.. (2024). Prenatal exposure to dietary levels of glyphosate disrupts metabolic, immune, and behavioral markers across generations in mice. bioRxiv. 10.1101/2024.08.27.609990 41005169

[B30] Bäuerl C. Collado M. C. Diaz Cuevas A. Viña J. Pérez Martínez G. (2018). Shifts in gut microbiota composition in an APP/PSS1 transgenic mouse model of Alzheimer's disease during lifespan. Lett. Appl. Microbiol. 66, 464–471. 10.1111/lam.12882 29575030

[B31] Bayles K. A. McCullough K. C. Tomoeda C. K. (2018). Cognitive-Communication Disorders of MCI and Dementia: Definition, Assessment, and Clinical Management, 3rd Edn. San Diego, CA: Plural Publishing, Incorporated.

[B32] Bazzari F. H. Abdallah D. M. El-Abhar H. S. (2019). Pharmacological interventions to attenuate Alzheimer's disease progression: the story so far. CAR 16, 261–277. 10.2174/1567205016666190301111120 30827243

[B33] Bear T. Dalziel J. Coad J. Roy N. Butts C. Gopal P. (2021). The microbiome-gut-brain axis and resilience to developing anxiety or depression under stress. Microorganisms 9:723. 10.3390/microorganisms9040723 33807290 PMC8065970

[B34] Becker A. Schmartz G. P. Gröger L. Grammes N. Galata V. Philippeit H.. (2022). Effects of resistant starch on symptoms, fecal markers, and gut microbiota in Parkinson's disease — the RESISTA-PD trial. Genom. Proteomics Bioinformat. 20, 274–287. 10.1016/j.gpb.2021.08.009 34839011 PMC9684155

[B35] Bedarf J. R. Hildebrand F. Coelho L. P. Sunagawa S. Bahram M. Goeser F.. (2017). Erratum to: Functional implications of microbial and viral gut metagenome changes in early stage L-DOPA-naïve Parkinson's disease patients. Genome Med. 9:61. 10.1186/s13073-017-0451-z 28662719 PMC5492116

[B36] Benakis C. Brea D. Caballero S. Faraco G. Moore J. Murphy M.. (2016). Commensal microbiota affects ischemic stroke outcome by regulating intestinal γδ T cells. Nat. Med. 22, 516–523. 10.1038/nm.4068 27019327 PMC4860105

[B37] Ben-Zion Z. Korem N. Spiller T. R. Duek O. Keynan J. N. Admon R.. (2023). Longitudinal volumetric evaluation of hippocampus and amygdala subregions in recent trauma survivors. Mol. Psychiatry 28, 657–667. 10.1038/s41380-022-01842-x 36280750 PMC9918676

[B38] Bercik P. Denou E. Collins J. Jackson W. Lu J. Jury J.. (2011). The intestinal microbiota affect central levels of brain-derived neurotropic factor and behavior in mice. Gastroenterology 141, 599–609; 609.e1–3. 10.1053/j.gastro.2011.04.052 21683077

[B39] Bergold P. J. Pinkhasova V. Syed M. Kao H.-Y. Jozwicka A. Zhao N.. (2009). Production of panic-like symptoms by lactate is associated with increased neural firing and oxidation of brain redox in the rat hippocampus. Neurosci. Lett. 453, 219–224. 10.1016/j.neulet.2009.02.041 19429039

[B40] Bharwani A. Mian M. F. Surette M. G. Bienenstock J. Forsythe P. (2017). Oral treatment with *Lactobacillus rhamnosus* attenuates behavioural deficits and immune changes in chronic social stress. BMC Med. 15:7. 10.1186/s12916-016-0771-7 28073366 PMC5225647

[B41] Bibi A. Zhang F. Shen J. Din A. U. Xu Y. (2025). Behavioral alterations in antibiotic-treated mice associated with gut microbiota dysbiosis: insights from 16S rRNA and metabolomics. Front. Neurosci. 19:1478304. 10.3389/fnins.2025.1478304 40092066 PMC11906700

[B42] Biessels G. J. Reagan L. P. (2015). Hippocampal insulin resistance and cognitive dysfunction. Nat. Rev. Neurosci. 16, 660–671. 10.1038/nrn4019 26462756

[B43] Bobo W. V. Grossardt B. R. Virani S. St Sauver J. L. Boyd C. M. Rocca W. A. (2022). Association of depression and anxiety with the accumulation of chronic conditions. JAMA Netw. Open 5:e229817. 10.1001/jamanetworkopen.2022.9817 35499825 PMC9062691

[B44] Boillée S. Yamanaka K. Lobsiger C. S. Copeland N. G. Jenkins N. A. Kassiotis G.. (2006). Onset and progression in inherited ALS determined by motor neurons and microglia. Science 312, 1389–1392. 10.1126/science.1123511 16741123

[B45] Bonfili L. Cecarini V. Gogoi O. Berardi S. Scarpona S. Angeletti M.. (2020). Gut microbiota manipulation through probiotics oral administration restores glucose homeostasis in a mouse model of Alzheimer's disease. Neurobiol. Aging 87, 35–43. 10.1016/j.neurobiolaging.2019.11.004 31813629

[B46] Borkent J. Ioannou M. Laman J. D. Haarman B. C. M. Sommer I. E. C. (2022). Role of the gut microbiome in three major psychiatric disorders. Psychol. Med. 52, 1222–1242. 10.1017/S0033291722000897 35506416 PMC9157303

[B47] Borody T. J. Brandt L. J. Paramsothy S. (2014). Therapeutic faecal microbiota transplantation: current status and future developments. Curr. Opin. Gastroenterol. 30, 97–105. 10.1097/MOG.0000000000000027 24257037 PMC3868025

[B48] Borzabadi S. Oryan S. Eidi A. Aghadavod E. Daneshvar Kakhaki R. Tamtaji O. R.. (2018). The effects of probiotic supplementation on gene expression related to inflammation, insulin and lipid in patients with Parkinson's disease: a randomized, double-blind, placebocontrolled trial. Arch. Iran. Med. 21, 289–295. 30041526

[B49] Bourgonje A. R. Hörstke N. V. Fehringer M. Innocenti G. Vogl T. (2024). Systemic antibody responses against gut microbiota flagellins implicate shared and divergent immune reactivity in Crohn's disease and chronic fatigue syndrome. Microbiome 12:141. 10.1186/s40168-024-01858-1 39075559 PMC11285207

[B50] Bravo J. A. Forsythe P. Chew M. V. Escaravage E. Savignac H. M. Dinan T. G.. (2011). Ingestion of Lactobacillus strain regulates emotional behavior and central GABA receptor expression in a mouse via the vagus nerve. Proc. Natl. Acad. Sci. U.S.A. 108, 16050–16055. 10.1073/pnas.1102999108 21876150 PMC3179073

[B51] Brenner D. Yilmaz R. Müller K. Grehl T. Petri S. Meyer T.. (2018). Hot-spot KIF5A mutations cause familial ALS. Brain 141, 688–697. 10.1093/brain/awx370 29342275 PMC5837483

[B52] Brenner L. A. Forster J. E. Stearns-Yoder K. A. Stamper C. E. Hoisington A. J. Brostow D. P.. (2020). Evaluation of an immunomodulatory probiotic intervention for veterans with co-occurring mild traumatic brain injury and posttraumatic stress disorder: a pilot study. Front. Neurol. 11:1015. 10.3389/fneur.2020.01015 33192959 PMC7641622

[B53] Brotman R. G. Moreno-Escobar M. C. Joseph J. Munakomi S. Pawar G. (2025). “Amyotrophic Lateral Sclerosis,” in StatPearls (Treasure Island, FL: StatPearls Publishing). Available online at: http://www.ncbi.nlm.nih.gov/books/NBK556151/ (Accessed July 9, 2025). 32310611

[B54] Bruun C. F. Haldor Hansen T. Vinberg M. Kessing L. V. Coello K. (2024). Associations between short-chain fatty acid levels and mood disorder symptoms: a systematic review. Nutr. Neurosci. 27, 899–912. 10.1080/1028415X.2023.2277970 37976103

[B55] Buchenauer L. Haange S.-B. Bauer M. Rolle-Kampczyk U. E. Wagner M. Stucke J.. (2023). Maternal exposure of mice to glyphosate induces depression- and anxiety-like behavior in the offspring via alterations of the gut-brain axis. Sci. Total Environ. 905:167034. 10.1016/j.scitotenv.2023.167034 37709081

[B56] Burokas A. Arboleya S. Moloney R. D. Peterson V. L. Murphy K. Clarke G.. (2017). Targeting the microbiota-gut-brain axis: prebiotics have anxiolytic and antidepressant-like effects and reverse the impact of chronic stress in mice. Biol. Psychiatry 82, 472–487. 10.1016/j.biopsych.2016.12.031 28242013

[B57] Burton T. C. Lv N. Tsai P. Peñalver Bernabé B. Tussing-Humphreys L. Xiao L.. (2023). Associations between fecal short-chain fatty acids, plasma inflammatory cytokines, and dietary markers with depression and anxiety: post hoc analysis of the ENGAGE-2 pilot trial. Am. J. Clin. Nutr. 117, 717–730. 10.1016/j.ajcnut.2023.01.018 36796440 PMC10273083

[B58] Butler M. I. Bastiaanssen T. F. S. Long-Smith C. Morkl S. Berding K. Ritz N. L.. (2023). The gut microbiome in social anxiety disorder: evidence of altered composition and function. Transl. Psychiatry 13:95. 10.1038/s41398-023-02325-5 36941248 PMC10027687

[B59] Butler M. I. Long-Smith C. Moloney G. M. Morkl S. O'Mahony S. M. Cryan J. F.. (2022). The immune-kynurenine pathway in social anxiety disorder. Brain Behav. Immun. 99, 317–326. 10.1016/j.bbi.2021.10.020 34758380

[B60] Cai M. Xue S.-S. Zhou C.-H. Feng Y.-C. Liu J.-Z. Liu R.. (2025). Effects of fecal microbiota transplantation from patients with generalized anxiety on anxiety-like behaviors: the role of the gut-microbiota-endocannabinoid-brain axis. J. Affect. Disord. 381, 131–149. 10.1016/j.jad.2025.04.018 40187430

[B61] Campbell D. Green M. J. Davies N. Demou E. Howe L. D. Harrison S.. (2022). Effects of depression on employment and social outcomes: a Mendelian randomisation study. J. Epidemiol. Community Health 76, 563–571. 10.1136/jech-2021-218074 35318279 PMC9118074

[B62] Canet G. Hernandez C. Zussy C. Chevallier N. Desrumaux C. Givalois L. (2019). Is AD a stress-related disorder? Focus on the HPA axis and its promising therapeutic targets. Front. Aging Neurosci. 11:269. 10.3389/fnagi.2019.00269 31611783 PMC6776918

[B63] Cantarel B. L. Waubant E. Chehoud C. Kuczynski J. DeSantis T. Z. Warrington J.. (2015). Gut microbiota in multiple sclerosis: possible influence of immunomodulators. J. Investig. Med. 63, 729–734. 10.1097/JIM.0000000000000192 25775034 PMC4439263

[B64] Cao J. Amakye W. K. Qi C. Liu X. Ma J. Ren J. (2021). *Bifidobacterium lactis* Probio-M8 regulates gut microbiota to alleviate Alzheimer's disease in the APP/PS1 mouse model. Eur. J. Nutr. 60, 3757–3769. 10.1007/s00394-021-02543-x 33796919

[B65] Carrasco-Querol N. Cabricano-Canga L. Bueno Hernández N. Gonçalves A. Q. Caballol Angelats R. Pozo Ariza M.. (2024). Nutrition and chronobiology as key components of multidisciplinary therapeutic interventions for fibromyalgia and associated chronic fatigue syndrome: a narrative and critical review. Nutrients 16:182. 10.3390/nu16020182 38257075 PMC10818822

[B66] Cassani E. Privitera G. Pezzoli G. Pusani C. Madio C. Iorio L.. (2011). Use of probiotics for the treatment of constipation in Parkinson's disease patients. Minerva Gastroenterol. Dietol. 57, 117–121. 21587143

[B67] Catron T. R. Gaballah S. Tal T. (2019). Using zebrafish to investigate interactions between xenobiotics and microbiota. Curr. Pharmacol. Rep. 5, 468–480. 10.1007/s40495-019-00203-735415967

[B68] Cattaneo A. Cattane N. Galluzzi S. Provasi S. Lopizzo N. Festari C.. (2017). Association of brain amyloidosis with pro-inflammatory gut bacterial taxa and peripheral inflammation markers in cognitively impaired elderly. Neurobiol. Aging 49, 60–68. 10.1016/j.neurobiolaging.2016.08.019 27776263

[B69] Cekanaviciute E. Yoo B. B. Runia T. F. Debelius J. W. Singh S. Nelson C. A.. (2017). Gut bacteria from multiple sclerosis patients modulate human T cells and exacerbate symptoms in mouse models. Proc. Natl. Acad. Sci. U.S.A. 114, 10713–10718. 10.1073/pnas.1711235114 28893978 PMC5635915

[B70] Cersosimo M. G. Raina G. B. Pecci C. Pellene A. Calandra C. R. Gutiérrez C.. (2013). Gastrointestinal manifestations in Parkinson's disease: prevalence and occurrence before motor symptoms. J. Neurol. 260, 1332–1338. 10.1007/s00415-012-6801-2 23263478

[B71] Chakraborty N. Holmes-Hampton G. Rusling M. Kumar V. P. Hoke A. Lawrence A. B.. (2025). Delayed impact of ionizing radiation depends on sex: integrative metagenomics and metabolomics analysis of rodent colon content. Int. J. Mol. Sci. 26:4227. 10.3390/ijms26094227 40362462 PMC12071923

[B72] Chandra S. Sisodia S. S. Vassar R. J. (2023). The gut microbiome in Alzheimer's disease: what we know and what remains to be explored. Mol. Neurodegener. 18:9. 10.1186/s13024-023-00595-7 36721148 PMC9889249

[B73] Charney D. S. (2003). The psychobiology of resilience and vulnerability to anxiety disorders: implications for prevention and treatment. Dial. Clin. Neurosci. 5, 207–221. 10.31887/DCNS.2003.5.3/dcharney 22034473 PMC3181630

[B74] Chen D. Yang X. Yang J. Lai G. Yong T. Tang X.. (2017). Prebiotic effect of fructooligosaccharides from *Morinda officinalis* on Alzheimer's disease in rodent models by targeting the microbiota-gut-brain axis. Front. Aging Neurosci. 9:403. 10.3389/fnagi.2017.00403 29276488 PMC5727096

[B75] Chen J. Chia N. Kalari K. R. Yao J. Z. Novotna M. Paz Soldan M. M.. (2016). Multiple sclerosis patients have a distinct gut microbiota compared to healthy controls. Sci. Rep. 6:28484. 10.1038/srep28484 27346372 PMC4921909

[B76] Chen X. Xu L. Chen Q. Su S. Zhuang J. Qiao D. (2023). Polystyrene micro- and nanoparticles exposure induced anxiety-like behaviors, gut microbiota dysbiosis and metabolism disorder in adult mice. Ecotoxicol. Environ. Saf. 259:115000. 10.1016/j.ecoenv.2023.115000 37210994

[B77] Chen Y. Bai J. Wu D. Yu S. Qiang X. Bai H.. (2019). Association between fecal microbiota and generalized anxiety disorder: severity and early treatment response. J. Affect. Disord. 259, 56–66. 10.1016/j.jad.2019.08.014 31437702

[B78] Chen Y. Xue F. Yu S. Li X. Liu L. Jia Y.. (2021). Gut microbiota dysbiosis in depressed women: the association of symptom severity and microbiota function. J. Affect. Disord. 282, 391–400. 10.1016/j.jad.2020.12.143 33421868

[B79] Chiang J. Y. L. Ferrell J. M. (2019). Bile acids as metabolic regulators and nutrient sensors. Annu. Rev. Nutr. 39, 175–200. 10.1146/annurev-nutr-082018-124344 31018107 PMC6996089

[B80] Chiantia G. Comai D. Hidisoglu E. Gurgone A. Franchino C. Carabelli V.. (2025). Glyphosate impairs both structure and function of GABAergic synapses in hippocampal neurons. Neuropharmacology 262:110183. 10.1016/j.neuropharm.2024.110183 39401670

[B81] Chiò A. Logroscino G. Traynor B. J. Collins J. Simeone J. C. Goldstein L. A.. (2013). Global epidemiology of amyotrophic lateral sclerosis: a systematic review of the published literature. Neuroepidemiology 41, 118–130. 10.1159/000351153 23860588 PMC4049265

[B82] Chung Y.-C. E. Chen H.-C. Chou H.-C. L. Chen I.-M. Lee M.-S. Chuang L.-C.. (2019). Exploration of microbiota targets for major depressive disorder and mood related traits. J. Psychiatr. Res. 111, 74–82. 10.1016/j.jpsychires.2019.01.016 30685565

[B83] Church J. S. Bannish J. A. M. Adrian L. A. Rojas Martinez K. Henshaw A. Schwartzer J. J. (2023). Serum short chain fatty acids mediate hippocampal BDNF and correlate with decreasing neuroinflammation following high pectin fiber diet in mice. Front. Neurosci. 17:1134080. 10.3389/fnins.2023.1134080 37123365 PMC10130583

[B84] Clark I. Atwood C. Bowen R. Paz-Filho G. Vissel B. (2012). Tumor necrosis factor-induced cerebral insulin resistance in Alzheimer's disease links numerous treatment rationales. Pharmacol. Rev. 64, 1004–1026. 10.1124/pr.112.005850 22966039

[B85] Clarke G. Grenham S. Scully P. Fitzgerald P. Moloney R. D. Shanahan F.. (2013). The microbiome-gut-brain axis during early life regulates the hippocampal serotonergic system in a sex-dependent manner. Mol. Psychiatry 18, 666–673. 10.1038/mp.2012.77 22688187

[B86] Colpitts S. L. Kasper E. J. Keever A. Liljenberg C. Kirby T. Magori K.. (2017). A bidirectional association between the gut microbiota and CNS disease in a biphasic murine model of multiple sclerosis. Gut Microbes 8, 561–573. 10.1080/19490976.2017.1353843 28708466 PMC5730387

[B87] Conesa M. P. B. Blixt F. W. Peesh P. Khan R. Korf J. Lee J.. (2023). Stabilizing histamine release in gut mast cells mitigates peripheral and central inflammation after stroke. J. Neuroinflamm. 20:230. 10.1186/s12974-023-02887-7 37805585 PMC10560441

[B88] Cools R. D'Esposito M. (2011). Inverted-U–shaped dopamine actions on human working memory and cognitive control. Biol. Psychiatry 69, e113–e125. 10.1016/j.biopsych.2011.03.028 21531388 PMC3111448

[B89] Cosorich I. Dalla-Costa G. Sorini C. Ferrarese R. Messina M. J. Dolpady J.. (2017). High frequency of intestinal T_H_ 17 cells correlates with microbiota alterations and disease activity in multiple sclerosis. Sci. Adv. 3:e1700492. 10.1126/sciadv.1700492 28706993 PMC5507635

[B90] Costabile A. Buttarazzi I. Kolida S. Quercia S. Baldini J. Swann J. R.. (2017). An *in vivo* assessment of the cholesterol-lowering efficacy of *Lactobacillus plantarum* ECGC 13110402 in normal to mildly hypercholesterolaemic adults. PLoS ONE 12:e0187964. 10.1371/journal.pone.0187964 29228000 PMC5724841

[B91] Costas-Ferreira C. Durán R. Faro L. R. F. (2022). Toxic effects of glyphosate on the nervous system: a systematic review. IJMS 23:4605. 10.3390/ijms23094605 35562999 PMC9101768

[B92] Crile G. (1951). Vagotomy in the treatment of biliary dyskinesia. Arch. Surg. 63:687. 10.1001/archsurg.1951.01250040701017 14868231

[B93] Cryan J. F. O'Mahony S. M. (2011). The microbiome-gut-brain axis: from bowel to behavior. Neurogastroenterol. Motil. 23, 187–192. 10.1111/j.1365-2982.2010.01664.x 21303428

[B94] Cui L. Li S. Wang S. Wu X. Liu Y. Yu W.. (2024). Major depressive disorder: hypothesis, mechanism, prevention and treatment. Sig. Transduct. Target. Ther. 9:30. 10.1038/s41392-024-01738-y 38331979 PMC10853571

[B95] Cui M. Xiao H. Li Y. Dong J. Luo D. Li H.. (2017a). Total abdominal irradiation exposure impairs cognitive function involving miR-34a-5p/BDNF axis. Biochim. Biophys. Acta 1863, 2333–2341. 10.1016/j.bbadis.2017.06.021 28668331 PMC5578400

[B96] Cui M. Xiao H. Li Y. Zhou L. Zhao S. Luo D.. (2017b). Faecal microbiota transplantation protects against radiation-induced toxicity. EMBO Mol. Med. 9, 448–461. 10.15252/emmm.201606932 28242755 PMC5376756

[B97] Dănău A. Dumitrescu L. Lefter A. Tulbă D. Popescu B. O. (2021). Small intestinal bacterial overgrowth as potential therapeutic target in Parkinson's disease. IJMS 22:11663. 10.3390/ijms222111663 34769091 PMC8584211

[B98] Dai L. Liu Z. Zhou W. Zhang L. Miao M. Wang L.. (2024). Sijunzi decoction, a classical Chinese herbal formula, improves fatigue symptoms with changes in gut microbiota in chronic fatigue syndrome: a randomized, double-blind, placebo-controlled, multi-center clinical trial. Phytomedicine 129:155636. 10.1016/j.phymed.2024.155636 38640860

[B99] Davis A. K. Barrett F. S. May D. G. Cosimano M. P. Sepeda N. D. Johnson M. W.. (2021). Effects of psilocybin-assisted therapy on major depressive disorder: a randomized clinical trial. JAMA Psychiatry 78:481. 10.1001/jamapsychiatry.2020.3285 33146667 PMC7643046

[B100] De Felice F. G. Gonçalves R. A. Ferreira S. T. (2022). Impaired insulin signalling and allostatic load in Alzheimer disease. Nat. Rev. Neurosci. 23, 215–230. 10.1038/s41583-022-00558-9 35228741

[B101] De Filippis F. Pellegrini N. Vannini L. Jeffery I. B. La Storia A. Laghi L.. (2016). High-level adherence to a Mediterranean diet beneficially impacts the gut microbiota and associated metabolome. Gut 65, 1812–1821. 10.1136/gutjnl-2015-309957 26416813

[B102] De Filippis F. Valentino V. Sequino G. Borriello G. Riccardi M. G. Pierri B.. (2024). Exposure to environmental pollutants selects for xenobiotic-degrading functions in the human gut microbiome. Nat. Commun. 15:4482. 10.1038/s41467-024-48739-7 38802370 PMC11130323

[B103] De Palma G. Collins S. M. Bercik P. (2014). The microbiota-gut-brain axis in functional gastrointestinal disorders. Gut Microbes 5, 419–429. 10.4161/gmic.29417 24921926 PMC4153782

[B104] De Punder K. Pruimboom L. (2015). Stress induces endotoxemia and low-grade inflammation by increasing barrier permeability. Front. Immunol. 6:223. 10.3389/fimmu.2015.00223 26029209 PMC4432792

[B105] De Vries Y. A. De Jonge P. Van Den Heuvel E. Turner E. H. Roest A. M. (2016). Influence of baseline severity on antidepressant efficacy for anxiety disorders: Meta-analysis and meta-regression. Br. J. Psychiatry 208, 515–521. 10.1192/bjp.bp.115.173450 26989093

[B106] Del Castilo I. Neumann A. S. Lemos F. S. De Bastiani M. A. Oliveira F. L. Zimmer E. R.. (2022). Lifelong exposure to a low-dose of the glyphosate-based herbicide RoundUp^®^ causes intestinal damage, gut dysbiosis, and behavioral changes in mice. IJMS 23:5583. 10.3390/ijms23105583 35628394 PMC9146949

[B107] Deng W. Yi P. Xiong Y. Ying J. Lin Y. Dong Y.. (2024). Gut metabolites acting on the gut-brain axis: regulating the functional state of microglia. Aging Dis. 15, 480–502. 10.14336/AD.2023.0727 37548933 PMC10917527

[B108] Desbonnet L. Clarke G. Traplin A. O'Sullivan O. Crispie F. Moloney R. D.. (2015). Gut microbiota depletion from early adolescence in mice: implications for brain and behaviour. Brain Behav. Immun. 48, 165–173. 10.1016/j.bbi.2015.04.004 25866195

[B109] Dickerson F. Adamos M. Katsafanas E. Khushalani S. Origoni A. Savage C.. (2018). Adjunctive probiotic microorganisms to prevent rehospitalization in patients with acute mania: a randomized controlled trial. Bipolar Disord. 20, 614–621. 10.1111/bdi.12652 29693757

[B110] Ding X. Li Q. Li P. Chen X. Xiang L. Bi L.. (2020). Fecal microbiota transplantation: a promising treatment for radiation enteritis? Radiother. Oncol. 143, 12–18. 10.1016/j.radonc.2020.01.011 32044171

[B111] Ding Y. Bu F. Chen T. Shi G. Yuan X. Feng Z.. (2021). A next-generation probiotic: Akkermansia muciniphila ameliorates chronic stress–induced depressive-like behavior in mice by regulating gut microbiota and metabolites. Appl. Microbiol. Biotechnol. 105, 8411–8426. 10.1007/s00253-021-11622-2 34617139

[B112] Dobbs S. M. Dobbs R. J. Weller C. Charlett A. Bjarnason I. T. Lawson A. J.. (2010). Differential effect of *Helicobacter pylori* eradication on time-trends in brady/hypokinesia and rigidity in idiopathic parkinsonism: report on completion of a randomized, double-blind, placebo-controlled efficacy study. Helicobacter 15, 279–294. 10.1111/j.1523-5378.2010.00768.x 20633189 PMC2913104

[B113] Dobson R. Giovannoni G. (2019). Multiple sclerosis – a review. Euro. J. Neurol. 26, 27–40. 10.1111/ene.13819 30300457

[B114] Dodiya H. B. Kuntz T. Shaik S. M. Baufeld C. Leibowitz J. Zhang X.. (2019). Sex-specific effects of microbiome perturbations on cerebral Aβ amyloidosis and microglia phenotypes. J. Exp. Med. 216, 1542–1560. 10.1084/jem.20182386 31097468 PMC6605759

[B115] Doll J. P. K. Vázquez-Castellanos J. F. Schaub A.-C. Schweinfurth N. Kettelhack C. Schneider E.. (2022). Fecal microbiota transplantation (FMT) as an adjunctive therapy for depression—case report. Front. Psychiatry 13:815422. 10.3389/fpsyt.2022.815422 35250668 PMC8891755

[B116] Dong Z. Shen X. Hao Y. Li J. Xu H. Yin L.. (2022). Gut microbiome: a potential indicator for predicting treatment outcomes in major depressive disorder. Front. Neurosci. 16:813075. 10.3389/fnins.2022.813075 35937875 PMC9354493

[B117] Dressman D. Elyaman W. (2022). T cells: a growing universe of roles in neurodegenerative diseases. Neuroscientist 28, 335–348. 10.1177/10738584211024907 34160330

[B118] Drzewiecki C. M. Fox A. S. (2024). Understanding the heterogeneity of anxiety using a translational neuroscience approach. Cogn. Affect. Behav. Neurosci. 24, 228–245. 10.3758/s13415-024-01162-3 38356013 PMC11039504

[B119] Du Y. He C. An Y. Huang Y. Zhang H. Fu W.. (2024). The role of short chain fatty acids in inflammation and body health. Int. J. Mol. Sci. 25:7379. 10.3390/ijms25137379 39000498 PMC11242198

[B120] Duan Y. Wu X. Liang S. Jin F. (2015). Elevated blood ammonia level is a potential biological risk factor of behavioral disorders in prisoners. Behav. Neurol. 2015, 1–5. 10.1155/2015/797862 26457003 PMC4589609

[B121] Dupuis L. Chio A. (2023). The “metabolic axis” of ALS: the role of body weight in disease pathogenesis. Muscle Nerve 67, 191–192. 10.1002/mus.27784 36602891

[B122] Eijsbouts C. Zheng T. Kennedy N. A. Bonfiglio F. Anderson C. A. Moutsianas L.. (2021). Genome-wide analysis of 53,400 people with irritable bowel syndrome highlights shared genetic pathways with mood and anxiety disorders. Nat. Genet. 53, 1543–1552. 10.1038/s41588-021-00950-8 34741163 PMC8571093

[B123] Elendu C. Amaechi D. C. Elendu T. C. Ibhiedu J. O. Egbunu E. O. Ndam A. R.. (2023). Stroke and cognitive impairment: understanding the connection and managing symptoms. Ann. Med. Surg. 85, 6057–6066. 10.1097/MS9.0000000000001441 38098605 PMC10718363

[B124] Elfil M. Kamel S. Kandil M. Koo B. B. Schaefer S. M. (2020). Implications of the gut microbiome in Parkinson's disease. Mov. Disord. 35, 921–933. 10.1002/mds.28004 32092186

[B125] El-Sehrawy A. A. M. A. Ayoub I. I. Uthirapathy S. Ballal S. Gabble B. C. Singh A.. (2025). The microbiota-gut-brain axis in myalgic encephalomyelitis/chronic fatigue syndrome: a narrative review of an emerging field. Eur. J. Transl. Myol. 35:13690. 10.4081/ejtm.2025.13690 39937103 PMC12038572

[B126] Emery D. C. Shoemark D. K. Batstone T. E. Waterfall C. M. Coghill J. A. Cerajewska T. L.. (2017). 16S rRNA next generation sequencing analysis shows bacteria in Alzheimer's post-mortem brain. Front. Aging Neurosci. 9:195. 10.3389/fnagi.2017.00195 28676754 PMC5476743

[B127] Engen P. A. Zaferiou A. Rasmussen H. Naqib A. Green S. J. Fogg L. F.. (2020). Single-arm, non-randomized, time series, single-subject study of fecal microbiota transplantation in multiple sclerosis. Front. Neurol. 11:978. 10.3389/fneur.2020.00978 33013647 PMC7506051

[B128] Eraly S. A. Nievergelt C. M. Maihofer A. X. Barkauskas D. A. Biswas N. Agorastos A.. (2014). Assessment of plasma C-reactive protein as a biomarker of posttraumatic stress disorder risk. JAMA Psychiatry 71:423. 10.1001/jamapsychiatry.2013.4374 24576974 PMC4032578

[B129] Erny D. Hrabě De Angelis A. L. Jaitin D. Wieghofer P. Staszewski O. David E.. (2015). Host microbiota constantly control maturation and function of microglia in the CNS. Nat. Neurosci. 18, 965–977. 10.1038/nn.4030 26030851 PMC5528863

[B130] Escartin C. Galea E. Lakatos A. O'Callaghan J. P. Petzold G. C. Serrano-Pozo A.. (2021). Reactive astrocyte nomenclature, definitions, and future directions. Nat. Neurosci. 24, 312–325. 10.1038/s41593-020-00783-4 33589835 PMC8007081

[B131] Eslami Shahrbabaki M. Sabouri S. Sabahi A. Barfeh D. Divsalar P. Esmailzadeh M.. (2020). The efficacy of probiotics for treatment of bipolar disorder-type 1: a randomized, double-blind, placebo controlled trial. Iran. J. Psychiatry 15, 10–16. 32377210 PMC7193240

[B132] Evans S. J. Bassis C. M. Hein R. Assari S. Flowers S. A. Kelly M. B.. (2017). The gut microbiome composition associates with bipolar disorder and illness severity. J. Psychiatr. Res. 87, 23–29. 10.1016/j.jpsychires.2016.12.007 27988330 PMC5336480

[B133] Faravelli C. (2012). Childhood stressful events, HPA axis and anxiety disorders. WJP 2:13. 10.5498/wjp.v2.i1.13 24175164 PMC3782172

[B134] Fasano A. Bove F. Gabrielli M. Petracca M. Zocco M. A. Ragazzoni E.. (2013). The role of small intestinal bacterial overgrowth in Parkinson's disease. Mov. Disord. 28, 1241–1249. 10.1002/mds.25522 23712625

[B135] Felice V. D. Quigley E. M. Sullivan A. M. O'Keeffe G. W. O'Mahony S. M. (2016). Microbiota-gut-brain signalling in Parkinson's disease: implications for non-motor symptoms. Parkinson. Relat. Disord. 27, 1–8. 10.1016/j.parkreldis.2016.03.012 27013171

[B136] Flowers S. A. Evans S. J. Ward K. M. McInnis M. G. Ellingrod V. L. (2017). Interaction between atypical antipsychotics and the gut microbiome in a bipolar disease cohort. Pharmacotherapy 37, 261–267. 10.1002/phar.1890 28035686

[B137] Fournier E. Leveque M. Ruiz P. Ratel J. Durif C. Chalancon S.. (2023). Microplastics: What happens in the human digestive tract? First evidences in adults using *in vitro* gut models. J. Hazard. Mater. 442:130010. 10.1016/j.jhazmat.2022.130010 36182891

[B138] Frost G. R. Li Y.-M. (2017). The role of astrocytes in amyloid production and Alzheimer's disease. Open Biol. 7:170228. 10.1098/rsob.170228 29237809 PMC5746550

[B139] Fung T. C. Olson C. A. Hsiao E. Y. (2017). Interactions between the microbiota, immune and nervous systems in health and disease. Nat. Neurosci. 20, 145–155. 10.1038/nn.4476 28092661 PMC6960010

[B140] Funk K. E. Mrak R. E. Kuret J. (2011). Granulovacuolar degeneration (GVD) bodies of Alzheimer's disease (AD) resemble late-stage autophagic organelles: granulovacuolar degeneration bodies and autophagy. Neuropathol. Appl. Neurobiol. 37, 295–306. 10.1111/j.1365-2990.2010.01135.x 20946470 PMC3037976

[B141] Gabrielli M. Bonazzi P. Scarpellini E. Bendia E. Lauritano E. C. Fasano A.. (2011). Prevalence of small intestinal bacterial overgrowth in Parkinson's disease. Mov. Disord. 26, 889–892. 10.1002/mds.23566 21520278

[B142] Galland L. (2014). The Gut Microbiome and the Brain. J. Med. Food 17, 1261–1272. 10.1089/jmf.2014.7000 25402818 PMC4259177

[B143] Gandhi K. R. Saadabadi A. (2025). “Levodopa (L-Dopa),” in StatPearls, (Treasure Island, FL: StatPearls Publishing). Available online at: http://www.ncbi.nlm.nih.gov/books/NBK482140/ (Accessed June 9, 2025).

[B144] Gareau M. G. Wine E. Rodrigues D. M. Cho J. H. Whary M. T. Philpott D. J.. (2011). Bacterial infection causes stress-induced memory dysfunction in mice. Gut 60, 307–317. 10.1136/gut.2009.202515 20966022

[B145] Georgescu D. Ancusa O. Georgescu L. Ionita I. Reisz D. (2016). Nonmotor gastrointestinal disorders in older patients with Parkinson's disease: is there hope? CIA 11, 1601–1608. 10.2147/CIA.S106284 27956826 PMC5113937

[B146] Girgenti M. J. Traumatic Stress Brain Research Group, Wang, J. Ji D. Cruz D. A. Stein M. B.. (2021). Transcriptomic organization of the human brain in post-traumatic stress disorder. Nat. Neurosci. 24, 24–33. 10.1038/s41593-020-00748-7 33349712

[B147] Glenner G. G. Wong C. W. (1984). Alzheimer's disease: Initial report of the purification and characterization of a novel cerebrovascular amyloid protein. Biochem. Biophys. Res. Commun. 120, 885–890. 10.1016/S0006-291X(84)80190-4 6375662

[B148] Godoy L. D. Rossignoli M. T. Delfino-Pereira P. Garcia-Cairasco N. De Lima Umeoka E. H. (2018). A comprehensive overview on stress neurobiology: basic concepts and clinical implications. Front. Behav. Neurosci. 12:127. 10.3389/fnbeh.2018.00127 30034327 PMC6043787

[B149] Gökçe Çokal B. Yurtdaş M. Keskin Güler S. Güneş H. N. Ataç Uçar C. Aytaç B.. (2017). Serum glutathione peroxidase, xanthine oxidase, and superoxide dismutase activities and malondialdehyde levels in patients with Parkinson's disease. Neurol. Sci. 38, 425–431. 10.1007/s10072-016-2782-8 27900485

[B150] Góralczyk-Bińkowska A. Szmajda-Krygier D. Kozłowska E. (2022). The microbiota-gut-brain axis in psychiatric disorders. Int. J. Mol. Sci. 23:11245. 10.3390/ijms231911245 36232548 PMC9570195

[B151] Goutman S. A. Hardiman O. Al-Chalabi A. Chió A. Savelieff M. G. Kiernan M. C.. (2022). Emerging insights into the complex genetics and pathophysiology of amyotrophic lateral sclerosis. Lancet Neurol. 21, 465–479. 10.1016/S1474-4422(21)00414-2 35334234 PMC9513754

[B152] Grande I. Berk M. Birmaher B. Vieta E. (2016). Bipolar disorder. Lancet 387, 1561–1572. 10.1016/S0140-6736(15)00241-X 26388529

[B153] Grant S. M. DeMorrow S. (2020). Bile acid signaling in neurodegenerative and neurological disorders. IJMS 21:5982. 10.3390/ijms21175982 32825239 PMC7503576

[B154] Green J. E. Berk M. Mohebbi M. Loughman A. McGuinness A. J. Castle D.. (2023). Feasibility, acceptability, and safety of faecal microbiota transplantation in the treatment of major depressive disorder: a pilot randomized controlled trial. Can. J. Psychiatry 68, 315–326. 10.1177/07067437221150508 36637229 PMC10192831

[B155] Grover M. Kolla B. P. Pamarthy R. Mansukhani M. P. Breen-Lyles M. He J.-P.. (2021). Psychological, physical, and sleep comorbidities and functional impairment in irritable bowel syndrome: results from a national survey of U.S. adults. PLoS ONE 16:e0245323. 10.1371/journal.pone.0245323 33444383 PMC7808669

[B156] Gudnadottir U. Kamau N. Fornes R. Nguyen M. H. Callens S. Fransson E.. (2024). Antibiotic or gastric acid inhibitor use during pregnancy and postpartum depression: Population-based cohort study. Acta Obstet. Gynecol. Scand. 103, 1596–1605. 10.1111/aogs.14864 38831623 PMC11266723

[B157] Guillemin L. Hofstede J. Anderson T. Walkeden H. Schellenberg K. Kang E.. (2022). Perceptions and experiences of nutrition interventions in individuals with amyotrophic lateral sclerosis (ALS) and their caregivers. Can. J. Diet. Pract. Res. 83, 193–197. 10.3148/cjdpr-2022-014 36004737

[B158] Guo C. Che X. Briese T. Ranjan A. Allicock O. Yates R. A.. (2023). Deficient butyrate-producing capacity in the gut microbiome is associated with bacterial network disturbances and fatigue symptoms in ME/CFS. Cell Host Microbe 31, 288–304.e8. 10.1016/j.chom.2023.01.004 36758522 PMC10183837

[B159] Guyton K. Z. Loomis D. Grosse Y. El Ghissassi F. Benbrahim-Tallaa L. Guha N.. (2015). Carcinogenicity of tetrachlorvinphos, parathion, malathion, diazinon, and glyphosate. Lancet Oncol. 16, 490–491. 10.1016/S1470-2045(15)70134-8 25801782

[B160] Guzzetta K. E. Cryan J. F. O'Leary O. F. (2022). Microbiota-gut-brain axis regulation of adult hippocampal neurogenesis. Brain Plast 8, 97–119. 10.3233/BPL-220141 36448039 PMC9661352

[B161] Haba R. Shintani N. Onaka Y. Wang H. Takenaga R. Hayata A.. (2012). Lipopolysaccharide affects exploratory behaviors toward novel objects by impairing cognition and/or motivation in mice: possible role of activation of the central amygdala. Behav. Brain Res. 228, 423–431. 10.1016/j.bbr.2011.12.027 22209851

[B162] Hamad M. I. K. Daoud S. Petrova P. Rabaya O. Jbara A. Al Houqani S.. (2024a). Reelin differentially shapes dendrite morphology of medial entorhinal cortical ocean and island cells. Development 151:dev.202449. 10.1242/dev.202449 38856043 PMC11234379

[B163] Hamad M. I. K. Emerald B. S. Kumar K. K. Ibrahim M. F. Ali B. R. Bataineh M. F. (2023). Extracellular molecular signals shaping dendrite architecture during brain development. Front. Cell. Dev. Biol. 11:1254589. 10.3389/fcell.2023.1254589 38155836 PMC10754048

[B164] Hamad M. I. K. Jbara A. Rabaya O. Petrova P. Daoud S. Melliti N.. (2021a). Reelin signaling modulates GABAB receptor function in the neocortex. J. Neurochem. 156, 589–603. 10.1111/jnc.14990 32083308 PMC7442713

[B165] Hamad M. I. K. Petrova P. Daoud S. Rabaya O. Jbara A. Melliti N.. (2021b). Reelin restricts dendritic growth of interneurons in the neocortex. Development 148:dev199718. 10.1242/dev.199718 34414407 PMC8451942

[B166] Hamad M. I. K. Rabaya O. Jbara A. Daoud S. Petrova P. Ali B. R.. (2024b). Reelin regulates developmental desynchronization transition of neocortical network activity. Biomolecules 14:593. 10.3390/biom14050593 38786001 PMC11118507

[B167] Hanrahan J. Steeves K. L. Locke D. P. O'Brien T. M. Maekawa A. S. Amiri R.. (2024). Maternal exposure to polyethylene micro- and nanoplastics impairs umbilical blood flow but not fetal growth in pregnant mice. Sci. Rep. 14:399. 10.1038/s41598-023-50781-2 38172192 PMC10764924

[B168] Hao W. Wu J. Yuan N. Gong L. Huang J. Ma Q.. (2021). Xiaoyaosan improves antibiotic-induced depressive-like and anxiety-like behavior in mice through modulating the gut microbiota and regulating the NLRP3 inflammasome in the colon. Front. Pharmacol. 12:619103. 10.3389/fphar.2021.619103 33935710 PMC8087337

[B169] Haran J. P. Bhattarai S. K. Foley S. E. Dutta P. Ward D. V. Bucci V.. (2019). Alzheimer's disease microbiome is associated with dysregulation of the anti-inflammatory P-glycoprotein pathway. MBio 10, e00632–e00619. 10.1128/mBio.00632-19 31064831 PMC6509190

[B170] Harnett N. G. Goodman A. M. Knight D. C. (2020). PTSD-related neuroimaging abnormalities in brain function, structure, and biochemistry. Exp. Neurol. 330:113331. 10.1016/j.expneurol.2020.113331 32343956

[B171] Hashim H. Azmin S. Razlan H. Yahya N. W. Tan H. J. Manaf M. R. A.. (2014). Eradication of *Helicobacter pylori* infection improves levodopa action, clinical symptoms and quality of life in patients with Parkinson's disease. PLoS ONE 9:e112330. 10.1371/journal.pone.0112330 25411976 PMC4239049

[B172] Hashish S. Salama M. (2023). The role of an altered gut microbiome in Parkinson's disease: a narrative review. Appl. Microbiol. 3, 429–447. 10.3390/applmicrobiol3020030

[B173] Hatfield C. F. (2004). Disrupted daily activity/rest cycles in relation to daily cortisol rhythms of home-dwelling patients with early Alzheimer's dementia. Brain 127, 1061–1074. 10.1093/brain/awh129 14998915

[B174] Hawkins K. G. Casolaro C. Brown J. A. Edwards D. A. Wikswo J. P. (2020). The microbiome and the gut-liver-brain axis for central nervous system clinical pharmacology: challenges in specifying and integrating *in vitro* and *in silico* models. Clin. Pharma Therap. 108, 929–948. 10.1002/cpt.1870 32347548 PMC7572575

[B175] Hazan S. (2020). Rapid improvement in Alzheimer's disease symptoms following fecal microbiota transplantation: a case report. J. Int. Med. Res. 48:0300060520925930. 10.1177/0300060520925930 32600151 PMC7328362

[B176] He G. Cao Y. Ma H. Guo S. Xu W. Wang D.. (2023). Causal effects between gut microbiome and myalgic encephalomyelitis/chronic fatigue syndrome: a two-sample mendelian randomization study. Front. Microbiol. 14:1190894. 10.3389/fmicb.2023.1190894 37485509 PMC10359717

[B177] He Q. Wang W. Xu D. Xiong Y. Tao C. You C.. (2024). Potential causal association between gut microbiome and posttraumatic stress disorder. Transl. Psychiatry 14:67. 10.1038/s41398-024-02765-7 38296956 PMC10831060

[B178] Heath A.-L. M. Haszard J. J. Galland B. C. Lawley B. Rehrer N. J. Drummond L. N.. (2020). Association between the faecal short-chain fatty acid propionate and infant sleep. Eur. J. Clin. Nutr. 74, 1362–1365. 10.1038/s41430-019-0556-0 31969698

[B179] Hegde M. N. Freed D. B. Hegde M. N. Hegde M. N. (2022). Assessment of Communication Disorders in Adults: Resources and Protocols, 3rd Edn. San Diego, CA: Plural Publishing, Inc.

[B180] Heijtz R. D. Wang S. Anuar F. Qian Y. Björkholm B. Samuelsson A.. (2011). Normal gut microbiota modulates brain development and behavior. Proc. Natl. Acad. Sci. U.S.A. 108, 3047–3052. 10.1073/pnas.1010529108 21282636 PMC3041077

[B181] Heintz-Buschart A. Pandey U. Wicke T. Sixel-Döring F. Janzen A. Sittig-Wiegand E.. (2018). The nasal and gut microbiome in Parkinson's disease and idiopathic rapid eye movement sleep behavior disorder. Mov. Disord. 33, 88–98. 10.1002/mds.27105 28843021 PMC5811909

[B182] Hemmings S. M. J. Malan-Müller S. Van Den Heuvel L. L. Demmitt B. A. Stanislawski M. A. Smith D. G.. (2017). The microbiome in posttraumatic stress disorder and trauma-exposed controls: an exploratory study. Psychosom. Med. 79, 936–946. 10.1097/PSY.0000000000000512 28700459 PMC5763914

[B183] Herrera-Quintana L. Vázquez-Lorente H. Silva R. C. M. C. Olivares-Arancibia J. Reyes-Amigo T. Pires B. R. B.. (2024). The role of the microbiome and of radiotherapy-derived metabolites in breast cancer. Cancers 16:3671. 10.3390/cancers16213671 39518108 PMC11545256

[B184] Heun R. Maier W. (1993). The distinction of bipolar II disorder from bipolar I and recurrent unipolar depression: results of a controlled family study. Acta Psychiatr. Scand. 87, 279–284. 10.1111/j.1600-0447.1993.tb03372.x 8488750

[B185] Hey G. Nair N. Klann E. Gurrala A. Safarpour D. Mai V.. (2023). Therapies for Parkinson's disease and the gut microbiome: evidence for bidirectional connection. Front. Aging Neurosci. 15:1151850. 10.3389/fnagi.2023.1151850 37323145 PMC10261989

[B186] Honarpisheh P. Bryan R. M. McCullough L. D. (2022). Aging microbiota-gut-brain axis in stroke risk and outcome. Circ. Res. 130, 1112–1144. 10.1161/CIRCRESAHA.122.319983 35420913 PMC9674376

[B187] Honarpisheh P. Reynolds C. R. Blasco Conesa M. P. Moruno Manchon J. F. Putluri N. Bhattacharjee M. B.. (2020). Dysregulated gut homeostasis observed prior to the accumulation of the brain amyloid-β in Tg2576 mice. IJMS 21:1711. 10.3390/ijms21051711 32138161 PMC7084806

[B188] Hopfner F. Künstner A. Müller S. H. Künzel S. Zeuner K. E. Margraf N. G.. (2017). Gut microbiota in Parkinson disease in a northern German cohort. Brain Res. 1667, 41–45. 10.1016/j.brainres.2017.04.019 28506555

[B189] Horsager J. Andersen K. B. Knudsen K. Skjærbæk C. Fedorova T. D. Okkels N.. (2020). Brain-first versus body-first Parkinson's disease: a multimodal imaging case-control study. Brain 143, 3077–3088. 10.1093/brain/awaa238 32830221

[B190] Horsager J. Knudsen K. Borghammer P. (2021). Radionuclide imaging of the gut–brain axis in Parkinson disease. J. Nucl. Med. 62, 1504–1505. 10.2967/jnumed.121.262300 34301776 PMC8612321

[B191] Horsager J. Knudsen K. Sommerauer M. (2022). Clinical and imaging evidence of brain-first and body-first Parkinson's disease. Neurobiol. Dis. 164:105626. 10.1016/j.nbd.2022.105626 35031485

[B192] Houssini K. Li J. Tan Q. (2025). Complexities of the global plastics supply chain revealed in a trade-linked material flow analysis. Commun. Earth Environ. 6:257. 10.1038/s43247-025-02169-5

[B193] Hsiao C. C. Yang A.-M. Wang C. Lin C.-Y. (2023). Association between glyphosate exposure and cognitive function, depression, and neurological diseases in a representative sample of US adults: NHANES 2013–2014 analysis. Environ. Res. 237;116860. 10.1016/j.envres.2023.116860 37562738

[B194] Hsu C.-Y. Ahmad I. Maya R. W. Abass M. A. Gupta J. Singh A.. (2025). The potential therapeutic approaches targeting gut health in myalgic encephalomyelitis/chronic fatigue syndrome (ME/CFS): a narrative review. J. Transl. Med. 23:530. 10.1186/s12967-025-06527-x 40350437 PMC12066075

[B195] Hu J. Jiao W. Tang Z. Wang C. Li Q. Wei M.. (2023). Quercetin inclusion complex gels ameliorate radiation-induced brain injury by regulating gut microbiota. Biomed. Pharmacother. 158:114142. 10.1016/j.biopha.2022.114142 36527844

[B196] Hu J. Li H. Wang X. Cheng H. Zhu G. Yang S. (2024). Novel mechanisms of Anshen Dingzhi prescription against PTSD: Inhibiting DCC to modulate synaptic function and inflammatory responses. J. Ethnopharmacol. 333, 118425. 10.1016/j.jep.2024.118425 38848974

[B197] Hu S. Li A. Huang T. Lai J. Li J. Sublette M. E.. (2019). Gut microbiota changes in patients with bipolar depression. Adv. Sci. 6:1900752. 10.1002/advs.201900752 31380217 PMC6662053

[B198] Hu W. Kong X. Wang H. Li Y. Luo Y. (2022). Ischemic stroke and intestinal flora: an insight into brain-gut axis. Eur. J. Med. Res. 27:73. 10.1186/s40001-022-00691-2 35614480 PMC9131669

[B199] Huang H. Hou J. Li M. Wei F. Liao Y. Xi B. (2025). Microplastics in the bloodstream can induce cerebral thrombosis by causing cell obstruction and lead to neurobehavioral abnormalities. Sci. Adv. 11:eadr8243. 10.1126/sciadv.adr8243 39841831 PMC11753373

[B200] Huang H. Xu H. Luo Q. He J. Li M. Chen H.. (2019). Fecal microbiota transplantation to treat Parkinson's disease with constipation: a case report. Medicine 98:e16163. 10.1097/MD.0000000000016163 31261545 PMC6616439

[B201] Huang Y. (2012). Effects of age and amyloid deposition on Aβ dynamics in the human central nervous system. Arch. Neurol. 69:51. 10.1001/archneurol.2011.235 21911660 PMC3254706

[B202] Huang Y. Shi X. Li Z. Shen Y. Shi X. Wang L.. (2018). Possible association of Firmicutes in the gut microbiota of patients with major depressive disorder. NDT 14, 3329–3337. 10.2147/NDT.S188340 30584306 PMC6284853

[B203] Huang Y.-Y. Gan Y.-H. Yang L. Cheng W. Yu J.-T. (2024). Depression in Alzheimer's disease: epidemiology, mechanisms, and treatment. Biol. Psychiatry 95, 992–1005. 10.1016/j.biopsych.2023.10.008 37866486

[B204] Huckins L. M. Chatzinakos C. Breen M. S. Hartmann J. Klengel T. Da Silva Almeida A. C.. (2020). Analysis of genetically regulated gene expression identifies a prefrontal PTSD Gene, SNRNP35, specific to military cohorts. Cell Rep. 31:107716. 10.1016/j.celrep.2020.107716 32492425 PMC7359754

[B205] Ibrahim A. Ali R. A. R. Manaf M. R. A. Ahmad N. Tajurruddin F. W. Qin W. Z.. (2020). Multi-strain probiotics (Hexbio) containing MCP BCMC strains improved constipation and gut motility in Parkinson's disease: a randomised controlled trial. PLoS ONE 15:e0244680. 10.1371/journal.pone.0244680 33382780 PMC7774928

[B206] Iqbal N. T. Khan H. Khalid A. Mahmood S. F. Nasir N. Khanum I.. (2025). Chronic inflammation in post-acute sequelae of COVID-19 modulates gut microbiome: a review of literature on COVID-19 sequelae and gut dysbiosis. Mol. Med. 31:22. 10.1186/s10020-024-00986-6 39849406 PMC11756069

[B207] Jackson A. Forsyth C. B. Shaikh M. Voigt R. M. Engen P. A. Ramirez V.. (2019). Diet in Parkinson's disease: critical role for the microbiome. Front. Neurol. 10:1245. 10.3389/fneur.2019.01245 31920905 PMC6915094

[B208] Jaggar M. Rea K. Spichak S. Dinan T. G. Cryan J. F. (2020). You've got male: Sex and the microbiota-gut-brain axis across the lifespan. Front. Neuroendocrinol. 56:100815. 10.1016/j.yfrne.2019.100815 31805290

[B209] Jak A. J. Jurick S. Hoffman S. Evangelista N. D. Deford N. Keller A.. (2020). PTSD, but not history of mTBI, is associated with altered myelin in combat-exposed Iraq and Afghanistan Veterans. Clin. Neuropsychol. 34, 1070–1087. 10.1080/13854046.2020.1730975 32176590

[B210] Jangi S. Gandhi R. Cox L. M. Li N. Von Glehn F. Yan R.. (2016). Alterations of the human gut microbiome in multiple sclerosis. Nat. Commun. 7:12015. 10.1038/ncomms12015 27352007 PMC4931233

[B211] Jarrett J. T. Berger E. P. Lansbury P. T. (1993). The carboxy terminus of the.beta. amyloid protein is critical for the seeding of amyloid formation: Implications for the pathogenesis of Alzheimer's disease. Biochemistry 32, 4693–4697. 10.1021/bi00069a001 8490014

[B212] Javaid S. F. Hashim I. J. Hashim M. J. Stip E. Samad M. A. Ahbabi A. A. (2023). Epidemiology of anxiety disorders: global burden and sociodemographic associations. Middle East Curr. Psychiatry 30:44. 10.1186/s43045-023-00315-3

[B213] Jenkins T. Nguyen J. Polglaze K. Bertrand P. (2016). Influence of tryptophan and serotonin on mood and cognition with a possible role of the gut-brain axis. Nutrients 8:56. 10.3390/nu8010056 26805875 PMC4728667

[B214] Jergović M. Bendelja K. Vidović A. Savić A. Vojvoda V. Aberle N.. (2014). Patients with posttraumatic stress disorder exhibit an altered phenotype of regulatory T cells. All. Asth. Clin. Immun. 10:43. 10.1186/1710-1492-10-43 25670936 PMC4322511

[B215] Jia L. Xiao L. Fu Y. Shao Z. Jing Z. Yuan J.. (2024). Neuroprotective effects of probiotics on anxiety- and depression-like disorders in stressed mice by modulating tryptophan metabolism and the gut microbiota. Food Funct. 15, 2895–2905. 10.1039/D3FO03897A 38404190

[B216] Jiang H. Ling Z. Zhang Y. Mao H. Ma Z. Yin Y.. (2015). Altered fecal microbiota composition in patients with major depressive disorder. Brain Behav. Immun. 48, 186–194. 10.1016/j.bbi.2015.03.016 25882912

[B217] Jiang H. Zhang X. Yu Z. Zhang Z. Deng M. Zhao J.. (2018). Altered gut microbiota profile in patients with generalized anxiety disorder. J. Psychiatr. Res. 104, 130–136. 10.1016/j.jpsychires.2018.07.007 30029052

[B218] Jiang W. Hu C. Chen Y. Li Y. Sun X. Wu H.. (2023). Dysregulation of the microbiota-brain axis during long-term exposure to polystyrene nanoplastics in rats and the protective role of dihydrocaffeic acid. Sci. Total Environ. 874:162101. 10.1016/j.scitotenv.2023.162101 36764550

[B219] Jiménez-Arroyo C. Tamargo A. Molinero N. Reinosa J. J. Alcolea-Rodriguez V. Portela R.. (2023). Simulated gastrointestinal digestion of polylactic acid (PLA) biodegradable microplastics and their interaction with the gut microbiota. Sci. Total Environ. 902:166003. 10.1016/j.scitotenv.2023.166003 37549707

[B220] Jin Y. Chi J. LoMonaco K. Boon A. Gu H. (2023). Recent review on selected xenobiotics and their impacts on gut microbiome and metabolome. Trends Anal. Chem. 166:117155. 10.1016/j.trac.2023.117155 37484879 PMC10361410

[B221] Joe E. Ringman J. M. (2019). Cognitive symptoms of Alzheimer's disease: clinical management and prevention. BMJ 367:l6217. 10.1136/bmj.l6217 31810978

[B222] Johnstone N. Milesi C. Burn O. Van Den Bogert B. Nauta A. Hart K.. (2021). Anxiolytic effects of a galacto-oligosaccharides prebiotic in healthy females (18–25 years) with corresponding changes in gut bacterial composition. Sci. Rep. 11:8302. 10.1038/s41598-021-87865-w 33859330 PMC8050281

[B223] Joo M.-K. Shin Y.-J. Kim D.-H. (2023). Cefaclor causes vagus nerve-mediated depression-like symptoms with gut dysbiosis in mice. Sci. Rep. 13:15529. 10.1038/s41598-023-42690-1 37726354 PMC10509198

[B224] Jurek J. M. Castro-Marrero J. (2024). A narrative review on gut microbiome disturbances and microbial preparations in myalgic encephalomyelitis/chronic fatigue syndrome: implications for long COVID. Nutrients 16:1545. 10.3390/nu16111545 38892479 PMC11173566

[B225] Kang J.-E. Lim M. M. Bateman R. J. Lee J. J. Smyth L. P. Cirrito J. R.. (2009). Amyloid-β dynamics are regulated by orexin and the sleep-wake cycle. Science 326, 1005–1007. 10.1126/science.1180962 19779148 PMC2789838

[B226] Kang J. W. Zivkovic A. M. (2021). The potential utility of prebiotics to modulate Alzheimer's disease: a review of the evidence. Microorganisms 9:2310. 10.3390/microorganisms9112310 34835436 PMC8625457

[B227] Kao A. C.-C. Spitzer S. Anthony D. C. Lennox B. Burnet P. W. J. (2018). Prebiotic attenuation of olanzapine-induced weight gain in rats: analysis of central and peripheral biomarkers and gut microbiota. Transl. Psychiatry 8:66. 10.1038/s41398-018-0116-8 29540664 PMC5852210

[B228] Kasarello K. Cudnoch-Jedrzejewska A. Czarzasta K. (2023). Communication of gut microbiota and brain via immune and neuroendocrine signaling. Front. Microbiol. 14:1118529. 10.3389/fmicb.2023.1118529 36760508 PMC9907780

[B229] Katrinli S. Oliveira N. C. S. Felger J. C. Michopoulos V. Smith A. K. (2022). The role of the immune system in posttraumatic stress disorder. Transl. Psychiatry 12:313. 10.1038/s41398-022-02094-7 35927237 PMC9352784

[B230] Kazemi A. Noorbala A. A. Azam K. Eskandari M. H. Djafarian K. (2019). Effect of probiotic and prebiotic vs placebo on psychological outcomes in patients with major depressive disorder: a randomized clinical trial. Clin. Nutr. 38, 522–528. 10.1016/j.clnu.2018.04.010 29731182

[B231] Ke S. Wang X.-W. Ratanatharathorn A. Huang T. Roberts A. L. Grodstein F.. (2023). Association of probable post-traumatic stress disorder with dietary pattern and gut microbiome in a cohort of women. Nat. Ment. Health 1, 900–913. 10.1038/s44220-023-00145-6 PMC13195745 42183083

[B232] Kelmendi B. Adams T. G. Yarnell S. Southwick S. Abdallah C. G. Krystal J. H. (2016). PTSD: from neurobiology to pharmacological treatments. Eur. J. Psychotraumatol. 7:31858. 10.3402/ejpt.v7.31858 27837583 PMC5106865

[B233] Kent K. G. (2024). The relationship between post-traumatic stress disorder and gastrointestinal disease in United States Military Veterans. SAGE Open Med. 12:20503121241260000. 10.1177/20503121241260000 38911441 PMC11193927

[B234] Keshavarzian A. Green S. J. Engen P. A. Voigt R. M. Naqib A. Forsyth C. B.. (2015). Colonic bacterial composition in Parkinson's disease. Mov. Disord. 30, 1351–1360. 10.1002/mds.26307 26179554

[B235] Kesika P. Suganthy N. Sivamaruthi B. S. Chaiyasut C. (2021). Role of gut-brain axis, gut microbial composition, and probiotic intervention in Alzheimer's disease. Life Sci. 264:118627. 10.1016/j.lfs.2020.118627 33169684

[B236] Khan M. F. Khodve G. Yadav S. Mallick K. Banerjee S. (2025). Probiotic treatment improves post-traumatic stress disorder outcomes in mice. Behav. Brain Res. 476:115246. 10.1016/j.bbr.2024.115246 39255901

[B237] Kiecka A. Szczepanik M. (2023). Proton pump inhibitor-induced gut dysbiosis and immunomodulation: current knowledge and potential restoration by probiotics. Pharmacol. Rep. 75, 791–804. 10.1007/s43440-023-00489-x 37142877 PMC10159235

[B238] Kim H. Kim S. Park S. Park G. Shin H. Park M. S.. (2021). Administration of *Bifidobacterium bifidum* BGN4 and *Bifidobacterium longum* BORI improves cognitive and memory function in the mouse model of Alzheimer's disease. Front. Aging Neurosci. 13:709091. 10.3389/fnagi.2021.709091 34421576 PMC8378450

[B239] Kim N. Jeon S. H. Ju I. G. Gee M. S. Do J. Oh M. S.. (2021). Transplantation of gut microbiota derived from Alzheimer's disease mouse model impairs memory function and neurogenesis in C57BL/6 mice. Brain Behav. Immun. 98, 357–365. 10.1016/j.bbi.2021.09.002 34500036

[B240] Kim S. Kwon S.-H. Kam T.-I. Panicker N. Karuppagounder S. S. Lee S.. (2019). Transneuronal propagation of pathologic α-synuclein from the gut to the brain models Parkinson's disease. Neuron 103, 627–641.e7. 10.1016/j.neuron.2019.05.035 31255487 PMC6706297

[B241] Kim S.-Y. Woo S.-Y. Raza S. Ho D. Jeon S. W. Chang Y.. (2023). Association between gut microbiota and anxiety symptoms: A large population-based study examining sex differences. J. Affect. Disord. 333, 21–29. 10.1016/j.jad.2023.04.003 37031878

[B242] Kiriyama Y. Nochi H. (2019). The biosynthesis, signaling, and neurological functions of bile acids. Biomolecules 9:232. 10.3390/biom9060232 31208099 PMC6628048

[B243] Kitami T. Fukuda S. Kato T. Yamaguti K. Nakatomi Y. Yamano E.. (2020). Deep phenotyping of myalgic encephalomyelitis/chronic fatigue syndrome in Japanese population. Sci. Rep. 10:19933. 10.1038/s41598-020-77105-y 33199820 PMC7669873

[B244] Knudsen J. K. Bundgaard-Nielsen C. Hjerrild S. Nielsen R. E. Leutscher P. Sørensen S. (2021). Gut microbiota variations in patients diagnosed with major depressive disorder—A systematic review. Brain Behav. 11:e02177. 10.1002/brb3.2177 34047485 PMC8323045

[B245] Kobayashi Y. Sugahara H. Shimada K. Mitsuyama E. Kuhara T. Yasuoka A.. (2017). Therapeutic potential of *Bifidobacterium breve* strain A1 for preventing cognitive impairment in Alzheimer's disease. Sci. Rep. 7:13510. 10.1038/s41598-017-13368-2 29044140 PMC5647431

[B246] König R. S. Albrich W. C. Kahlert C. R. Bahr L. S. Löber U. Vernazza P.. (2021). The gut microbiome in myalgic encephalomyelitis (ME)/chronic fatigue syndrome (CFS). Front. Immunol. 12:628741. 10.3389/fimmu.2021.628741 35046929 PMC8761622

[B247] Krishnan M. Mydeen A. B. Nakhal M. M. Ibrahim M. F. Jayaraj R. L. Ljubisavljevic M. R.. (2025). Altered dendritic morphology of MEC II pyramidal and stellate cells in Rett syndrome mice. Front. Neuroanat. 19:1580435. 10.3389/fnana.2025.1580435 40630552 PMC12236101

[B248] Kuhlmann T. Ludwin S. Prat A. Antel J. Brück W. Lassmann H. (2017). An updated histological classification system for multiple sclerosis lesions. Acta Neuropathol. 133, 13–24. 10.1007/s00401-016-1653-y 27988845

[B249] Kwan J. Vullaganti M. (2022). Amyotrophic lateral sclerosis mimics. Muscle Nerve 66, 240–252. 10.1002/mus.27567 35607838

[B250] Laaker C. Hsu M. Fabry Z. Miller S. D. Karpus W. J. (2021). Experimental autoimmune encephalomyelitis in the mouse. Curr. Protoc. 1:e300. 10.1002/cpz1.300 34870897

[B251] Lai Y. Xiong P. (2025). Analysis of gut microbiota and depression and anxiety: Mendelian randomization from three datasets. Gen. Hosp. Psychiatry 94, 206–218. 10.1016/j.genhosppsych.2025.03.012 40154232

[B252] Latifi S. Tamayol A. Habibey R. Sabzevari R. Kahn C. Geny D.. (2016). Natural lecithin promotes neural network complexity and activity. Sci. Rep. 6:25777. 10.1038/srep25777 27228907 PMC4882550

[B253] Laudani S. Torrisi S. A. Alboni S. Bastiaanssen T. F. S. Benatti C. Rivi V.. (2023). Gut microbiota alterations promote traumatic stress susceptibility associated with p-cresol-induced dopaminergic dysfunctions. Brain Behav. Immun. 107, 385–396. 10.1016/j.bbi.2022.11.004 36400332

[B254] Lavasani S. Dzhambazov B. Nouri M. Fåk F. Buske S. Molin G.. (2010). A novel probiotic mixture exerts a therapeutic effect on experimental autoimmune encephalomyelitis mediated by IL-10 producing regulatory T cells. PLoS ONE 5:e9009. 10.1371/journal.pone.0009009 20126401 PMC2814855

[B255] Lavebratt C. Yang L. L. Giacobini M. Forsell Y. Schalling M. Partonen T.. (2019). Early exposure to antibiotic drugs and risk for psychiatric disorders: a population-based study. Transl. Psychiatry 9:317. 10.1038/s41398-019-0653-9 31772217 PMC6879739

[B256] Leclercq S. Forsythe P. Bienenstock J. (2016). Posttraumatic stress disorder: does the gut microbiome hold the key? Can. J. Psychiatry 61, 204–213. 10.1177/0706743716635535 27254412 PMC4794957

[B257] Lee B. Kwon J.-T. Jeong Y. Caris H. Oh D. Feng M.. (2025). Inflammatory and anti-inflammatory cytokines bidirectionally modulate amygdala circuits regulating anxiety. Cell 188, 2190–2202.e15. 10.1016/j.cell.2025.03.005 40199321 PMC12090750

[B258] Lee H.-J. Lee K.-E. Kim J.-K. Kim D.-H. (2019b). Suppression of gut dysbiosis by *Bifidobacterium longum* alleviates cognitive decline in 5XFAD transgenic and aged mice. Sci. Rep. 9:11814. 10.1038/s41598-019-48342-7 31413350 PMC6694197

[B259] Lee H. J. Hong J. K. Kim J.-K. Kim D.-H. Jang S. W. Han S.-W.. (2021). Effects of probiotic NVP-1704 on mental health and sleep in healthy adults: an 8-week randomized, double-blind, placebo-controlled trial. Nutrients 13:2660. 10.3390/nu13082660 34444820 PMC8398773

[B260] Lee H. J. Son Y. Lee M. Moon C. Kim S. H. Shin I. S.. (2019a). Sodium butyrate prevents radiation-induced cognitive impairment by restoring pCREB/BDNF expression. Neural. Regen. Res. 14, 1530–1535. 10.4103/1673-5374.255974 31089051 PMC6557090

[B261] Lee J. Park S. J. Choi S. Chang J. Park Y. J. Jeong S.. (2024). Antibiotic exposure and depression incidence: a cohort study of the Korean population. Psychiatry Res. 339:115992. 10.1016/j.psychres.2024.115992 38875919

[B262] Lee S.-H. Park S.-Y. Choi C. S. (2022). Insulin resistance: from mechanisms to therapeutic strategies. Diabetes Metab. J. 46, 15–37. 10.4093/dmj.2021.0280 34965646 PMC8831809

[B263] Lee Y. T. Mohd Ismail N. I. Wei L. K. (2021). Microbiome and ischemic stroke: a systematic review. PLoS ONE 16:e0245038. 10.1371/journal.pone.0245038 33439913 PMC7806160

[B264] Lehman P. C. Cady N. Ghimire S. Shahi S. K. Shrode R. L. Lehmler H.-J.. (2023). Low-dose glyphosate exposure alters gut microbiota composition and modulates gut homeostasis. Environ. Toxicol. Pharmacol. 100:104149. 10.1016/j.etap.2023.104149 37196884 PMC10330715

[B265] Leifeld J. Förster E. Reiss G. Hamad M. I. K. (2022). Considering the role of extracellular matrix molecules, in particular reelin, in granule cell dispersion related to temporal lobe epilepsy. Front. Cell. Dev. Biol. 10:917575. 10.3389/fcell.2022.917575 35733853 PMC9207388

[B266] Leino L. Tall T. Helander M. Saloniemi I. Saikkonen K. Ruuskanen S.. (2021). Classification of the glyphosate target enzyme (5-enolpyruvylshikimate-3-phosphate synthase) for assessing sensitivity of organisms to the herbicide. J. Hazard. Mater. 408:124556. 10.1016/j.jhazmat.2020.124556 33243645

[B267] Li B. He Y. Ma J. Huang P. Du J. Cao L.. (2019). Mild cognitive impairment has similar alterations as Alzheimer's disease in gut microbiota. Alzheimers. Dement. 15, 1357–1366. 10.1016/j.jalz.2019.07.002 31434623

[B268] Li G. Liu X. Sun X. Huang L. Kuang W. Ou J.. (2024). Polystyrene microplastics induce anxiety via HRAS derived PERK-NF-κB pathway. Environ. Int. 185:108543. 10.1016/j.envint.2024.108543 38452464

[B269] Li N. Xiao X. Zhang H. Bai Z. Li M. Sun J.. (2024). Sterile soil mitigates the intergenerational loss of gut microbial diversity and anxiety-like behavior induced by antibiotics in mice. Brain Behav. Immun. 115, 179–190. 10.1016/j.bbi.2023.10.014 37848098

[B270] Li Q. Meng L. Chen L. Shi X. Tu L. Zhou Q.. (2023). The role of the microbiota-gut-brain axis and intestinal microbiome dysregulation in Parkinson's disease. Front. Neurol. 14:1185375. 10.3389/fneur.2023.1185375 37305758 PMC10249504

[B271] Li X. Liu S. Wang F. Li X. Liu H. Lian T.. (2025). Dietary herbs that interact with gut microbiota: roles as anti-stroke agents. Food Sci. Biotechnol. 34, 547–562. 10.1007/s10068-024-01698-7 39958164 PMC11822190

[B272] Li Y. I. Pagulayan K. Rau H. Hendrickson R. Schindler A. G. (2025). Gut microbial composition is associated with symptom self-report in trauma-exposed Iraq and Afghanistan veterans. Neurotrauma Rep. 6, 1–12. 10.1089/neur.2024.0011 40012717 PMC11850977

[B273] Li Z. Ke X. Zuo D. Wang Z. Fang F. Li B. (2022). New insights into the relationship between gut microbiota and radiotherapy for cancer. Nutrients 15:48. 10.3390/nu15010048 36615706 PMC9824372

[B274] Lian H. Yang L. Cole A. Sun L. Chiang A. C.-A. Fowler S. W.. (2015). NFκB-activated astroglial release of complement C3 compromises neuronal morphology and function associated with Alzheimer's disease. Neuron 85, 101–115. 10.1016/j.neuron.2014.11.018 25533482 PMC4289109

[B275] Liang S. Zhao L. Ni P. Wang Q. Guo W. Xu Y.. (2024). Frontostriatal circuitry and the tryptophan kynurenine pathway in major psychiatric disorders. Psychopharmacology 241, 97–107. 10.1007/s00213-023-06466-9 37735237

[B276] Liddelow S. A. Guttenplan K. A. Clarke L. E. Bennett F. C. Bohlen C. J. Schirmer L.. (2017). Neurotoxic reactive astrocytes are induced by activated microglia. Nature 541, 481–487. 10.1038/nature21029 28099414 PMC5404890

[B277] Lim A. S. P. Kowgier M. Yu L. Buchman A. S. Bennett D. A. (2013). Sleep fragmentation and the risk of incident Alzheimer's disease and cognitive decline in older persons. Sleep 36, 1027–1032. 10.5665/sleep.2802 23814339 PMC3669060

[B278] Lin A. Zheng W. He Y. Tang W. Wei X. He R.. (2018). Gut microbiota in patients with Parkinson's disease in southern China. Parkinson. Relat. Disord. 53, 82–88. 10.1016/j.parkreldis.2018.05.007 29776865

[B279] Lindell A. E. Zimmermann-Kogadeeva M. Patil K. R. (2022). Multimodal interactions of drugs, natural compounds and pollutants with the gut microbiota. Nat. Rev. Microbiol. 20, 431–443. 10.1038/s41579-022-00681-5 35102308 PMC7615390

[B280] Liu J. Liu C. Yue J. (2021). Radiotherapy and the gut microbiome: facts and fiction. Radiation Oncol. 16:9. 10.1186/s13014-020-01735-9 33436010 PMC7805150

[B281] Liu J. Ning W. Zhang N. Zhu B. Mao Y. (2024). Estimation of the global disease burden of depression and anxiety between 1990 and 2044: an analysis of the global burden of disease study 2019. Healthcare 12:1721. 10.3390/healthcare12171721 39273745 PMC11395616

[B282] Liu J. Wang F. (2017). Role of neuroinflammation in amyotrophic lateral sclerosis: cellular mechanisms and therapeutic implications. Front. Immunol. 8:1005. 10.3389/fimmu.2017.01005 28871262 PMC5567007

[B283] Liu J. Wang F. Liu S. Du J. Hu X. Xiong J.. (2017). Sodium butyrate exerts protective effect against Parkinson's disease in mice via stimulation of glucagon like peptide-1. J. Neurol. Sci. 381, 176–181. 10.1016/j.jns.2017.08.3235 28991675

[B284] Liu P. Wu L. Peng G. Han Y. Tang R. Ge J.. (2019). Altered microbiomes distinguish Alzheimer's disease from amnestic mild cognitive impairment and health in a Chinese cohort. Brain Behav. Immun. 80, 633–643. 10.1016/j.bbi.2019.05.008 31063846

[B285] Liu R. T. Rowan-Nash A. D. Sheehan A. E. Walsh R. F. L. Sanzari C. M. Korry B. J.. (2020). Reductions in anti-inflammatory gut bacteria are associated with depression in a sample of young adults. Brain Behav. Immun. 88, 308–324. 10.1016/j.bbi.2020.03.026 32229219 PMC7415740

[B286] Liu T. Sun W. Guo S. Chen T. Zhu M. Yuan Z.. (2024). Research progress on pathogenesis of chronic fatigue syndrome and treatment of traditional Chinese and Western medicine. Auton. Neurosci. 255:103198. 10.1016/j.autneu.2024.103198 39047501

[B287] Liu X. Li Y. Gu M. Xu T. Wang C. Chang P. (2024). Radiation enteropathy-related depression: a neglectable course of disease by gut bacterial dysbiosis. Cancer Med. 13:e6865. 10.1002/cam4.6865 38457257 PMC10923036

[B288] Liu Y. Forsythe P. (2021). Vagotomy and insights into the microbiota-gut-brain axis. Neurosci. Res. 168, 20–27. 10.1016/j.neures.2021.04.001 33887355

[B289] Liu Y. Wang Y. Ni Y. Cheung C. K. Y. Lam K. S. L. Wang Y.. (2020). Gut microbiome fermentation determines the efficacy of exercise for diabetes prevention. Cell Metab. 31, 77–91.e5. 10.1016/j.cmet.2019.11.001 31786155

[B290] Liu Z. Wei S. Chen X. Liu L. Wei Z. Liao Z.. (2022). The effect of long-term or repeated use of antibiotics in children and adolescents on cognitive impairment in middle-aged and older person(s) adults: a cohort study. Front. Aging Neurosci. 14:833365. 10.3389/fnagi.2022.833365 35401157 PMC8984107

[B291] Loh J. S. Mak W. Q. Tan L. K. S. Ng C. X. Chan H. H. Yeow S. H.. (2024). Microbiota-gut-brain axis and its therapeutic applications in neurodegenerative diseases. Sig. Transduct. Target. Ther. 9:37. 10.1038/s41392-024-01743-1 38360862 PMC10869798

[B292] Lourida I. Soni M. Thompson-Coon J. Purandare N. Lang I. A. Ukoumunne O. C.. (2013). Mediterranean diet, cognitive function, and dementia: a systematic review. Epidemiology 24, 479–489. 10.1097/EDE.0b013e3182944410 23680940

[B293] Lu L. Li F. Gao Y. Kang S. Li J. Guo J. (2024). Microbiome in radiotherapy: an emerging approach to enhance treatment efficacy and reduce tissue injury. Mol. Med. 30:105. 10.1186/s10020-024-00873-0 39030525 PMC11264922

[B294] Lu L. Li W. Sun C. Kang S. Li J. Luo X.. (2020). Phycocyanin ameliorates radiation-induced acute intestinal toxicity by regulating the effect of the gut microbiota on the TLR4/Myd88/NF-κB pathway. J. Parenteral Enteral Nutr. 44, 1308–1317. 10.1002/jpen.1744 31769063

[B295] Lubomski M. Davis R. L. Sue C. M. (2020). Gastrointestinal dysfunction in Parkinson's disease. J. Neurol. 267, 1377–1388. 10.1007/s00415-020-09723-5 31989280

[B296] Lubomski M. Xu X. Holmes A. J. Muller S. Yang J. Y. H. Davis R. L.. (2022a). The gut microbiome in Parkinson's disease: a longitudinal study of the impacts on disease progression and the use of device-assisted therapies. Front. Aging Neurosci. 14:875261. 10.3389/fnagi.2022.875261 35656540 PMC9152137

[B297] Lubomski M. Xu X. Holmes A. J. Yang J. Y. H. Sue C. M. Davis R. L. (2022b). The impact of device-assisted therapies on the gut microbiome in Parkinson's disease. J. Neurol. 269, 780–795. 10.1007/s00415-021-10657-9 34128115

[B298] Luo J. Lin S. (2025). Association between microplastics exposure and depressive symptoms in college students. Ecotoxicol. Environ. Saf. 295:118142. 10.1016/j.ecoenv.2025.118142 40185030

[B299] Luo N. Zhu W. Li X. Fu M. Peng X. Yang F.. (2022). Impact of gut microbiota on radiation-associated cognitive dysfunction and neuroinflammation in mice. Radiat. Res. 197, 350–364. 10.1667/RADE-21-00006.1 34982167

[B300] Lupo G. F. D. Rocchetti G. Lucini L. Lorusso L. Manara E. Bertelli M.. (2021). Potential role of microbiome in chronic fatigue syndrome/myalgic encephalomyelits (CFS/ME). Sci. Rep. 11:7043. 10.1038/s41598-021-86425-6 33782445 PMC8007739

[B301] Lyte M. Varcoe J. J. Bailey M. T. (1998). Anxiogenic effect of subclinical bacterial infection in mice in the absence of overt immune activation. Physiol. Behav. 65, 63–68. 10.1016/S0031-9384(98)00145-0 9811366

[B302] Ma C. Hong F. Yang S. (2022). Amyloidosis in Alzheimer's disease: pathogeny, etiology, and related therapeutic directions. Molecules 27:1210. 10.3390/molecules27041210 35209007 PMC8876037

[B303] Mac Giollabhui N. Slaney C. Hemani G. Foley É. M. Van Der Most P. J. Nolte I. M.. (2025). Role of inflammation in depressive and anxiety disorders, affect, and cognition: genetic and non-genetic findings in the lifelines cohort study. Transl. Psychiatry 15:164. 10.1038/s41398-025-03372-w 40348744 PMC12065825

[B304] Makkawi S. Camara-Lemarroy C. Metz L. (2018). Fecal microbiota transplantation associated with 10 years of stability in a patient with SPMS. Neurol. Neuroimmunol. Neuroinflamm. 5:e459. 10.1212/NXI.0000000000000459 29619403 PMC5882466

[B305] Malan-Muller S. Valles-Colomer M. Foxx C. L. Vieira-Silva S. Van Den Heuvel L. L. Raes J.. (2022). Exploring the relationship between the gut microbiome and mental health outcomes in a posttraumatic stress disorder cohort relative to trauma-exposed controls. Europ. Neuropsychopharmacol. 56, 24–38. 10.1016/j.euroneuro.2021.11.009 34923209

[B306] Malhi G. S. Mann J. J. (2018). Depression. Lancet 392, 2299–2312. 10.1016/S0140-6736(18)31948-2 30396512

[B307] Manda K. Glasow A. Paape D. Hildebrandt G. (2012). Effects of ionizing radiation on the immune system with special emphasis on the interaction of dendritic and T cells. Front. Oncol. 2:102. 10.3389/fonc.2012.00102 22937525 PMC3426842

[B308] Margolis K. G. Cryan J. F. Mayer E. A. (2021). The microbiota-gut-brain axis: from motility to mood. Gastroenterology 160, 1486–1501. 10.1053/j.gastro.2020.10.066 33493503 PMC8634751

[B309] Marx W. Penninx B. W. J. H. Solmi M. Furukawa T. A. Firth J. Carvalho A. F.. (2023). Major depressive disorder. Nat. Rev. Dis. Primers 9:44. 10.1038/s41572-023-00454-1 37620370

[B310] Matei B. Winters-Stone K. M. Raber J. (2023). Examining the mechanisms behind exercise's multifaceted impacts on body composition, cognition, and the gut microbiome in cancer survivors: exploring the links to oxidative stress and inflammation. Antioxidants 12:1423. 10.3390/antiox12071423 37507961 PMC10376047

[B311] Matsumoto M. Inoue R. Tsukahara T. Ushida K. Chiji H. Matsubara N.. (2008). Voluntary running exercise alters microbiota composition and increases n-butyrate concentration in the rat cecum. Biosci. Biotechnol. Biochem. 72, 572–576. 10.1271/bbb.70474 18256465

[B312] McEwen B. S. Akil H. (2020). Revisiting the stress concept: implications for affective disorders. J. Neurosci. 40, 12–21. 10.1523/JNEUROSCI.0733-19.2019 31896560 PMC6939488

[B313] McIntyre R. S. Subramaniapillai M. Shekotikhina M. Carmona N. E. Lee Y. Mansur R. B.. (2021). Characterizing the gut microbiota in adults with bipolar disorder: a pilot study. Nutr. Neurosci. 24, 173–180. 10.1080/1028415X.2019.1612555 31132957

[B314] Meier S. M. Trontti K. Purves K. L. Als T. D. Grove J. Laine M.. (2019). Genetic variants associated with anxiety and stress-related disorders: a genome-wide association study and mouse-model study. JAMA Psychiatry 76:924. 10.1001/jamapsychiatry.2019.1119 31116379 PMC6537792

[B315] Melis M. Vascellari S. Santoru M. L. Oppo V. Fabbri M. Sarchioto M.. (2021). Gut microbiota and metabolome distinctive features in Parkinson disease: Focus on levodopa and levodopa-carbidopa intrajejunal gel. Euro. J. Neurol. 28, 1198–1209. 10.1111/ene.14644 33185912

[B316] Meng Q. Lin M.-S. Tzeng I.-S. (2020). Relationship between exercise and Alzheimer's disease: a narrative literature review. Front. Neurosci. 14:131. 10.3389/fnins.2020.00131 32273835 PMC7113559

[B317] Menozzi E. Schapira A. H. V. (2024). The gut microbiota in Parkinson disease: interactions with drugs and potential for therapeutic applications. CNS Drugs 38, 315–331. 10.1007/s40263-024-01073-4 38570412 PMC11026199

[B318] Mesnage R. Teixeira M. Mandrioli D. Falcioni L. Ducarmon Q. R. Zwittink R. D.. (2021). Use of shotgun metagenomics and metabolomics to evaluate the impact of glyphosate or roundup MON 52276 on the gut microbiota and serum metabolome of sprague-dawley rats. Environ. Health Perspect. 129:017005. 10.1289/EHP6990 33502259 PMC7839352

[B319] Mestre L. Carrillo-Salinas F. J. Mecha M. Feliú A. Espejo C. Álvarez-Cermeño J. C.. (2019). Manipulation of gut microbiota influences immune responses, axon preservation, and motor disability in a model of progressive multiple sclerosis. Front. Immunol. 10:1374. 10.3389/fimmu.2019.01374 31258540 PMC6587398

[B320] Metaxas A. Kempf S. (2016). Neurofibrillary tangles in Alzheimer′s disease: elucidation of the molecular mechanism by immunohistochemistry and tau protein phospho-proteomics. Neural. Regen. Res. 11:1579. 10.4103/1673-5374.193234 27904486 PMC5116834

[B321] Michopoulos V. Norrholm S. D. Jovanovic T. (2015). Diagnostic biomarkers for posttraumatic stress disorder: promising horizons from translational neuroscience research. Biol. Psychiatry 78, 344–353. 10.1016/j.biopsych.2015.01.005 25727177 PMC4520791

[B322] Minter M. R. Hinterleitner R. Meisel M. Zhang C. Leone V. Zhang X.. (2017). Antibiotic-induced perturbations in microbial diversity during post-natal development alters amyloid pathology in an aged APPSWE/PS1ΔE9 murine model of Alzheimer's disease. Sci. Rep. 7:10411. 10.1038/s41598-017-11047-w 28874832 PMC5585265

[B323] Minter M. R. Zhang C. Leone V. Ringus D. L. Zhang X. Oyler-Castrillo P.. (2016). Antibiotic-induced perturbations in gut microbial diversity influences neuro-inflammation and amyloidosis in a murine model of Alzheimer's disease. Sci. Rep. 6:30028. 10.1038/srep30028 27443609 PMC4956742

[B324] Minuk G. Y. Lewkonia R. M. (1986). Possible familial association of multiple sclerosis and inflammatory bowel disease. N. Engl. J. Med. 314, 586–586. 10.1056/NEJM198602273140921 3945303

[B325] Miranda O. Qi X. Brannock M. D. Whitworth R. Kosten T. Ryan N. D.. (2025). Emulating a randomized clinical trial with real-world data to evaluate the effect of antidepressant use in PTSD patients with high suicide risk. Front. Psychiatry 15:1526488. 10.3389/fpsyt.2024.1526488 39935628 PMC11811752

[B326] Mirashrafi S. Hejazi Taghanaki S. Z. Sarlak F. Moravejolahkami A. R. Hojjati Kermani M. A. Haratian M. (2021). Effect of probiotics supplementation on disease progression, depression, general health, and anthropometric measurements in relapsing-remitting multiple sclerosis patients: a systematic review and meta-analysis of clinical trials. Int. J. Clin. Pract. 75:e14724. 10.1111/ijcp.14724 34379879

[B327] Mirzaei R. Bouzari B. Hosseini-Fard S. R. Mazaheri M. Ahmadyousefi Y. Abdi M.. (2021). Role of microbiota-derived short-chain fatty acids in nervous system disorders. Biomed. Pharmacother. 139:111661. 10.1016/j.biopha.2021.111661 34243604

[B328] Miyake S. Kim S. Suda W. Oshima K. Nakamura M. Matsuoka T.. (2015). Dysbiosis in the gut microbiota of patients with multiple sclerosis, with a striking depletion of species belonging to Clostridia XIVa and IV clusters. PLoS ONE 10:e0137429. 10.1371/journal.pone.0137429 26367776 PMC4569432

[B329] Miyaoka T. Kanayama M. Wake R. Hashioka S. Hayashida M. Nagahama M.. (2018). *Clostridium butyricum* MIYAIRI 588 as adjunctive therapy for treatment-resistant major depressive disorder: a prospective open-label trial. Clin. Neuropharm. 41, 151–155. 10.1097/WNF.0000000000000299 30234616

[B330] Monteiro-Cardoso V. F. Corlianò M. Singaraja R. R. (2021). Bile acids: a communication channel in the gut-brain axis. Neuromol. Med. 23, 99–117. 10.1007/s12017-020-08625-z 33085065

[B331] Mousten I. V. Sørensen N. V. Christensen R. H. B. Benros M. E. (2022). Cerebrospinal fluid biomarkers in patients with unipolar depression compared with healthy control individuals: a systematic review and meta-analysis. JAMA Psychiatry 79:571. 10.1001/jamapsychiatry.2022.0645 35442429 PMC9021989

[B332] Mulak A. (2021). Bile acids as key modulators of the brain-gut-microbiota axis in Alzheimer's disease. JAD 84, 461–477. 10.3233/JAD-210608 34569953 PMC8673511

[B333] Musiek E. S. Xiong D. D. Holtzman D. M. (2015). Sleep, circadian rhythms, and the pathogenesis of Alzheimer disease. Exp. Mol. Med. 47:e148. 10.1038/emm.2014.121 25766617 PMC4351409

[B334] Mydeen A. B. Nakhal M. M. Almazrouei R. Alkamali R. Alsulaimi M. Aleissaee O.. (2025). Selective vulnerability of stellate cells to gut dysbiosis: neuroanatomical changes in the medial entorhinal cortex. Front. Neuroanat. 19:1589287. 10.3389/fnana.2025.1589287 40880682 PMC12380798

[B335] Nabizadeh F. Valizadeh P. Fallahi M. S. Alzheimer's Disease Neuroimaging Initiative (2024). Bile acid profile associated with CSF and PET biomarkers in Alzheimer's disease. Aging Clin. Exp. Res. 36:62. 10.1007/s40520-024-02729-3 38451317 PMC10920417

[B336] Nagpal R. Shively C. A. Register T. C. Craft S. Yadav H. (2019). Gut microbiome-Mediterranean diet interactions in improving host health. F1000Research 8:699. 10.12688/f1000research.18992.1 32704349 PMC7359750

[B337] Nakhal M. M. Mydeen A. B. Yassin L. K. Almazrouei R. Alkamali R. Alsulaimi M.. (2025a). Antibiotics-induced dysbiosis impacts dendritic morphology of adult mouse cortical interneurons. Front. Neuroanat. 19:1557961. 10.3389/fnana.2025.1557961 40124111 PMC11925899

[B338] Nakhal M. M. Yassin L. K. Al Houqani S. Mydeen A. B. Ibrahim M. F. Shehab S.. (2025b). Early-life stress caused by maternal deprivation impacts dendritic morphology of adult male mouse neocortical interneurons. Int. J. Mol. Sci. 26:1909. 10.3390/ijms26051909 40076536 PMC11900613

[B339] Nakhal M. M. Yassin L. K. Alyaqoubi R. Saeed S. Alderei A. Alhammadi A.. (2024). The microbiota-gut-brain axis and neurological disorders: a comprehensive review. Life 14:1234. 10.3390/life14101234 39459534 PMC11508655

[B340] Naseribafrouei A. Hestad K. Avershina E. Sekelja M. Linløkken A. Wilson R.. (2014). Correlation between the human fecal microbiota and depression. Neurogastroenterol. Motil. 26, 1155–1162. 10.1111/nmo.12378 24888394

[B341] Neufeld K. M. Kang N. Bienenstock J. Foster J. A. (2011). Reduced anxiety-like behavior and central neurochemical change in germ-free mice. Neurogastroenterol. Motil. 23, 255–264, e119. 10.1111/j.1365-2982.2010.01620.x 21054680

[B342] Niccolai E. Martinelli I. Quaranta G. Nannini G. Zucchi E. De Maio F.. (2024). Fecal microbiota transplantation in amyotrophic lateral sclerosis: clinical protocol and evaluation of microbiota immunity axis. Methods Mol. Biol. 2761, 373–396. 10.1007/978-1-0716-3662-6_27 38427251

[B343] Nichol K. E. Poon W. W. Parachikova A. I. Cribbs D. H. Glabe C. G. Cotman C. W. (2008). Exercise alters the immune profile in Tg2576 Alzheimer mice toward a response coincident with improved cognitive performance and decreased amyloid. J. Neuroinflamm. 5:13. 10.1186/1742-2094-5-13 18400101 PMC2329612

[B344] Nierenberg A. A. Agustini B. Köhler-Forsberg O. Cusin C. Katz D. Sylvia L. G.. (2023). Diagnosis and treatment of bipolar disorder: a review. JAMA 330, 1370–1380. 10.1001/jama.2023.18588 37815563

[B345] Nihart A. J. Garcia M. A. El Hayek E. Liu R. Olewine M. Kingston J. D.. (2025). Bioaccumulation of microplastics in decedent human brains. Nat. Med. 31, 1114–1119. 10.1038/s41591-024-03453-1 39901044 PMC12003191

[B346] Nusrat S. Gulick E. Levinthal D. Bielefeldt K. (2012). Anorectal dysfunction in multiple sclerosis: a systematic review. ISRN Neurol. 2012, 1–9. 10.5402/2012/376023 22900202 PMC3414061

[B347] Obeso J. A. Stamelou M. Goetz C. G. Poewe W. Lang A. E. Weintraub D.. (2017). Past, present, and future of Parkinson's disease: a special essay on the 200th Anniversary of the Shaking Palsy. Mov. Disord. 32, 1264–1310. 10.1002/mds.27115 28887905 PMC5685546

[B348] Obrenovich M. Jaworski H. Tadimalla T. Mistry A. Sykes L. Perry G.. (2020). The role of the microbiota-gut-brain axis and antibiotics in ALS and neurodegenerative diseases. Microorganisms 8:784. 10.3390/microorganisms8050784 32456229 PMC7285349

[B349] Ochoa-Repáraz J. Mielcarz D. W. Ditrio L. E. Burroughs A. R. Foureau D. M. Haque-Begum S.. (2009). Role of gut commensal microflora in the development of experimental autoimmune encephalomyelitis. J. Immunol. 183, 6041–6050. 10.4049/jimmunol.0900747 19841183

[B350] O'Hare M. A. Swart P. C. Malan-Müller S. Van Den Heuvel L. L. Bröcker E. Seedat S.. (2024). The saNeuroGut initiative: investigating the gut microbiome and symptoms of anxiety, depression, and posttraumatic stress. Neuroimmunomodulation 32, 1–15. 10.1159/000542696 39561720 PMC11844704

[B351] Olanow C. W. Kieburtz K. Odin P. Espay A. J. Standaert D. G. Fernandez H. H.. (2014). Continuous intrajejunal infusion of levodopa-carbidopa intestinal gel for patients with advanced Parkinson's disease: a randomised, controlled, double-blind, double-dummy study. Lancet Neurol. 13, 141–149. 10.1016/S1474-4422(13)70293-X 24361112 PMC4643396

[B352] O'Mahony S. M. Felice V. D. Nally K. Savignac H. M. Claesson M. J. Scully P.. (2014). Disturbance of the gut microbiota in early-life selectively affects visceral pain in adulthood without impacting cognitive or anxiety-related behaviors in male rats. Neuroscience 277, 885–901. 10.1016/j.neuroscience.2014.07.054 25088912

[B353] Özsoy S. Gündogdu S. Sezigen S. Tasalp E. Ikiz D. A. Kideys A. E. (2024). Presence of microplastics in human stomachs. Forensic Sci. Int. 364:112246. 10.1016/j.forsciint.2024.112246 39413612

[B354] Painold A. Mörkl S. Kashofer K. Halwachs B. Dalkner N. Bengesser S.. (2019). A step ahead: exploring the gut microbiota in inpatients with bipolar disorder during a depressive episode. Bipolar Disord. 21, 40–49. 10.1111/bdi.12682 30051546 PMC6585963

[B355] Palacios N. Hannoun A. Flahive J. Ward D. Goostrey K. Deb A.. (2021). Effect of levodopa initiation on the gut microbiota in Parkinson's disease. Front. Neurol. 12:574529. 10.3389/fneur.2021.574529 33746867 PMC7970035

[B356] Paley E. L. Merkulova-Rainon T. Faynboym A. Shestopalov V. I. Aksenoff I. (2018). Geographical distribution and diversity of gut microbial NADH: ubiquinone oxidoreductase sequence associated with Alzheimer's disease. J. Alzheimer. Dis. 61, 1531–1540. 10.3233/JAD-170764 29376868

[B357] Palm N. W. De Zoete M. R. Flavell R. A. (2015). Immune–microbiota interactions in health and disease. Clin. Immunol. 159, 122–127. 10.1016/j.clim.2015.05.014 26141651 PMC4943041

[B358] Parizadeh M. Arrieta M.-C. (2023). The global human gut microbiome: genes, lifestyles, and diet. Trends Mol. Med. 29, 789–801. 10.1016/j.molmed.2023.07.002 37516570

[B359] Park S.-H. Lee J. H. Shin J. Kim J.-S. Cha B. Lee S.. (2021). Cognitive function improvement after fecal microbiota transplantation in Alzheimer's dementia patient: a case report. Curr. Med. Res. Opin. 37, 1739–1744. 10.1080/03007995.2021.1957807 34289768

[B360] Parobkova E. Matej R. (2021). Amyotrophic lateral sclerosis and frontotemporal lobar degenerations: similarities in genetic background. Diagnostics 11:509. 10.3390/diagnostics11030509 33805659 PMC7998502

[B361] Parrella E. Gussago C. Porrini V. Benarese M. Pizzi M. (2020). From preclinical stroke models to humans: polyphenols in the prevention and treatment of stroke. Nutrients 13:85. 10.3390/nu13010085 33383852 PMC7823436

[B362] Pavan S. Prabhu A. N. Prasad Gorthi S. Das B. Mutreja A. Shetty V.. (2022). Exploring the multifactorial aspects of gut microbiome in Parkinson's disease. Folia Microbiol. 67, 693–706. 10.1007/s12223-022-00977-2 35583791 PMC9526693

[B363] Peh A. O'Donnell J. A. Broughton B. R. S. Marques F. Z. (2022). Gut microbiota and their metabolites in stroke: a double-edged sword. Stroke 53, 1788–1801. 10.1161/STROKEAHA.121.036800 35135325

[B364] Peng Y. Lu J. Fan L. Dong W. Jiang M. (2024). Simulated gastrointestinal digestion of two different sources of biodegradable microplastics and the influence on gut microbiota. Food Chem. Toxicol. 185, 114474. 10.1016/j.fct.2024.114474 38301992

[B365] Penninx B. W. Milaneschi Y. Lamers F. Vogelzangs N. (2013). Understanding the somatic consequences of depression: biological mechanisms and the role of depression symptom profile. BMC Med. 11:129. 10.1186/1741-7015-11-129 23672628 PMC3661358

[B366] Perrin M. Vandeleur C. L. Castelao E. Rothen S. Glaus J. Vollenweider P.. (2014). Determinants of the development of post-traumatic stress disorder, in the general population. Soc. Psychiatry Psychiatr. Epidemiol. 49, 447–457. 10.1007/s00127-013-0762-3 24022753

[B367] Petakh P. Oksenych V. Kamyshna I. Boisak I. Lyubomirskaya K. Kamyshnyi O. (2024). Exploring the interplay between posttraumatic stress disorder, gut microbiota, and inflammatory biomarkers: a comprehensive meta-analysis. Front. Immunol. 15:1349883. 10.3389/fimmu.2024.1349883 38410510 PMC10895958

[B368] Pfeiffer R. F. (2003). Gastrointestinal dysfunction in Parkinson's disease. Lancet Neurol. 2, 107–116. 10.1016/S1474-4422(03)00307-7 12849267

[B369] Pierantozzi M. Pietroiusti A. Sancesario G. Lunardi G. Fedele E. Giacomini P.. (2001). Reduced L -dopa absorption and increased clinical fluctuations in *Helicobacter pylori*-infected Parkinson's disease patients. Neurol. Sci. 22, 89–91. 10.1007/s100720170061 11487216

[B370] Poonacha K. N. T. Villa T. G. Notario V. (2022). The interplay among radiation therapy, antibiotics and the microbiota: impact on cancer treatment outcomes. Antibiotics 11:331. 10.3390/antibiotics11030331 35326794 PMC8944497

[B371] Prasad A. Bharathi V. Sivalingam V. Girdhar A. Patel B. K. (2019). Molecular mechanisms of TDP-43 misfolding and pathology in amyotrophic lateral sclerosis. Front. Mol. Neurosci. 12:25. 10.3389/fnmol.2019.00025 30837838 PMC6382748

[B372] Preiningerova J. L. Jiraskova Zakostelska Z. Srinivasan A. Ticha V. Kovarova I. Kleinova P.. (2022). Multiple sclerosis and microbiome. Biomolecules 12:433. 10.3390/biom12030433 35327624 PMC8946130

[B373] Puigbò P. Leino L. I. Rainio M. J. Saikkonen K. Saloniemi I. Helander M. (2022). Does glyphosate affect the human microbiota? Life 12:707. 10.3390/life12050707 35629374 PMC9145961

[B374] Qian Q. Pu Q. Li L. Wu J. Cheng G. Cheng Y.. (2025). Polylactic acid microplastics before and after aging induced neurotoxicity in zebrafish by disrupting the microbiota-gut-brain axis. J. Hazard. Mater. 488:137306. 10.1016/j.jhazmat.2025.137306 39864199

[B375] Qian Y. Yang X. Xu S. Wu C. Song Y. Qin N.. (2018). Alteration of the fecal microbiota in Chinese patients with Parkinson's disease. Brain Behav. Immun. 70, 194–202. 10.1016/j.bbi.2018.02.016 29501802

[B376] Qu L. Liu F. Fang Y. Wang L. Chen H. Yang Q.. (2023). Improvement in zebrafish with diabetes and Alzheimer's disease treated with pasteurized akkermansia muciniphila. Microbiol. Spectr. 11, e00849–e00823. 10.1128/spectrum.00849-23 37191572 PMC10269592

[B377] Ranisavljev M. Stajer V. Todorovic N. Ostojic J. Cvejic J. H. Steinert R. E.. (2024). The effects of 3-month supplementation with synbiotic on patient-reported outcomes, exercise tolerance, and brain and muscle metabolism in adult patients with post-COVID-19 chronic fatigue syndrome (STOP-FATIGUE): a randomized Placebo-controlled clinical trial. Eur. J. Nutr. 64:28. 10.1007/s00394-024-03546-0 39592468

[B378] Ravi M. Miller A. H. Michopoulos V. (2021). The immunology of stress and the impact of inflammation on the brain and behaviour. BJPsych Adv. 27, 158–165. 10.1192/bja.2020.82 34055387 PMC8158089

[B379] Renton A. E. Majounie E. Waite A. Simón-Sánchez J. Rollinson S. Gibbs J. R.. (2011). A hexanucleotide repeat expansion in C9ORF72 is the cause of chromosome 9p21-linked ALS-FTD. Neuron 72, 257–268. 10.1016/j.neuron.2011.09.010 21944779 PMC3200438

[B380] Rhee S. H. Pothoulakis C. Mayer E. A. (2009). Principles and clinical implications of the brain-gut-enteric microbiota axis. Nat. Rev. Gastroenterol. Hepatol. 6, 306–314. 10.1038/nrgastro.2009.35 19404271 PMC3817714

[B381] Roda A. Serra-Mir G. Montoliu-Gaya L. Tiessler L. Villegas S. (2022). Amyloid-beta peptide and tau protein crosstalk in Alzheimer's disease. Neural. Regen. Res. 17:1666. 10.4103/1673-5374.332127 35017413 PMC8820696

[B382] Rogers G. B. Keating D. J. Young R. L. Wong M.-L. Licinio J. Wesselingh S. (2016). From gut dysbiosis to altered brain function and mental illness: mechanisms and pathways. Mol. Psychiatry 21, 738–748. 10.1038/mp.2016.50 27090305 PMC4879184

[B383] Roh J. H. Huang Y. Bero A. W. Kasten T. Stewart F. R. Bateman R. J.. (2012). Disruption of the sleep-wake cycle and diurnal fluctuation of β-amyloid in mice with Alzheimer's disease pathology. Sci. Transl. Med. 4:122. 10.1126/scitranslmed.3004291 22956200 PMC3654377

[B384] Ruciński J. Kurowska-Rucińska E. Myślińska D. Grembecka B. Piekarczyk N. Necel A.. (2025). Galactooligosaccharides attenuate behavioural, haematological and immunological abnormalities and influence gut microbiota in rats with amygdala hyperactivation induced by electrical stimulation. IJMS 26:4353. 10.3390/ijms26094353 40362590 PMC12073049

[B385] Salminen S. Collado M. C. Endo A. Hill C. Lebeer S. Quigley E. M. M.. (2021). The international scientific association of probiotics and prebiotics (ISAPP) consensus statement on the definition and scope of postbiotics. Nat. Rev. Gastroenterol. Hepatol. 18, 649–667. 10.1038/s41575-021-00440-6 33948025 PMC8387231

[B386] Sampson T. R. (2023). Revealing gut microbiome associations with CFS. Cell Host Microbe 31, 171–172. 10.1016/j.chom.2023.01.011 36758517

[B387] Sampson T. R. Debelius J. W. Thron T. Janssen S. Shastri G. G. Ilhan Z. E.. (2016). Gut microbiota regulate motor deficits and neuroinflammation in a model of Parkinson's disease. Cell 167, 1469–1480.e12. 10.1016/j.cell.2016.11.018 27912057 PMC5718049

[B388] Sanada K. Nakajima S. Kurokawa S. Barceló-Soler A. Ikuse D. Hirata A.. (2020). Gut microbiota and major depressive disorder: a systematic review and meta-analysis. J. Affect. Disord. 266, 1–13. 10.1016/j.jad.2020.01.102 32056863

[B389] Sasaki D. Sasaki K. Ikuta N. Yasuda T. Fukuda I. Kondo A.. (2018). Low amounts of dietary fibre increase *in vitro* production of short-chain fatty acids without changing human colonic microbiota structure. Sci. Rep. 8:435. 10.1038/s41598-017-18877-8 29323180 PMC5765155

[B390] Savignac H. M. Kiely B. Dinan T. G. Cryan J. F. (2014). Bifidobacteria exert strain-specific effects on stress-related behavior and physiology in BALB/c mice. Neurogastroenterol. Motil. 26, 1615–1627. 10.1111/nmo.12427 25251188

[B391] Scheperjans F. Aho V. Pereira P. A. B. Koskinen K. Paulin L. Pekkonen E.. (2015). Gut microbiota are related to Parkinson's disease and clinical phenotype. Mov. Disord. 30, 350–358. 10.1002/mds.26069 25476529

[B392] Schwarz E. Maukonen J. Hyytiäinen T. Kieseppä T. Orešič M. Sabunciyan S.. (2018). Analysis of microbiota in first episode psychosis identifies preliminary associations with symptom severity and treatment response. Schizophr. Res. 192, 398–403. 10.1016/j.schres.2017.04.017 28442250

[B393] Seton K. A. Defernez M. Telatin A. Tiwari S. K. Savva G. M. Hayhoe A.. (2023). Investigating antibody reactivity to the intestinal microbiome in severe myalgic encephalomyelitis/chronic fatigue syndrome (ME/CFS): a feasibility study. Int. J. Mol. Sci. 24:15316. 10.3390/ijms242015316 37895005 PMC10607161

[B394] Shamsipour S. Sharifi G. Taghian F. (2021). An 8-week administration of *Bifidobacterium bifidum* and *Lactobacillus plantarum* combined with exercise training alleviates neurotoxicity of Aβ and spatial learning via acetylcholine in Alzheimer rat model. J. Mol. Neurosci. 71, 1495–1505. 10.1007/s12031-021-01812-y 33715084

[B395] Shehata A. A. Schrödl W. Aldin A. A. Hafez H. M. Krüger M. (2013). The effect of glyphosate on potential pathogens and beneficial members of poultry microbiota *in vitro*. Curr. Microbiol. 66, 350–358. 10.1007/s00284-012-0277-2 23224412

[B396] Shen L. Liu L. Ji H.-F. (2017). Alzheimer's disease histological and behavioral manifestations in transgenic mice correlate with specific gut microbiome state. J. Alzheimer. Dis. 56, 385–390. 10.3233/JAD-160884 27911317

[B397] Sherin J. E. Nemeroff C. B. (2011). Post-traumatic stress disorder: the neurobiological impact of psychological trauma. Dial. Clin. Neurosci. 13, 263–278. 10.31887/DCNS.2011.13.2/jsherin 22034143 PMC3182008

[B398] Shi H. Nelson J. W. Phillips S. Petrosino J. F. Bryan R. M. Durgan D. J. (2022). Alterations of the gut microbial community structure and function with aging in the spontaneously hypertensive stroke prone rat. Sci. Rep. 12:8534. 10.1038/s41598-022-12578-7 35595870 PMC9122926

[B399] Shin N.-R. Whon T. W. Bae J.-W. (2015). Proteobacteria: microbial signature of dysbiosis in gut microbiota. Trends Biotechnol. 33, 496–503. 10.1016/j.tibtech.2015.06.011 26210164

[B400] Silva Y. P. Bernardi A. Frozza R. L. (2020). The role of short-chain fatty acids from gut microbiota in gut-brain communication. Front. Endocrinol. 11:25. 10.3389/fendo.2020.00025 32082260 PMC7005631

[B401] Singh V. Roth S. Llovera G. Sadler R. Garzetti D. Stecher B.. (2016). Microbiota dysbiosis controls the neuroinflammatory response after stroke. J. Neurosci. 36, 7428–7440. 10.1523/JNEUROSCI.1114-16.2016 27413153 PMC6705544

[B402] Skjevling L. Goll R. Hanssen H. M. Johnsen P. H. (2024). Faecal microbiota transplantation (FMT) in Norwegian outpatients with mild to severe myalgic encephalomyelitis/chronic fatigue syndrome (ME/CFS): protocol for a 12-month randomised double-blind placebo-controlled trial. BMJ Open 14:e073275. 10.1136/bmjopen-2023-073275 38858151 PMC11168185

[B403] Slykerman R. F. Hood F. Wickens K. Thompson J. M. D. Barthow C. Murphy R.. (2017). Effect of *Lactobacillus rhamnosus* HN001 in pregnancy on postpartum symptoms of depression and anxiety: a randomised double-blind placebo-controlled trial. EBioMedicine 24, 159–165. 10.1016/j.ebiom.2017.09.013 28943228 PMC5652021

[B404] Socała K. Doboszewska U. Szopa A. Serefko A. Włodarczyk M. Zielińska A.. (2021). The role of microbiota-gut-brain axis in neuropsychiatric and neurological disorders. Pharmacol. Res. 172:105840. 10.1016/j.phrs.2021.105840 34450312

[B405] Solati J. Hajikhani R. Golub Y. (2013). Activation of GABA_A_ receptors in the medial prefrontal cortex produces an anxiolytic-like response. Acta Neuropsychiatr. 25, 221–226. 10.1111/acn.12016 25287635

[B406] Sorboni S. G. Moghaddam H. S. Jafarzadeh-Esfehani R. Soleimanpour S. (2022). A comprehensive review on the role of the gut microbiome in human neurological disorders. Clin. Microbiol. Rev. 35:e0033820. 10.1128/CMR.00338-20 34985325 PMC8729913

[B407] Souza P. B. D. De Araujo Borba L. Castro De Jesus L. Valverde A. P. Gil-Mohapel J. Rodrigues A. L. S. (2023). Major depressive disorder and gut microbiota: role of physical exercise. IJMS 24:16870. 10.3390/ijms242316870 38069198 PMC10706777

[B408] Spira A. P. Gamaldo A. A. An Y. Wu M. N. Simonsick E. M. Bilgel M.. (2013). Self-reported sleep and β-amyloid deposition in community-dwelling older adults. JAMA Neurol. 70, 1537–1543. 10.1001/jamaneurol.2013.4258 24145859 PMC3918480

[B409] Stallmach A. Quickert S. Puta C. Reuken P. A. (2024). The gastrointestinal microbiota in the development of ME/CFS: a critical view and potential perspectives. Front. Immunol. 15:1352744. 10.3389/fimmu.2024.1352744 38605969 PMC11007072

[B410] Stanley D. Mason L. J. Mackin K. E. Srikhanta Y. N. Lyras D. Prakash M. D.. (2016). Translocation and dissemination of commensal bacteria in post-stroke infection. Nat. Med. 22, 1277–1284. 10.1038/nm.4194 27694934

[B411] Stein M. B. Chen C.-Y. Ursano R. J. Cai T. Gelernter J. Heeringa S. G.. (2016). Genome-wide association studies of posttraumatic stress disorder in 2 cohorts of US army soldiers. JAMA Psychiatry 73:695. 10.1001/jamapsychiatry.2016.0350 27167565 PMC4936936

[B412] Stilling R. M. Van De Wouw M. Clarke G. Stanton C. Dinan T. G. Cryan J. F. (2016). The neuropharmacology of butyrate: the bread and butter of the microbiota-gut-brain axis? Neurochem. Int. 99, 110–132. 10.1016/j.neuint.2016.06.011 27346602

[B413] Stolfi C. Maresca C. Monteleone G. Laudisi F. (2022). Implication of intestinal barrier dysfunction in gut dysbiosis and diseases. Biomedicines 10:289. 10.3390/biomedicines10020289 35203499 PMC8869546

[B414] Strandwitz P. Kim K. H. Terekhova D. Liu J. K. Sharma A. Levering J.. (2018). GABA-modulating bacteria of the human gut microbiota. Nat. Microbiol. 4, 396–403. 10.1038/s41564-018-0307-3 30531975 PMC6384127

[B415] Sudo N. Chida Y. Aiba Y. Sonoda J. Oyama N. Yu X.-N.. (2004). Postnatal microbial colonization programs the hypothalamic-pituitary-adrenal system for stress response in mice. J. Physiol. 558, 263–275. 10.1113/jphysiol.2004.063388 15133062 PMC1664925

[B416] Sun J. Li H. Jin Y. Yu J. Mao S. Su K.-P.. (2021). Probiotic *Clostridium butyricum* ameliorated motor deficits in a mouse model of Parkinson's disease via gut microbiota-GLP-1 pathway. Brain Behav. Immun. 91, 703–715. 10.1016/j.bbi.2020.10.014 33148438

[B417] Sun J. Liu S. Ling Z. Wang F. Ling Y. Gong T.. (2019a). Fructooligosaccharides ameliorating cognitive deficits and neurodegeneration in APP/PS1 transgenic mice through modulating gut microbiota. J. Agric. Food Chem. 67, 3006–3017. 10.1021/acs.jafc.8b07313 30816709

[B418] Sun J. Peng S. Yang Q. Yang J. Dai Y. Xing L. (2025). Microplastics/nanoplastics and neurological health: an overview of neurological defects and mechanisms. Toxicology 511:154030. 10.1016/j.tox.2024.154030 39653181

[B419] Sun J. Xu J. Ling Y. Wang F. Gong T. Yang C.. (2019b). Fecal microbiota transplantation alleviated Alzheimer's disease-like pathogenesis in APP/PS1 transgenic mice. Transl. Psychiatry 9:189. 10.1038/s41398-019-0525-3 31383855 PMC6683152

[B420] Sutherland V. L. McQueen C. A. Mendrick D. Gulezian D. Cerniglia C. Foley S.. (2020). The gut microbiome and xenobiotics: identifying knowledge gaps. Toxicol. Sci. 176, 1–10. 10.1093/toxsci/kfaa060 32658296 PMC7850111

[B421] Svensson E. Horváth-Puhó E. Thomsen R. W. Djurhuus J. C. Pedersen L. Borghammer P.. (2015). Vagotomy and subsequent risk of P arkinson's disease. Ann. Neurol. 78, 522–529. 10.1002/ana.24448 26031848

[B422] Tamtaji O. R. Taghizadeh M. Daneshvar Kakhaki R. Kouchaki E. Bahmani F. Borzabadi S.. (2019). Clinical and metabolic response to probiotic administration in people with Parkinson's disease: a randomized, double-blind, placebo-controlled trial. Clin. Nutr. 38, 1031–1035. 10.1016/j.clnu.2018.05.018 29891223

[B423] Tan A. H. Lim S.-Y. Chong K. K. A Manap M. A. A. Hor J. W. Lim J. L.. (2021). Probiotics for constipation in Parkinson disease: a randomized placebo-controlled study. Neurology 96, e772–e782. 10.1212/WNL.0000000000010998 33046607

[B424] Tanca A. Abbondio M. Palomba A. Fraumene C. Manghina V. Cucca F.. (2017). Potential and active functions in the gut microbiota of a healthy human cohort. Microbiome 5:79. 10.1186/s40168-017-0293-3 28709472 PMC5513205

[B425] Tanelian A. Nankova B. Cheriyan A. Arens C. Hu F. Sabban E. L. (2023). Differences in gut microbiota associated with stress resilience and susceptibility to single prolonged stress in female rodents. Neurobiol. Stress 24:100533. 10.1016/j.ynstr.2023.100533 36970450 PMC10034505

[B426] Tankou S. K. Regev K. Healy B. C. Cox L. M. Tjon E. Kivisakk P.. (2018a). Investigation of probiotics in multiple sclerosis. Mult. Scler. 24, 58–63. 10.1177/1352458517737390 29307299

[B427] Tankou S. K. Regev K. Healy B. C. Tjon E. Laghi L. Cox L. M.. (2018b). A probiotic modulates the microbiome and immunity in multiple sclerosis. Ann. Neurol. 83, 1147–1161. 10.1002/ana.25244 29679417 PMC6181139

[B428] Taylor J. P. Brown R. H. Cleveland D. W. (2016). Decoding ALS: from genes to mechanism. Nature 539, 197–206. 10.1038/nature20413 27830784 PMC5585017

[B429] Tejkalová H. Jakob L. Kvasnová S. Klaschka J. Sechovcová H. Mrázek J.. (2023). The influence of antibiotic treatment on the behavior and gut microbiome of adult rats neonatally insulted with lipopolysaccharide. Heliyon 9:e15417. 10.1016/j.heliyon.2023.e15417 37123951 PMC10130227

[B430] Thabet E. Dief A. E. Arafa S. A.-F. Yakout D. Ali M. A. (2024). Antibiotic-induced gut microbe dysbiosis alters neurobehavior in mice through modulation of BDNF and gut integrity. Physiol. Behav. 283:114621. 10.1016/j.physbeh.2024.114621 38925433

[B431] Thakur S. Dhapola R. Sarma P. Medhi B. Reddy D. H. (2023). Neuroinflammation in Alzheimer's disease: current progress in molecular signaling and therapeutics. Inflammation 46, 1–17. 10.1007/s10753-022-01721-1 35986874

[B432] Thal D. R. Del Tredici K. Ludolph A. C. Hoozemans J. J. M. Rozemuller A. J. Braak H.. (2011). Stages of granulovacuolar degeneration: their relation to Alzheimer's disease and chronic stress response. Acta Neuropathol. 122, 577–589. 10.1007/s00401-011-0871-6 21935637

[B433] Thompson A. J. Banwell B. L. Barkhof F. Carroll W. M. Coetzee T. Comi G.. (2018). Diagnosis of multiple sclerosis: 2017 revisions of the McDonald criteria. Lancet Neurol. 17, 162–173. 10.1016/S1474-4422(17)30470-2 29275977

[B434] Thompson R. S. Vargas F. Dorrestein P. C. Chichlowski M. Berg B. M. Fleshner M. (2020). Dietary prebiotics alter novel microbial dependent fecal metabolites that improve sleep. Sci. Rep. 10:3848. 10.1038/s41598-020-60679-y 32123201 PMC7051969

[B435] Tian P. Wang G. Zhao J. Zhang H. Chen W. (2019a). Bifidobacterium with the role of 5-hydroxytryptophan synthesis regulation alleviates the symptom of depression and related microbiota dysbiosis. J. Nutr. Biochem. 66, 43–51. 10.1016/j.jnutbio.2019.01.007 30743155

[B436] Tian P. Zou R. Song L. Zhang X. Jiang B. Wang G.. (2019b). Ingestion of *Bifidobacterium longum* subspecies *infantis* strain CCFM687 regulated emotional behavior and the central BDNF pathway in chronic stress-induced depressive mice through reshaping the gut microbiota. Food Funct. 10, 7588–7598. 10.1039/C9FO01630A 31687714

[B437] Torrisi S. A. Leggio G. M. Drago F. Salomone S. (2019). Therapeutic challenges of post-traumatic stress disorder: focus on the dopaminergic system. Front. Pharmacol. 10:404. 10.3389/fphar.2019.00404 31057408 PMC6478703

[B438] Touchefeu Y. Montassier E. Nieman K. Gastinne T. Potel G. Bruley des Varannes S.. (2014). Systematic review: the role of the gut microbiota in chemotherapy- or radiation-induced gastrointestinal mucositis – current evidence and potential clinical applications. Alimentary Pharmacol. Therap. 40, 409–421. 10.1111/apt.12878 25040088

[B439] Tremlett H. Fadrosh D. W. Faruqi A. A. Hart J. Roalstad S. Graves J.. (2016). Gut microbiota composition and relapse risk in pediatric MS: a pilot study. J. Neurol. Sci. 363, 153–157. 10.1016/j.jns.2016.02.042 27000242 PMC4806409

[B440] Troubat R. Barone P. Leman S. Desmidt T. Cressant A. Atanasova B.. (2021). Neuroinflammation and depression: a review. Eur. J. Neurosci. 53, 151–171. 10.1111/ejn.14720 32150310

[B441] Tuttolomondo A. Simonetta I. Daidone M. Mogavero A. Ortello A. Pinto A. (2019). Metabolic and vascular effect of the mediterranean diet. Int. J. Mol. Sci. 20:4716. 10.3390/ijms20194716 31547615 PMC6801699

[B442] Tyagi A. Choi Y.-Y. Shan L. Vinothkanna A. Lee E.-S. Chelliah R.. (2025). Limosilactobacillus reuteri fermented brown rice alleviates anxiety improves cognition and modulates gut microbiota in stressed mice. NPJ Sci. Food 9:5. 10.1038/s41538-025-00369-z 39799113 PMC11724862

[B443] Uemura N. Yagi H. Uemura M. T. Hatanaka Y. Yamakado H. Takahashi R. (2018). Inoculation of α-synuclein preformed fibrils into the mouse gastrointestinal tract induces Lewy body-like aggregates in the brainstem via the vagus nerve. Mol. Neurodegener. 13:21. 10.1186/s13024-018-0257-5 29751824 PMC5948849

[B444] Ugidos N. Mena J. Baquero S. Alloza I. Azkargorta M. Elortza F.. (2019). Interactome of the autoimmune risk protein ANKRD55. Front. Immunol. 10:2067. 10.3389/fimmu.2019.02067 31620119 PMC6759997

[B445] Uhde M. Indart A. C. Green P. H. R. Yolken R. H. Cook D. B. Shukla S. K.. (2023). Suppressed immune and metabolic responses to intestinal damage-associated microbial translocation in myalgic encephalomyelitis/chronic fatigue syndrome. Brain Behav. Immun. Health 30:100627. 10.1016/j.bbih.2023.100627 37396339 PMC10308215

[B446] Unger M. M. Spiegel J. Dillmann K.-U. Grundmann D. Philippeit H. Bürmann J.. (2016). Short chain fatty acids and gut microbiota differ between patients with Parkinson's disease and age-matched controls. Parkinson. Relat. Disord. 32, 66–72. 10.1016/j.parkreldis.2016.08.019 27591074

[B447] Vagnerová K. Vodička M. Hermanová P. Ergang P. Šrutková D. Klusonová P.. (2019). Interactions between gut microbiota and acute restraint stress in peripheral structures of the hypothalamic–pituitary–adrenal axis and the intestine of male mice. Front. Immunol. 10:2655. 10.3389/fimmu.2019.02655 31798585 PMC6878942

[B448] Valeri F. Dos Santos Guilherme M. He F. Stoye N. M. Schwiertz A. Endres K. (2021). Impact of the age of cecal material transfer donors on Alzheimer's disease pathology in 5xFAD mice. Microorganisms 9:2548. 10.3390/microorganisms9122548 34946148 PMC8708188

[B449] Valizadeh S. Majdi Seghinsara A. Maleki Chollou K. Bahadori A. Abbaszadeh S. Taghdir M.. (2021). The efficacy of probiotics in experimental autoimmune encephalomyelitis (an animal model for MS): a systematic review and meta-analysis. Lett. Appl. Microbiol. 73, 408–417. 10.1111/lam.13543 34310737

[B450] Valles-Colomer M. Falony G. Darzi Y. Tigchelaar E. F. Wang J. Tito R. Y.. (2019). The neuroactive potential of the human gut microbiota in quality of life and depression. Nat. Microbiol. 4, 623–632. 10.1038/s41564-018-0337-x 30718848

[B451] van den Bos M. A. J. Geevasinga N. Higashihara M. Menon P. Vucic S. (2019). Pathophysiology and diagnosis of ALS: insights from advances in neurophysiological techniques. Int. J. Mol. Sci. 20:2818. 10.3390/ijms20112818 31185581 PMC6600525

[B452] Varesi A. Deumer U.-S. Ananth S. Ricevuti G. (2021). The emerging role of gut microbiota in myalgic encephalomyelitis/chronic fatigue syndrome (ME/CFS): current evidence and potential therapeutic applications. J. Clin. Med 10:5077. 10.3390/jcm10215077 34768601 PMC8584653

[B453] Vendrik K. E. W. Ooijevaar R. E. De Jong P. R. C. Laman J. D. Van Oosten B. W. Van Hilten J. J.. (2020). Fecal microbiota transplantation in neurological disorders. Front. Cell. Infect. Microbiol. 10:98. 10.3389/fcimb.2020.00098 32266160 PMC7105733

[B454] Venkidesh B. S. Narasimhamurthy R. K. Jnana A. Reghunathan D. Sharan K. Chandraguthi S. G.. (2023). Pelvic irradiation induces behavioural and neuronal damage through gut dysbiosis in a rat model. Chem. Biol. Interact. 386:110775. 10.1016/j.cbi.2023.110775 37866488

[B455] Vettorazzi J. F. Kurauti M. A. Soares G. M. Borck P. C. Ferreira S. M. Branco R. C. S.. (2017). Bile acid TUDCA improves insulin clearance by increasing the expression of insulin-degrading enzyme in the liver of obese mice. Sci. Rep. 7:14876. 10.1038/s41598-017-13974-0 29093479 PMC5665899

[B456] Vogl T. Kalka I. N. Klompus S. Leviatan S. Weinberger A. Segal E. (2022). Systemic antibody responses against human microbiota flagellins are overrepresented in chronic fatigue syndrome patients. Sci. Adv. 8:eabq2422. 10.1126/sciadv.abq2422 36149952 PMC11580831

[B457] Vogt N. M. Kerby R. L. Dill-McFarland K. A. Harding S. J. Merluzzi A. P. Johnson S. C.. (2017). Gut microbiome alterations in Alzheimer's disease. Sci. Rep. 7:13537. 10.1038/s41598-017-13601-y 29051531 PMC5648830

[B458] Vogt N. M. Romano K. A. Darst B. F. Engelman C. D. Johnson S. C. Carlsson C. M.. (2018). The gut microbiota-derived metabolite trimethylamine N-oxide is elevated in Alzheimer's disease. Alzheimers. Res. Ther. 10:124. 10.1186/s13195-018-0451-2 30579367 PMC6303862

[B459] Voigt R. M. Engen P. A. Villanueva M. Bambi S. A. Green S. J. Naqib A.. (2025). Prebiotics as an adjunct therapy for posttraumatic stress disorder: a pilot randomized controlled trial. Front. Neurosci. 18:1477519. 10.3389/fnins.2024.1477519 39840022 PMC11747240

[B460] Wanapaisan P. Chuansangeam M. Nopnipa S. Mathuranyanon R. Nonthabenjawan N. Ngamsombat C.. (2022). Association between gut microbiota with mild cognitive impairment and Alzheimer's disease in a Thai population. Neurodegener. Dis. 22, 43–54. 10.1159/000526947 36070704

[B461] Wang A. Ling Z. Yang Z. Kiela P. R. Wang T. Wang C.. (2015). Gut microbial dysbiosis may predict diarrhea and fatigue in patients undergoing pelvic cancer radiotherapy: a pilot study. PLoS ONE 10:e0126312. 10.1371/journal.pone.0126312 25955845 PMC4425680

[B462] Wang J. Liu X. Li Q. (2023a). Interventional strategies for ischemic stroke based on the modulation of the gut microbiota. Front. Neurosci. 17:1158057. 10.3389/fnins.2023.1158057 36937662 PMC10017736

[B463] Wang J. Tian H. Shi Y. Yang Y. Yu F. Cao H.. (2023b). The enhancement in toxic potency of oxidized functionalized polyethylene-microplastics in mice gut and Caco-2 cells. Sci. Total Environ. 903:166057. 10.1016/j.scitotenv.2023.166057 37553056

[B464] Wang J. Yang Y. Shi Y. Wei L. Gao L. Liu M. (2024). Oxidized/unmodified-polyethylene microplastics neurotoxicity in mice: Perspective from microbiota-gut-brain axis. Environ. Int. 185:108523. 10.1016/j.envint.2024.108523 38484610

[B465] Wang J.-H. Choi Y. Lee J.-S. Hwang S.-J. Gu J. Son C.-G. (2024). Clinical evidence of the link between gut microbiome and myalgic encephalomyelitis/chronic fatigue syndrome: a retrospective review. Eur. J. Med. Res. 29:148. 10.1186/s40001-024-01747-1 38429822 PMC10908121

[B466] Wang L. Wang X. Zhang G. Ma Y. Zhang Q. Li Z.. (2021). The impact of pelvic radiotherapy on the gut microbiome and its role in radiation-induced diarrhoea: a systematic review. Radiat. Oncol. 16, 187. 10.1186/s13014-021-01899-y 34563216 PMC8466721

[B467] Wang M. Cao J. Gong C. Amakye W. K. Yao M. Ren J. (202b). Exploring the microbiota-Alzheimer's disease linkage using short-term antibiotic treatment followed by fecal microbiota transplantation. Brain Behav. Immun. 96, 227–238. 10.1016/j.bbi.2021.06.003 34111528

[B468] Wang M. Pan W. Xu Y. Zhang J. Wan J. Jiang H. (2022). Microglia-mediated neuroinflammation: a potential target for the treatment of cardiovascular diseases. J. Inflamm. Res. 15, 3083–3094. 10.2147/JIR.S350109 35642214 PMC9148574

[B469] Wang Q. Luo Y. Ray Chaudhuri K. Reynolds R. Tan E.-K. Pettersson S. (2021). The role of gut dysbiosis in Parkinson's disease: mechanistic insights and therapeutic options. Brain 144, 2571–2593. 10.1093/brain/awab156 33856024

[B470] Wang Q. Xu G. Yan O. Wang S. Wang X. (2025). Radiation-induced injury and the gut microbiota: insights from a microbial perspective. Therap. Adv. Gastroenterol. 18:17562848251347347. 10.1177/17562848251347347 40535532 PMC12174693

[B471] Wang Q.-J. Shen Y.-E. Wang X. Fu S. Zhang X. Zhang Y.-N.. (2020). Concomitant memantine and *Lactobacillus plantarum* treatment attenuates cognitive impairments in APP/PS1 mice. Aging 12, 628–649. 10.18632/aging.102645 31907339 PMC6977692

[B472] Wang T. Yuan F. Chen Z. Zhu S. Chang Z. Yang W.. (2020). Vascular, inflammatory and metabolic risk factors in relation to dementia in Parkinson's disease patients with type 2 diabetes mellitus. Aging 12, 15682–15704. 10.18632/aging.103776 32805719 PMC7467390

[B473] Wang Y. Wang D. Lv H. Dong Q. Li J. Geng W.. (2022). Modulation of the gut microbiota and glycometabolism by a probiotic to alleviate amyloid accumulation and cognitive impairments in AD rats. Mol. Nutr. Food Res. 66:2200265. 10.1002/mnfr.202200265 35975737

[B474] Wang Y. Yin Y. Chen X. Zhao Y. Wu Y. Li Y.. (2019). Induction of intestinal Th17 cells by flagellins from segmented filamentous bacteria. Front. Immunol. 10:2750. 10.3389/fimmu.2019.02750 31824516 PMC6883716

[B475] Wang Z. Zhang L. Qin C. (2025). Alzheimer's disease pathogenesis: standing at the crossroad of lipid metabolism and immune response. Mol. Neurodegener. 20:67. 10.1186/s13024-025-00857-6 40468377 PMC12139291

[B476] Weis S. Schwiertz A. Unger M. M. Becker A. Faßbender K. Ratering S.. (2019). Effect of Parkinson's disease and related medications on the composition of the fecal bacterial microbiota. NPJ Parkinsons Dis. 5:28. 10.1038/s41531-019-0100-x 31815177 PMC6884491

[B477] Weng H. Deng L. Wang T. Xu H. Wu J. Zhou Q.. (2024). Humid heat environment causes anxiety-like disorder via impairing gut microbiota and bile acid metabolism in mice. Nat. Commun. 15:5697. 10.1038/s41467-024-49972-w 38972900 PMC11228019

[B478] Westall F. C. (2006). Molecular mimicry or structural mimicry? Mol. Immunol. 43, 1062–1064. 10.1016/j.molimm.2005.06.039 16054695

[B479] Wlodarska M. Luo C. Kolde R. d'Hennezel E. Annand J. W. Heim C. E.. (2017). Indoleacrylic acid produced by commensal Peptostreptococcus species suppresses inflammation. Cell Host Microbe 22, 25–37.e6. 10.1016/j.chom.2017.06.007 28704649 PMC5672633

[B480] Wolff B. S. Alshawi S. A. Feng L. R. Juneau P. L. Saligan L. N. (2021). Inflammation plays a causal role in fatigue-like behavior induced by pelvic irradiation in mice. Brain Behav. Immun. Health 15:100264. 10.1016/j.bbih.2021.100264 34589770 PMC8474574

[B481] Wu H. Chiou J. (2021). Potential benefits of probiotics and prebiotics for coronary heart disease and stroke. Nutrients 13:2878. 10.3390/nu13082878 34445037 PMC8401746

[B482] Wu J. Li M. Li A. Ji X. (2022). Mechanism of Salvia miltiorrhiza Bge. for the treatment of ischemic stroke based on bioinformatics and network pharmacology. Evid. Based Complement. Alternat. Med. 2022:1767421. 10.1155/2022/1767421 36133785 PMC9484879

[B483] Wu M. Cheng Y. Zhang R. Han W. Jiang H. Bi C.. (2024). Molecular mechanism and therapeutic strategy of bile acids in Alzheimer's disease from the emerging perspective of the microbiota–gut–brain axis. Biomed. Pharmacother. 178:117228. 10.1016/j.biopha.2024.117228 39088965

[B484] Wu P. Lin S. Cao G. Wu J. Jin H. Wang C.. (2022). Absorption, distribution, metabolism, excretion and toxicity of microplastics in the human body and health implications. J. Hazard. Mater. 437:129361. 10.1016/j.jhazmat.2022.129361 35749897

[B485] Wu Y. Li X. Ji X. Ren W. Zhu Y. Chen Z.. (2025). Trends in the epidemiology of anxiety disorders from 1990 to 2021: a global, regional, and national analysis with a focus on the sociodemographic index. J. Affect. Disord. 373, 166–174. 10.1016/j.jad.2024.12.086 39732404

[B486] Xiao H. Cui M. Li Y. Dong J. Zhang S. Zhu C.. (2020). Gut microbiota-derived indole 3-propionic acid protects against radiation toxicity via retaining acyl-CoA-binding protein. Microbiome 8:69. 10.1186/s40168-020-00845-6 32434586 PMC7241002

[B487] Xie X. Wang L. Dong S. Ge S. Zhu T. (2024). Immune regulation of the gut-brain axis and lung-brain axis involved in ischemic stroke. Neural. Regen. Res. 19, 519–528. 10.4103/1673-5374.380869 37721279 PMC10581566

[B488] Xiong R. Gunter C. Fleming E. Vernon S. D. Bateman L. Unutmaz D.. (2023). Multi-'omics of gut microbiome-host interactions in short- and long-term myalgic encephalomyelitis/chronic fatigue syndrome patients. Cell Host Microbe 31, 273–287.e5. 10.1016/j.chom.2023.01.001 36758521 PMC10353054

[B489] Xiong R.-G. Li J. Cheng J. Zhou D.-D. Wu S.-X. Huang S.-Y.. (2023). The role of gut microbiota in anxiety, depression, and other mental disorders as well as the protective effects of dietary components. Nutrients 15:3258. 10.3390/nu15143258 37513676 PMC10384867

[B490] Xue C. Ge Y. Tang B. Liu Y. Kang P. Wang M.. (2015). A Meta-analysis of risk factors for combat-related PTSD among military personnel and veterans. PLoS ONE 10:e0120270. 10.1371/journal.pone.0120270 25793582 PMC4368749

[B491] Yadav S. K. Ahmad R. Moshfegh C. M. Sankarasubramanian J. Joshi V. Elkhatib S. K.. (2023). Repeated social defeat stress induces an inflammatory gut milieu by altering the mucosal barrier integrity and gut microbiota homeostasis. Biol. Psychiatry Glob. Open Sci. 3, 824–836. 10.1016/j.bpsgos.2023.03.005 37881577 PMC10593959

[B492] Yan J. Chen H. Zhang Y. Peng L. Wang Z. Lan X.. (2024). Fecal microbiota transplantation significantly improved respiratory failure of amyotrophic lateral sclerosis. Gut Microbes 16:2353396. 10.1080/19490976.2024.2353396 38778483 PMC11123505

[B493] Yang C. Qu Y. Fujita Y. Ren Q. Ma M. Dong C.. (2017). Possible role of the gut microbiota–brain axis in the antidepressant effects of (R)-ketamine in a social defeat stress model. Transl. Psychiatry 7:1294. 10.1038/s41398-017-0031-4 29249803 PMC5802627

[B494] Yang J.-Z. Zhang K.-K. Liu Y. Li X.-W. Chen L.-J. Liu J.-L.. (2023). Epigallocatechin-3-gallate ameliorates polystyrene microplastics-induced anxiety-like behavior in mice by modulating gut microbe homeostasis. Sci. Total Environ. 892:164619. 10.1016/j.scitotenv.2023.164619 37269995

[B495] Yang Q. Qin B. Hou W. Qin H. Yin F. (2023). Pathogenesis and therapy of radiation enteritis with gut microbiota. Front. Pharmacol. 14:1116558. 10.3389/fphar.2023.1116558 37063268 PMC10102376

[B496] Yao Y. Qi X. Jia Y. Ye J. Chu X. Wen Y.. (2023). Evaluating the interactive effects of dietary habits and human gut microbiome on the risks of depression and anxiety. Psychol. Med. 53, 3047–3055. 10.1017/S0033291721005092 35074039

[B497] Yassin L. K. Nakhal M. M. Alderei A. Almehairbi A. Mydeen A. B. Akour A.. (2025). Exploring the microbiota-gut-brain axis: impact on brain structure and function. Front. Neuroanat. 19:1504065. 10.3389/fnana.2025.1504065 40012737 PMC11860919

[B498] Yi Y. Lu W. Shen L. Wu Y. Zhang Z. (2023). The gut microbiota as a booster for radiotherapy: novel insights into radio-protection and radiation injury. Exp. Hematol. Oncol. 12, 48. 10.1186/s40164-023-00410-5 37218007 PMC10201781

[B499] Yin J. Liao S.-X. He Y. Wang S. Xia G.-H. Liu F.-T.. (2015). Dysbiosis of gut microbiota with reduced trimethylamine-N-oxide level in patients with large-artery atherosclerotic stroke or transient ischemic attack. J. Am. Heart Assoc. 4:e002699. 10.1161/JAHA.115.002699 26597155 PMC4845212

[B500] Yirmiya K. Turjeman S. Shtossel O. Zagoory-Sharon O. Moadi L. Rubin E.. (2024). Microbiome signature of posttraumatic stress disorder and resilience in youth. Psychol. Trauma Theory Res. Pract. Policy 16, 645–657. 10.1037/tra0001727 39023942

[B501] Yohn C. N. Gergues M. M. Samuels B. A. (2017). The role of 5-HT receptors in depression. Mol. Brain 10:28. 10.1186/s13041-017-0306-y 28646910 PMC5483313

[B502] Yolken R. Adamos M. Katsafanas E. Khushalani S. Origoni A. Savage C.. (2016). Individuals hospitalized with acute mania have increased exposure to antimicrobial medications. Bipolar Disord. 18, 404–409. 10.1111/bdi.12416 27425597 PMC5508736

[B503] Zang Y. Lai X. Li C. Ding D. Wang Y. Zhu Y. (2023). The role of gut microbiota in various neurological and psychiatric disorders-an evidence mapping based on quantified evidence. Mediators Inflamm. 2023:5127157. 10.1155/2023/5127157 36816743 PMC9936509

[B504] Zangerolamo L. Carvalho M. Barssotti L. Soares G. M. Marmentini C. Boschero A. C.. (2022). The bile acid TUDCA reduces age-related hyperinsulinemia in mice. Sci. Rep. 12:22273. 10.1038/s41598-022-26915-3 36564463 PMC9789133

[B505] Zhan X. Stamova B. Jin L.-W. DeCarli C. Phinney B. Sharp F. R. (2016). Gram-negative bacterial molecules associate with Alzheimer disease pathology. Neurology 87, 2324–2332. 10.1212/WNL.0000000000003391 27784770 PMC5135029

[B506] Zhang J. Liu K. Sun L. Yang L. Liu X. Zhu Y.. (2021a). Exposure to antibiotics and mental disorders in children: a community-based cross-sectional study. Environ. Geochem. Health 43, 3237–3253. 10.1007/s10653-021-00840-2 33547614

[B507] Zhang J. Wang L. Trasande L. Kannan K. (2021b). Occurrence of polyethylene terephthalate and polycarbonate microplastics in infant and adult feces. Environ. Sci. Technol. Lett. 8, 989–994. 10.1021/acs.estlett.1c00559

[B508] Zhang L. Wang Y. Xiayu X. Shi C. Chen W. Song N.. (2017). Altered gut microbiota in a mouse model of Alzheimer's disease. JAD 60, 1241–1257. 10.3233/JAD-170020 29036812

[B509] Zhang W. Dong X. Y. Huang R. (2023). Gut microbiota in ischemic stroke: role of gut bacteria-derived metabolites. Transl. Stroke Res. 14, 811–828. 10.1007/s12975-022-01096-3 36279071

[B510] Zhang Y. Dong Y. Lu P. Wang X. Li W. Dong H.. (2021c). Gut metabolite Urolithin A mitigates ionizing radiation-induced intestinal damage. J. Cell. Mol. Med. 25, 10306–10312. 10.1111/jcmm.16951 34595829 PMC8572803

[B511] Zhang Y. Hu J. Song X. Dai J. Tang Z. Huang G.. (2023). The effects of Lactobacillus reuteri microcapsules on radiation-induced brain injury by regulating the gut microenvironment. Food Funct. 14, 10041–10051. 10.1039/D3FO0C 37843434

[B512] Zhang Y. Wang Z. Peng J. Gerner S. T. Yin S. Jiang Y. (2021d). Gut microbiota-brain interaction: an emerging immunotherapy for traumatic brain injury. Exp. Neurol. 337:113585. 10.1016/j.expneurol.2020.113585 33370556

[B513] Zhao L. Zhang F. Ding X. Wu G. Lam Y. Y. Wang X.. (2018). Gut bacteria selectively promoted by dietary fibers alleviate type 2 diabetes. Science 359, 1151–1156. 10.1126/science.aao5774 29590046

[B514] Zhao Y. Cong L. Jaber V. Lukiw W. J. (2017). Microbiome-derived lipopolysaccharide enriched in the perinuclear region of Alzheimer's disease brain. Front. Immunol. 8:1064. 10.3389/fimmu.2017.01064 28928740 PMC5591429

[B515] Zhao Z. Ning J. Bao X.-Q. Shang M. Ma J. Li G.. (2021). Fecal microbiota transplantation protects rotenone-induced Parkinson's disease mice via suppressing inflammation mediated by the lipopolysaccharide-TLR4 signaling pathway through the microbiota-gut-brain axis. Microbiome 9:226. 10.1186/s40168-021-01107-9 34784980 PMC8597301

[B516] Zheng Y. Bonfili L. Wei T. Eleuteri A. M. (2023). Understanding the gut–brain axis and its therapeutic implications for neurodegenerative disorders. Nutrients 15:4631. 10.3390/nu15214631 37960284 PMC10648099

[B517] Zheng Y. Xu S. Liu J. Liu Z. (2024). The effects of micro- and nanoplastics on the central nervous system: a new threat to humanity? Toxicology 504:153799. 10.1016/j.tox.2024.153799 38608860

[B518] Zhou J. Tang M. Li W. Fang R. Tang C. Wang Q. (2024). Diet and physical activity influence the composition of gut microbiota, benefit on Alzheimer's disease. Food Sci. Human Wellness 13, 541–555. 10.26599/FSHW.2022.9250049

[B519] Zhou Q. Sun T. Wu F. Li F. Liu Y. Li W.. (2020). Correlation of gut microbiota and neurotransmitters in a rat model of post-traumatic stress disorder. J. Traditional Chin. Med. Sci. 7, 375–385. 10.1016/j.jtcms.2020.10.005

[B520] Zhou S.-Y. Guo Z.-N. Yang Y. Qu Y. Jin H. (2023). Gut-brain axis: mechanisms and potential therapeutic strategies for ischemic stroke through immune functions. Front. Neurosci. 17:1081347. 10.3389/fnins.2023.1081347 36777635 PMC9911679

[B521] Zhou X.-Y. (2016). Visceral hypersensitive rats share common dysbiosis features with irritable bowel syndrome patients. WJG 22:5211. 10.3748/wjg.v22.i22.5211 27298564 PMC4893468

[B522] Zhu G. Zhao J. Wang G. Chen W. (2023). *Bifidobacterium breve* HNXY26M4 attenuates cognitive deficits and neuroinflammation by regulating the gut–brain axis in APP/PS1 mice. J. Agric. Food Chem. 71, 4646–4655. 10.1021/acs.jafc.3c00652 36888896

[B523] Zhu G. Zhao J. Zhang H. Chen W. Wang G. (2021). Administration of *Bifidobacterium breve* improves the brain function of Aβ1-42-treated mice via the modulation of the gut microbiome. Nutrients 13:1602. 10.3390/nu13051602 34064762 PMC8150793

[B524] Zhu R. Fang Y. Li H. Liu Y. Wei J. Zhang S.. (2023). Psychobiotic *Lactobacillus plantarum* JYLP-326 relieves anxiety, depression, and insomnia symptoms in test anxious college via modulating the gut microbiota and its metabolism. Front. Immunol. 14:1158137. 10.3389/fimmu.2023.1158137 37033942 PMC10077425

[B525] Zhuang Z.-Q. Shen L.-L. Li W.-W. Fu X. Zeng F. Gui L.. (2018). Gut microbiota is altered in patients with Alzheimer's disease. JAD 63, 1337–1346. 10.3233/JAD-180176 29758946

[B526] Zoladz P. R. Del Valle C. R. Smith I. F. Goodman C. S. Dodson J. L. Elmouhawesse K. M.. (2021). Glucocorticoid abnormalities in female rats exposed to a predator-based psychosocial stress model of PTSD. Front. Behav. Neurosci. 15:675206. 10.3389/fnbeh.2021.675206 34220463 PMC8249699

